# Genomic Analysis of Terpene Synthase Family and Functional Characterization of Seven Sesquiterpene Synthases from *Citrus sinensis*

**DOI:** 10.3389/fpls.2017.01481

**Published:** 2017-08-24

**Authors:** Berta Alquézar, Ana Rodríguez, Marcos de la Peña, Leandro Peña

**Affiliations:** ^1^Laboratório de Biotecnologia Vegetal, Pesquisa y Desenvolvimento, Fundo de Defesa da Citricultura Araraquara, Brazil; ^2^Instituto de Biología Molecular y Celular de Plantas, Consejo Superior de Investigaciones Científicas and Universidad Politécnica de Valencia Valencia, Spain

**Keywords:** citrus, orange, volatile, monoterpene, sesquiterpene, defense, β-caryophyllene

## Abstract

Citrus aroma and flavor, chief traits of fruit quality, are derived from their high content in essential oils of most plant tissues, including leaves, stems, flowers, and fruits. Accumulated in secretory cavities, most components of these oils are volatile terpenes. They contribute to defense against herbivores and pathogens, and perhaps also protect tissues against abiotic stress. In spite of their importance, our understanding of the physiological, biochemical, and genetic regulation of citrus terpene volatiles is still limited. The availability of the sweet orange (*Citrus sinensis* L. Osbeck) genome sequence allowed us to characterize for the first time the terpene synthase (TPS) family in a citrus type. *CsTPS* is one of the largest angiosperm TPS families characterized so far, formed by 95 loci from which just 55 encode for putative functional TPSs. All TPS angiosperm families, TPS-a, TPS-b, TPS-c, TPS-e/f, and TPS-g were represented in the sweet orange genome, with 28, 18, 2, 2, and 5 putative full length genes each. Additionally, sweet orange β-farnesene synthase, (*Z*)-β-cubebene/α-copaene synthase, two β-caryophyllene synthases, and three multiproduct enzymes yielding β-cadinene/α-copaene, β-elemene, and β-cadinene/ledene/allo-aromandendrene as major products were identified, and functionally characterized via *in vivo* recombinant *Escherichia coli* assays.

## Introduction

*Citrus* belongs to the family Rutaceae, subfamily Aurantioideae, members of which are cultivated worldwide, being the first fruit crop in international trade in terms of value^1^. From a commercial point of view, there are seven main horticultural groups within citrus: sweet oranges (*Citrus sinensis* L. Osb.), mandarins (*Citrus reticulata* Blanco), pummelos [*Citrus maxima* (Burm.) Merr.], and grapefruits (*Citrus paradisi* Macf.), and the common acid members, which include three subgroups: citrons (*Citrus medica* L.), lemons (*Citrus limon* L. Burm. f.), and limes [*Citrus aurantifolia* (Christm.) Swing.; *Citrus latifolia* Tan.]. They have long been recognized by consumers for its flavor, health, and nutritional properties and they are consumed worldwide usually as fresh fruit or processed products such as juice, marmalade, etc. Citrus trees are also notable for their fragrance and they are used as sources of flavors and fragrances in cosmetics, perfumes, soft drinks, and food industries. Besides, as aroma is one of the most appreciated fruit characteristics, volatile compounds play a key role in determining the perception, and acceptability of citrus products by consumers (Rodríguez et al., 2017).

Citrus fragrance shows genotypic variation, ranging from the light aroma of limes to the sweet of oranges and to the pungent aroma of citron (Rouseff et al., 2009; Azam et al., 2013a). Different cultivars as well as their different organs also present distinctive aroma profiles (Cheong et al., 2012; Azam et al., 2013a; Rodríguez et al., 2013; Zhong et al., 2014; Ren et al., 2015), which are influenced as well by developmental factors (Azam et al., 2013b; Rodríguez et al., 2013). Over 100 volatile organic compounds (VOCs) have been identified in *Citrus, being* terpene (mono- and sesquiterpene) hydrocarbons, and their oxygenated derivatives, alcohols, aldehydes, esters, ethers, and oxides, the main framework of the entire aroma (Dugo, 2002; González-Mas et al., 2011; Azam et al., 2013b; Rodríguez et al., 2013; Zhong et al., 2014). They accumulate in specialized oil glands in the leaf and flavedo (outer part of the peel) and in oil bodies in the fruit juice sacs. The role of terpenes in plant defense against herbivores and plant pathogens or as attractants for pollinators and natural enemies of pest insects has been demonstrated (Holopainen, 2004). In rough lemon, monoterpenes emitted from leaves increase as response to wounding, and microbial infection (Yamasaki et al., 2007; Shishido et al., 2012), and the expression level of some terpene synthase (TPS) genes are increased by microbial attack (Shishido et al., 2012; Shimada et al., 2014). In citrus peel, high D-limonene content has been related with the attraction of a citrus pest (the medfly *Ceratitis capitata*) and the infection by specialized citrus pathogens (Rodríguez et al., 2011, 2015).

Despite the relevance of terpenes in citrus, their biosynthesis has remained elusive until recently. Terpene biosynthesis starts with one isoprene unit, C_5_ isopentenyl pyrophosphate (IPP) and its isomer dimethylallyl diphosphate (DMAPP), through serial additions of this unit to reach the formation of geranyl diphosphate (GPP), farnesyl diphosphate (FPP), and geranyl geranyl diphosphate (GGPP) via elongation reactions. In plants, monoterpenes (C10-based) are synthesized from the plastid-derived GPP, and sesquiterpenes (C15-based) from the cytoplasmically derived FPP. The conversions of these precursors are carried out by a large family of enzymes known as TPSs, to produce a vast array of terpenoids (Tholl, 2015). According to their genomic structure, including intron number, exon size, and intron position, *TPSs* can be classified into three classes: 12–14 introns (class I), 9 introns (class II), or 6 introns (class III) (Trapp and Croteau, 2001). In general, TPS proteins are 550–850 amino acids length and their molecular masses are about 50–100 kDa. Monoterpene synthases (MonoTPSs) are in the range of 600–650 amino acids and bear an N-terminal transit plastid, in accordance with monoterpene biosynthesis in plastids. As sesquiterpene biosynthesis takes place in the cytosol, sesquiterpene synthases (SesquiTPSs) lack plastid targeting signals, and they are typically about 50–70 amino acids shorter than MonoTPSs. Although a high number of TPS are supposed to be present in the citrus genome (Jia et al., 2005), just a few have been cloned from citrus. From the peel of lemon fruits, Lücker et al. (2002) isolated two D-limonene synthases, which also produced minute amounts of β-myrcene and α-pinene, and two other enzymes less specific in their product formation, which generate a major product (γ-terpinene or β-pinene) and up to 11% of other monoterpenes. A γ-terpinene synthase and a sabinene synthase producing other side compounds were isolated from Satsuma mandarin [*Citrus unshiu* (Mak.) Marc.] and rough lemon (*Citrus jambhiri* Lush.), respectively (Suzuki et al., 2004; Kohzaki et al., 2009). Also from Satsuma mandarin, different D-limonene and linalool synthases were characterized (Shimada et al., 2004, 2014). A single-product valence synthase was isolated from Valencia sweet orange (Sharon-Asa et al., 2003). Uji et al. (2015) isolated a TPS producing δ-elemene or β-myrcene when FPP or GPP were used as substrates, respectively, indicating that the TPS activity of this enzyme is strictly determined by subcellular localization. Similarly, CuSTS4 produced linalool or nerolidol depending on the substrate provided (Shimada et al., 2014). Thus, in general, the product(s) of a given TPS cannot be predicted from an analysis of its homology with identified TPS sequences, and it has been suggested that the product specificity of Mono/SesquiTPS depends on a critical region rather than the entire structure. For example, β-pinene synthases of lemon, and Satsuma mandarin (Lücker et al., 2002; Shimada et al., 2004) have 95 and 97% sequence identity, respectively, with the sabinene synthase isolated from rough lemon (Kohzaki et al., 2009), though the last, because of a single amino acid substitution, could not produce β-pinene dominantly (Kohzaki et al., 2009).

Considering the importance of terpenes in citrus and the limited amount of knowledge that is currently available about their biosynthesis, the objective of this work was to characterize the TPS family in citrus, taking advantage of the enormous amount of information available as a result of the citrus genomes sequence projects (Xu et al., 2013; Wang et al., 2014). Special interest has been devoted to the characterization of SesquiTPS, because only four enzymes have been characterized so far from citrus with this type of activity.

## Materials and methods

### Plant material and volatile emission GC-MS analysis

Fruit at four developmental stages (immature green, mature green, breaker, and full colored), leaves at two developmental stages (young and mature), and open flowers of Pineapple sweet orange (*C. sinensis* L. Osb.) were harvested at random from adult trees grafted on Carrizo citrange (*C. sinensis* L. Osb. × *Poncirus trifoliata* L. Raf.) rootstocks cultivated at Instituto Valenciano de Investigaciones Agrarias (Moncada, Valencia, Spain). Fruit peel and pulp were separated with a scalpel and immediately frozen in liquid nitrogen. Flowers and leaves were also immediately frozen. Frozen material was ground to a fine powder and stored at −80°C until RNA extraction.

Volatile emission analysis was performed by static headspace sampling with a solid phase microextraction (SPME) essentially as described previously (Peris et al., 2017). For pulp volatile analysis, 100 mg of grounded tissue were transferred to a 10 mL Teflon sealed glass tube and homogenized with 300 μL of a saturated NaCl solution. After 30 min of incubation at 30°C with slow agitation, SPME fiber was exposed for 20 min. For fresh flowers or leaves, analysis was performed by enclosing intact whole organs in 10 or 50 mL screw-cap Pyrex tubes carrying a septum on the top and containing 1 mL of milli-Q water for avoiding hydric stress, and incubated at a controlled temperature of 22°C between 1 and 4 h. Fresh fruits were enclosed in 1 L glass jars using 3M™ aluminum foil and incubated at 22°C for 5 h. After incubation, SPME fiber (100 μm poly(dimethyl) siloxane, Supelco, Bellefonte, PA) was exposed for 30 min at 22°C and immediately afterwards transferred to GC injector (220°C) where thermal desorption was prolonged to 4 min. Subsequent GC-MS analysis was conducted as described above.

GC-MS analysis was carried out as described before (Peris et al., 2017). α-farnesene, α-humulene, α-farnesene, α-pinene, α-copaene, β-caryophyllene, α-terpinene, D-limonene, decanal, geranyl acetate, and linalool were identified by comparison with available commercial standards. Remaining compounds were tentatively identified by matching of the acquired mass spectra with those stored in NIST reference library for GC-MS.

### Identification and sequence analysis of *CsTPSs*

In order to identify putative *TPS* genes in sweet orange, BLASTp searches in the *Citrus sinensis* Annotation Project (CAP^2^) were performed using as queries the keywords “linalool” and “limonene” (two abundant monoterpenes in citrus). In addition, in order to be not-dependent of the automatic annotation, the database was screened (BLASTp) with known functional monoterpene and sesquiterpene protein sequences from citrus: BAD91045, and AAQ04608, annotated in NCBI database as 1,8-cineole synthase from *Citrus unshiu*, and valencene synthase from *Citrus sinensis*, respectively. After removing duplicated sequences, a total of 95 putative *TPS* gene models were identified in the sweet orange genome. NCBI ORF finder^3^ was used to reveal the putative ORFs of the 111 predicted *TPS* transcripts and those encoding predicted proteins of at least 400 amino acids were further analyzed by homology search in non-redundant protein database from NCBI. As lot of information about citrus genomes is available (i.e., orange and clementine genomes^1^,^4^,^5^) the best matches corresponded in most cases to citrus genes automatically annotated. In order to avoid possible annotation mistakes from massive computational analysis, those matches corresponding to *Citrus* or citrus-related types were ignored, and as best match the first non-citrus protein was selected. Motif families were identified by searching in the pFAM^6^ database (Finn et al., 2016).

Intron/exon structure of putative citrus *TPS* genes was determined by manual analysis of the coding region, including evaluation of intron number, length (nucleotides), placement, and phase defined as the location of the intron before the first, second, or third nucleotide position of the proximate codon, and was referred to as phase 0, 1, or 2, respectively, and exon number and size (amino acids). The architecture of each *CsTPS* was summarized in table format, where conserved motifs were also annotated, and a physical map (exon and intron) of each gene was created. *TPS* architectural maps were aligned by hand with the assistance of Adobe Illustrator 6.0 and then compared. The classification of *TPS* genes into class I, II, or III types was based upon grouping by physical similarities of gene architectures as described previously (Trapp and Croteau, 2001). Consensus sequences for exon–intron and intron–exon boundaries were determined using WebLogo server^7^.

Molecular weight and isoelectric point of predicted proteins with good homology to previously annotated TPSs were calculated with ExPasy^8^. Subcellular location was predicted by the ChloroP^9^ and TargetP^10^ programs. Multiple sequence alignment of *CsTPS* genes was performed using ClustaX (version 1.81). Phylogenetic tree was constructed using the MEGA 4.1 program with the neighbor-joining (NJ) method (Saitou and Nei, 1987) and bootstrap analysis (1,000 replicates).

### RNA isolation, cDNA synthesis, SesquiTPS isolation, and expression analysis

Total RNA from plant tissues was isolated as previously described (Rodrigo et al., 2004). Total RNA (1 μg) was reversed transcribed using 200 U of SuperScript II Reverse Transcriptase (Invitrogen) and 500 ng of oligodT primer. Full length cDNA clones were amplified from a 1:1 mix of different plant tissues (leaves, peel, pulp, and flowers) using KAPA HiFi polymerase (KAPA Biosystems) and gene-specific primer pairs (Supplementary Table 1). PCR products were cloned into pJET1.2/blunt (ThermoFisher Scientific) according to manufacturer instructions and at least 10 clones per gene were fully sequenced. Identity of the clones was confirmed by blasting their sequences to the CAP database. Same primer pairs and 25 amplification cycles with CloneAmp HiFi polymerase were employed for non-quantitative expression analysis (Clontech). Using CAP^2^ (Wang et al., 2014) accession number RNA-seq data for each identified putative full length *CsTPS* gene were downloaded and used to analyze their expression levels in flower, leaf, and fruit tissues. A heat-map was generated using ClustVis^11^ (Metsalu and Vilo, 2015).

### Functional analysis of CsTPSs

The complete coding sequence of *Cs4g11980, Cs4g12120, Cs4g12350, Cs4g12400, Cs5g23510, orange1.1t03302*, and *orange1.1t04360* was amplified from pJET1.2/blunt cloning vector using specific primers (Supplementary Table 1), and KAPA HiFi polymerase (KAPA Biosystems), and amplicons were subcloned into the bacterial expression vector pET-45b(+) (Novagen) using Infusion HD cloning kit (Clontech) following manufacturer instructions. One microliter of the reaction mix was used to transform *Escherichia coli DH5*α competent cells. Positive colonies were selected and fully sequenced to assess identity and discard errors introduced by DNA amplification.

Selected clones and pET-45b(+) empty vector as control were transformed to *E. coli* BL21(DE3) strain (Invitrogen, Carlsbad, CA, USA). Plasmid DNA was isolated and re-sequenced to check identity. For induction of protein expression, single colonies were used to inoculate 5 mL LB broth supplemented with ampicillin (100 μg/mL) and grown overnight at 37°C and 200 r.p.m. Aliquots of 100 μL were used to inoculate 250 mL Erlenmeyer flasks containing 100 mL of LB broth supplemented with carbenicillin (50 μg/mL). Cultures were grown at 28°C and 200 r.p.m. to an OD_600_ of 0.4.

For induction of recombinant protein expression, IPTG (isopropyl β-D-thiogalacto-pyranoside) was added to a final concentration of 1 mM and the cultures were maintained at 18°C and 80 r.p.m overnight. Aliquots of 500 μL were taken to confirm induction of recombinant proteins by SDS-PAGE and remaining cells were harvested by centrifugation for 10 min at 4,000 r.p.m. Pellets were resuspended in 4 mL of chilled SSAB buffer (100 mM NaPhosphate pH 7.0, 10 mM MgCl_2_, 1 mM DTT, 10% (v/v) glycerol; Prosser et al., 2002) and disrupted by 3 × 10 s treatments with a ultrasonic processor (UP200S, Hielscher). Cell debris was removed by centrifugation for 30 min at 15,000 r.p.m. Aliquots of 500 μL of the supernatant, which contained the expressed soluble proteins, were transferred to 3 mL Teflon sealed glass tubes, supplemented with 10 μg of (*E*,*E*)-farnesyl-diphosphate (FPP, Sigma), and incubated for 2 h at 30°C and 50 r.p.m. A SPME fiber [100 μm poly(dimethyl) siloxane, Supelco, Bellefonte, PA] was placed into the headspace of the tube during 45-min at 22°C and immediately the fiber was transferred to GC injector (220°C) and thermal desorption was prolonged to 4 min GC-MS conditions were set as described above.

### Molecular modeling

The search for templates in PDB proteins database revealed (+)-δ-cadinene synthase from *Gossypum arboreum* (Protein bank code 3G4D_A; Gennadios et al., 2009) as the best match to model Cs4g12400, orange1.1t04360, and Cs4g11980 (confidence 100%; match >50%; coverage 0.99). 3D-protein structural models were obtained with the SWISS-MODEL server in Autoated Mode (Biasini et al., 2014). Structural figures were drawn using PyMol^12^.

## Results

### Sweet orange sesquiterpene emission profile

Volatile profile in sweet orange leaves, flowers, and fruits at various phenological stages was investigated by static headspace sampling, and gas chromatography-mass spectrometry. Separated volatile analysis for peel and pulp tissues from mature fruit were performed. In total, 99 volatile compounds, including 36 (plus 6 acetate/formate derivates) monoterpenes, were identified. In all samples, analyzed volatile emission was dominated by this class of terpenes, which constituted between 13 and more than 90% of the total emission (Supplementary Table 2). In general, β-terpinene, β-ocimene, and D-limonene were major compounds in all tissues, although it is interesting to note the high percentage of linalool in the volatile profile of sweet orange flowers.

**Figure 1:**
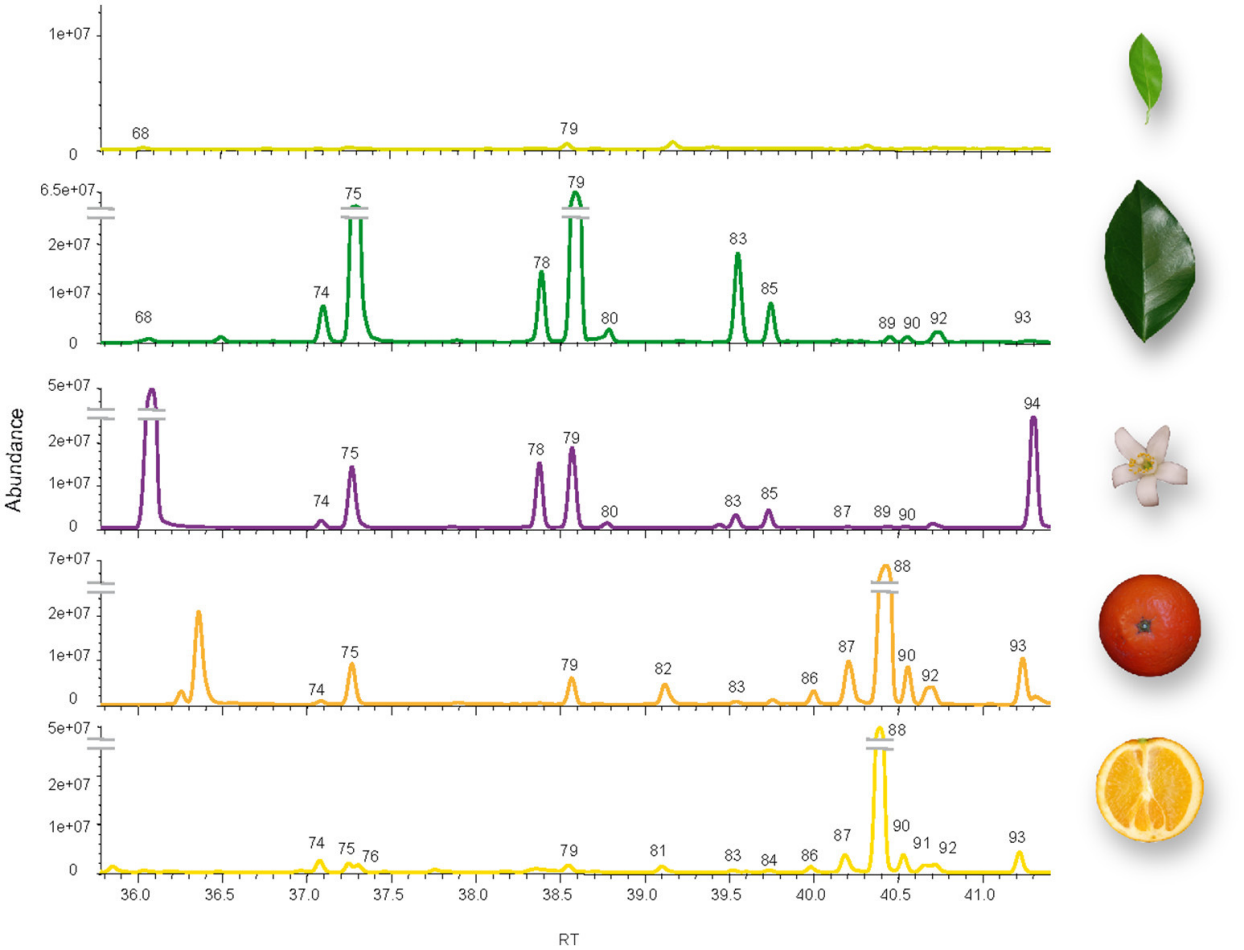
Volatile sesquiterpenes emitted by different tissues of Pineapple sweet orange. From top to bottom, young flushes, mature leaves, flowers, peel, and pulp from mature fruit. Peak numbers were assigned in basis of retention time (Supplementary Table 2), and those displayed in this figure correspond to: (68), α-cubebene; (74), α-copaene; (75), β-elemene; (76), β-cubebene; (78), (*Z*)-β-farnesene; (79), β-caryophyllene; (80), β-cedrene; (81), allo-aromadendrene; (82), γ-gurjunene; (83), α-humulene; (84), γ-selinene; (85), α-farnesene; (86), 3,7(11)-selinadiene; (87), β-selinene; (88), valencene; (89), eremophilene; (90), α-selinene; (91), δ-selinene; (92), β-cadinene, (93), α-panasinsene, and (94), (±)-trans-nerolidol.

Despite their quantitative low contribution to total volatile emission from sweet orange tissues (<18%, Supplementary Table 2), sesquiterpene profile was qualitatively rich (Figure 1). Overall, up to 25 sesquiterpenes were identified in total. Small amounts of β-caryophyllene were detected in all samples, while α-copaene, β-elemene, α-humulene, and α-selinene were emitted by all tissues except young leaves. Beside this, each tissue showed a characteristic sesquiterpene emission profile. Major sesquiterpenes emitted by adult leaves were β-elemene and β-caryophyllene, while flowers emitted nerolidol, absent from most tissues analyzed. In fruit tissues, valencene emission contributed greatly to total emitted volatiles, in much more extent in peel than in pulp.

### Identification of the *TPS* gene family in the sweet orange genome

**Figure 2:**
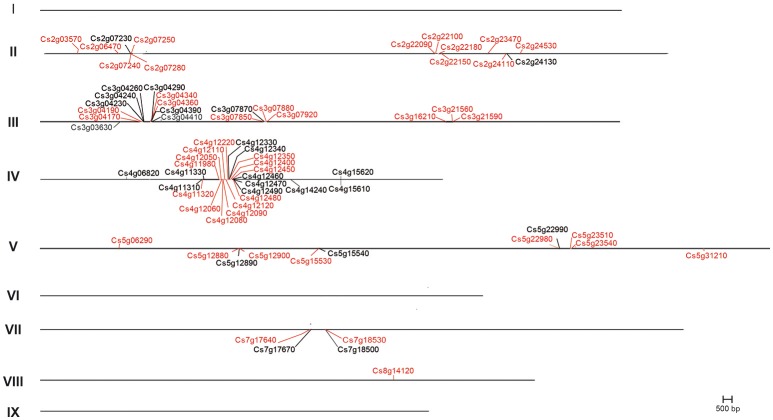
Graphic representation of chromosomic distribution of citrus *TPS* gene models in the sweet orange genome. Genes whose names begin with Cs2g, Cs3g, Cs4g, Cs5g, Cs7g, and Cs8g are located on chromosomes 2, 3, 4, 5, 7, and 8 respectively. In chromosomes 1, 6, and 9 it is not predicted the localization of any *TPS*. Twenty four genes could not be localized to a specific chromosome. Red letters represent putative complete *TPS* genes, black letters represent putative *TPS* pseudogenes. Letters over/down the lines representing chromosomes correspond to those genes predicted in the ± strands, respectively. The exact position of each gene is indicated in Supplementary Table 3.

To identify all sweet orange genes with homology to *TPSs*, the recently released sweet orange genome sequence (CAP^1^) was screened using previously annotated *Citrus* TPSs and relevant keywords as queries. This search identified a total of 95 loci and 111 predicted transcripts, 96 of them exhibiting significant similarities with known *TPSs* (Supplementary Table 3). Most of the predicted peptides presented domains described in pFAM^5^ and CCD^13^ resources as TPS-characteristics, such as PF01397, and PF03936, described as TPS N-terminal and C-terminal domains, respectively. Besides, PLN02279 (ent-kaur-16-ene synthase), PLN02150 (TPS/cyclase), and PLN02592 (ent-copalyl diphosphate synthase) were identified. Putative *TPS* genes were located on 6 of the 9 citrus chromosomes, being a significant proportion (78.84%) of them located in chromosomes II, III, IV, and V (Figure 2), while in chromosome VIII there was just one putative *TPS* gene and none in chromosomes I, VI, and IX. Of the complete set of 95 *CsTPS* loci identified in this study, 61 (63.54%) were organized in 19 distinct clusters (distance between genes lower to 67 knt), covering from 2 to 6 *CsTPS* genes or pseudogenes, while 24 of them (named as orange1.1t) were not assigned to specific positions of the genome. 16 *TPS* loci arranged in five nearby clusters located in a region of 617 knt in chromosome IV, while isolated *TPS* genes were identified in chromosomes II, IV, V, and VIII. In chromosomes II, IV, and V, *TPS* genes were almost equally distributed in both DNA strands while in chromosome III all but one gene were located in the positive strand, and in chromosome VII all the genes were in the negative strand.

**Figure 3:**
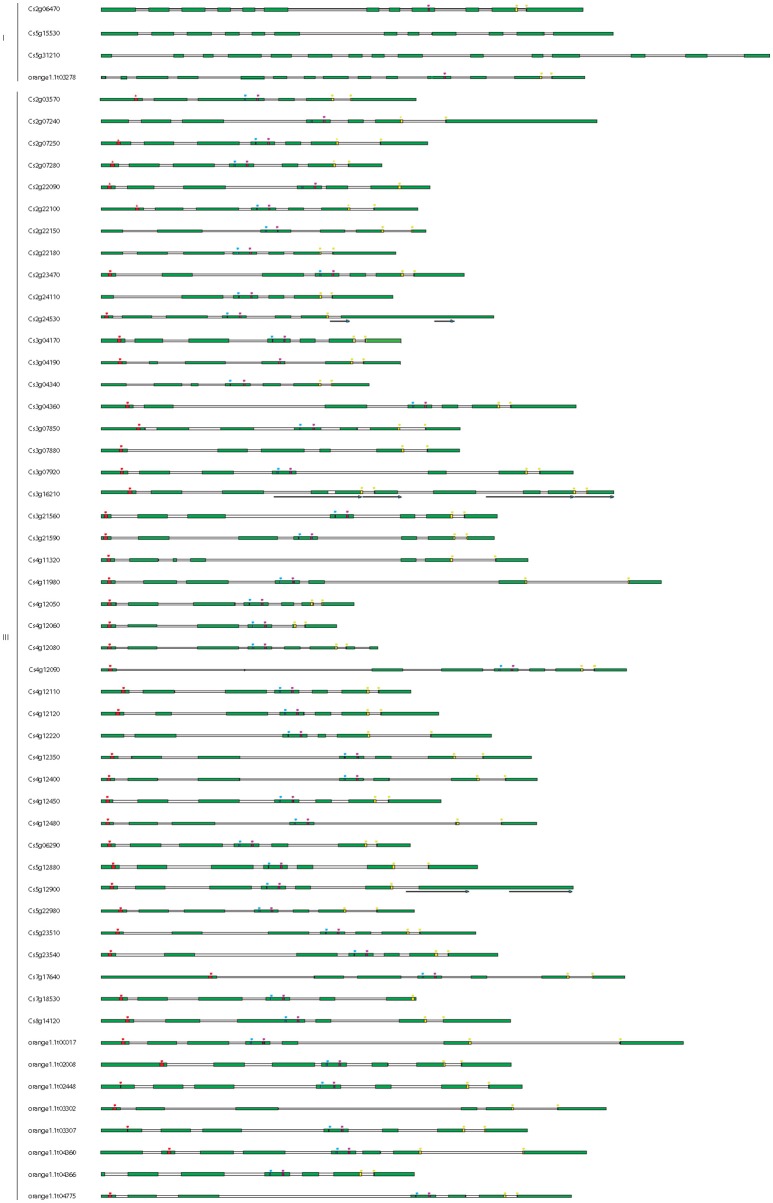
Gene intron-exon structures of the putative full length *CsTPS* genes identified from the sweet orange genome. The names of the genes are detailed on the left. Introns and exons are represented by white and green boxes, respectively. Red, blue, pink, and yellow asterisks represent conserved motifs RRX8W, RDR, DDXXD, and NSE/DTE, respectively. For more details, see Supplementary Figure 1 and Supplementary Table 5. Duplicated regions are marked by arrows.

**Figure 4:**
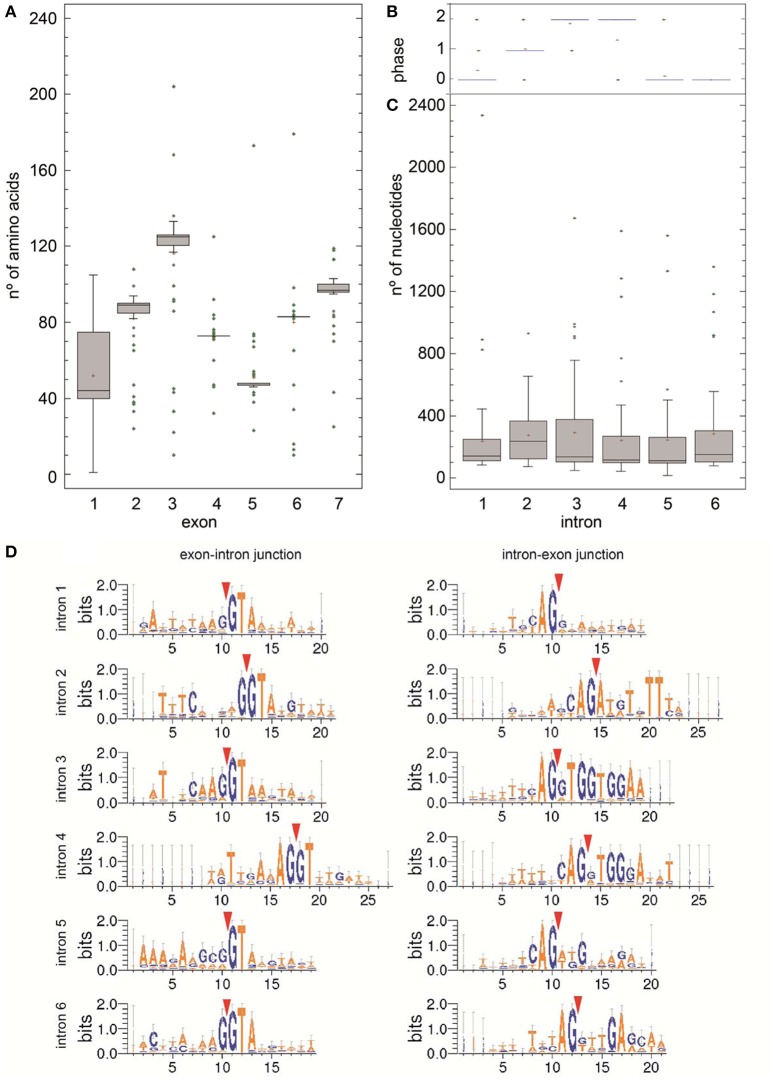
Exon and intron characteristics of putative 55 full-length *CsTPSs*. **(A)** Representation of exon length variation (in amino acids) among *CsTPSs* analyzed. **(B)** Representation of intron phase variation among *CsTPSs* analyzed. **(C)** Representation of intron length variation (in nucleotides) among *CsTPSs* analyzed. **(D)** Sequence conservation and splice position (indicated by red triangles) were calculated using WebLogo^7^ server in basis of annotated information from 55 putative *CsTPS* genes. In ordinate axis the height of the letters reflects the relative frequency of each nucleotide at each position.

Encoded protein predictions and homology analysis revealed that just 55 of the genes (70 predicted transcripts) appear to have uncompromised ORFs, encoding proteins longer than 400 amino acids and presenting significant homology to known non-citrus TPS enzymes (Supplementary Table 3). Remaining genes encoded polypeptides shorter than 400 amino acids and are likely to be pseudogenes or partial genes. Protein Cs3g21590 presented only 41% query coverage (from amino acid 1–531) to an annotated protein product from *Vitis* and to other genes annotated as *SesquiTPS*, while from amino acid 569 it presented high homology to 60S ribosomal genes. When NCBI citrus-annotated sequences were considered in homology analysis, Cs3g21590 presented 100% identity to a predicted SesquiTPS (XP_006473872) identified in the same genome annotation project that finally resulted in the CAP database. Consequently, annotated Cs3g21590 was replaced by a corrected version (Cs3g21590c) for subsequent analysis. Attending to their intron/exon number, as proposed by Trapp and Croteau (2001), *TPSs* were classified into three classes: I (12–14 introns), II (9 introns), or III (6 introns) (Figure 3). Four genes, namely *Cs2g06470, Cs5g15530, Cs5g31210*, and *orange1.1t03278*, with 12, 13, 15, and 14 predicted exons, respectively, were classified as class I, which comprises mainly diterpene synthases from both angio- and gymnosperms. Remaining genes were annotated as class III, coding for TPS-a (SesquiTPS), TPS-b (cyclic monoterpene and hemiterpene synthases) and TPS-g (acyclic MonoTPS). From these, 12 were found to be partial genes in which some part of the gene is missed, probably by deletion or incomplete duplication, while other three genes presented duplications of the 3'-end. For example, gene *Cs3g16210* had automatically predicted nine introns, a characteristic feature of class II-TPS, which is gymnosperm specific. Detailed analysis of the sequence revealed two insertions of 800 and 356 nt, corresponding to duplications from more than half of intron 3 to almost the end of exon 5, and from intron 5 to the beginning of intron 6, respectively, after exon 7. Other genes, despite suffering insertions (i.e., *orange1.1t04360*, which harbors a small insertion in exon 3), coded for putative full length TPSs (Figure 3). In total, 40 of the genes were predicted to be full-length. Manual analysis of the architecture of *CsTPS*s revealed a high conserved gene structure, with exons/introns (7/6) from class III TPSs corresponding to exons/introns 3, 8, 11, 12, 13, 14, and 15 of class I TPSs (data not shown). Number, size, placement and position of exons and introns and splice sites of all these genes have been also analyzed. The size of the exons, although to a lesser extent the first one, was quite preserved, with median lengths from 44 to 125 amino acids (Figure 4A), while intron length was highly variable, from 16 to 2,335 nucleotides, most of them being between 81 and 400 nucleotides (Figure 4C). However, conservation of intron phase was evident in all them, though with some (1–4) exceptions in all introns but 6 (Figure 4B). To further characterize *CsTPSs*, introns splicing sites context was analyzed and conserved trinucleotides R/GT and YAG/ were detected at 5′ and 3′ ends of the introns, respectively (Figure 4D).

### Protein conserved motifs and phylogenetic analysis

Overall amino acid identity among predicted citrus TPS was 42.10%, although it varied widely, from 16.92% (Cs3g07850 and Cs5g15530) to 99.81% (Cs4g12120.1 and Cs4g12120.2) (Supplementary Table 4) similarity decreasing toward the carboxyl terminus of the proteins (Supplementary Figure 1). Protein length also differed extensively, from 729–851 amino acids of class I TPS to the minimum size of class III TPS (410 amino acids for Cs4g11320, Supplementary Table 3), being the medium size for this last class of 556 amino acids.

Amino acidic sequence of putative full-length class III CsTPS was further analyzed (Supplementary Table 5). The size of the predicted protein sequences ranged from 410 to 629 amino acids, with theoretical molecular weights between 47.15 and 72.51 KDa and pIs between 4.86 and 7.63. Chloroplast transit peptides were predicted for 15 of the proteins, envisaging their probable MonoTPS activity, while the rest of CsTPSs are probably targeted to the cytosol and thus are most likely SesquiTPSs. Although all predicted full-length class III CsTPSs contained characteristic TPS motifs to a greater or lesser extent, just 39 of them (corresponding to 37 genes) contained all the motifs required for activity. At the N-terminal position, RRX8W motif essential for monoterpene synthase cyclization (Bohlmann et al., 1998; Trapp and Croteau, 2001) was completely conserved in 25 of the predicted peptides, while variations affecting the second arginine were found in 18 sequences. This motif was completely absent in nine of the predicted peptides. At the C-terminus, a fully conserved RDR motif was found in most sequences, with variations R(E/S)(Q/S) in seven of them. The aspartate rich motif involved in bivalent metal ion cofactor binding varied between CsTPSs, occurring mostly as DDIYD. Only three predicted peptides lacked this motif. NSE/DTE signature was found in all selected full-length TPSs, with the general consensus sequence (D/N)D(V/M/L/I)X(S/T)XXXE. One predicted protein (orange1.1t02008) lacked the first aspartate, while two other lacked the terminal E (Cs2g22150 and Cs7g18530). Variations at intermediate positions were found in six of the putative peptides. Interestingly, the position of all these characteristic motifs was conserved along exon structure (Figure 3, Supplementary Table 5).

**Figure 5:**
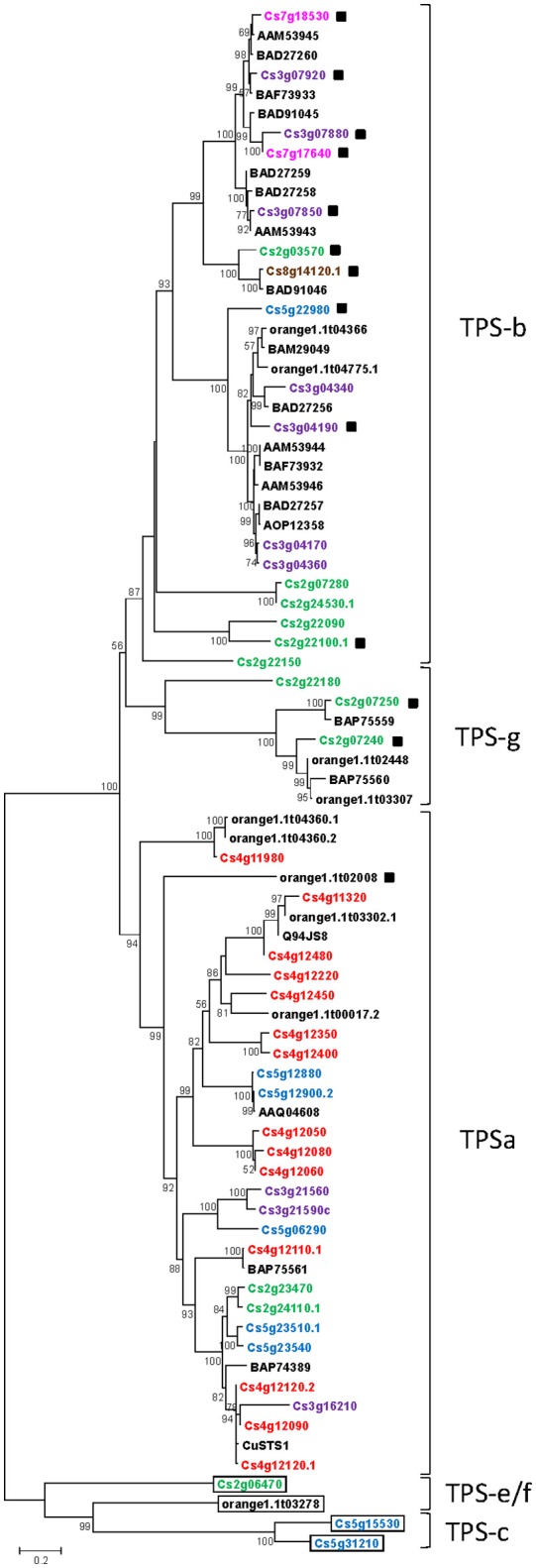
Phylogenetic analysis and chromosome location of putative and previously functionally characterized *Citrus* TPSs. Q94JS8, (E)-β-farnesene synthase from *Citrus junos*; AAQ04608, valencene synthase from *Citrus sinensis*; BAP75561, linalool synthase from *Citrus unshiu*; BAP75559, and BAP75560, linalool synthase from *Citrus unshiu*; BAF73932, and BAF73932, D-limonene synthase from *Citrus jambhiri*; BAD27256, and BAD27257, D-limonene synthase from *Citrus unshiu*; AOP12358, D-limonene synthase from *Citrus sinensis*; AAM53943, γ-terpinene synthase from *Citrus limon*; BAD27258, and BAD27259, γ-terpinene synthase from *Citrus unshiu*; AAM53944, and AAM53946, D-limonene synthase 1 from *Citrus limon*; AAM53945, β-pinene synthase from *Citrus limon*; BAM29049, geraniol synthase from *Citrus jambhiri*; BAD91046, (E)-β-ocimene synthase from *Citrus unshiu*; BAD91045, 1,8-cineole synthase from *Citrus unshiu*; BAF73933, β-pinene synthase from *Citrus jambhiri*, BAD27260, β-pinene synthase from *Citrus unshiu*; CuSTS-1, germacrene-A synthase from *Citrus unshiu*; BAP74389, δ-elemene synthase from *Citrus jambhiri*. Bootstrap values for all branches >50% are listed. Coloured letters indicate chromosome location as annotated in the CAP database (green, chromosome 2; purple, chromosome 3; red, chromosome 4; blue, chromosome 5; pink, chromosome 7; brown, chromosome 8; black, unassigned position). Black squares mark those peptides for which a transit signal to chloroplast is predicted *in silico* (Supplementary Table 5). Class I TPSs are black framed, while non-framed TPSs are predicted to belong to Class III. TPS subfamilies are designated according to Bohlmann et al. (1998).

To explore the evolutionary relationship between the citrus TPS family, putative full length functional CsTPSs identified in the CAP database, and all citrus TPSs functionally characterized up to date were used to construct a neighbor-joining phylogenetic tree (Figure 5). This analysis separated CsTPSs in two clades, one with four members and the other with the remaining sequences. The first one included TPS-c, -e, and -f subfamilies, with one or two members each. Members of these subfamilies belong to class I TPS, as envisaged from their intron number, and their larger size due to the presence of an N-terminal plant diterpene synthase conserved signature, which includes SAYDTAW and QXXDGSWG motifs (Supplementary Figure 1). Identified citrus class I TPS did not contain RRX8W, RDR, or NSE/DTE motifs. TPSc members (Cs5g15530 and Cs5g31210) did not harbored DDXXD, but DXDD. These characteristics, together with the ancestral position of this clade, suggest that CsTPSc members are most probably copalyl diphosphate synthases, in good agreement with homology analysis (Supplementary Table 3). Orange1.1t03278 and Cs2g06470 contained DDXXD signature. The first one, which constitutes by itself clade TPS-e, resembled ent-kaurene synthase from other species while Cs2g06470, only representative of clade TPS-f, presented high homology to geranyl linalool synthases annotated in the NCBI public database.

The second clade divided in two clusters, the larger one including TPS-b/g and TPS-a1 subfamilies. Identity within TPS-a1 group ranged from 28.17 (between Cs4g11320 and orange1.1t02448/orange1.1t03307) to 98.72% (between Cs5g12880 and Cs5g12900). This group contains putative SesquiTPSs and, consequently, all but one did not have a predicted transit peptide. Previously, functionally characterized citrus SesquiTPSs, such as β-farnesene synthase (Q94JS8), valencene synthase (AAQ04608), δ-elemene synthase (BAP74389), germacrene-A synthase (CuSesquiTPS1), and nerolidol/linalool synthase (BAP75561), also claded in this group. Members of Q94JS8 subcluster (Cs4g11320, orange1.1t03302.1, Cs4g12480 and Cs4g12220) maintain the DXDD motif, absent from the rest of predicted full length TPSs from class III (Supplementary Table 5). In this subfamily, arginine rich motifs presented R(P/R)X(A/S)X(F/Y)HP(S/T/N)(I/L)W and R(D/N)R consensus sequences and were found in almost all members. The first motif was absent in Cs4g12220 and Cs2g24110.1, while Cs4g11320 and orange1.1t03302 lacked RDR signature (Supplementary Table 5). Aspartate rich motifs DD(I/T)(Y/F)D and (D/N)D(I/M/V)X(S/T/G)(H/Y)(K/E)(F/V)E were found as conserved sequences in CsTPS-a, and they were present in all predicted full length proteins but Cs4g11320 and orange1.1t03302, which also lacked DDXXD.

TPS-b subfamily, related to angiosperm MonoTPSs, had 19 *C. sinensis* predicted peptides, many of them harboring plastid targeting signals (Figure 5). Minimum identity within this group was 36.67%, between Cs2g22100 and Cs3g07880, while highest was 98.68%, between Cs3g04360 and Cs3g04190. *Citrus* D-limonene (BAD27256, BAD27257, BAF73932, AAM53944, and AAM53946), geraniol (BAM29049), β-ocimene (BAD91046), γ-terpinene (AAM53943, BAD27258, and BAD27259), 1,8-cineole (BAD91045), β-pinene (AAM53945 and BAD27260) and sabinene (BAF73933) synthases reported in the literature, also claded in this group. Most (73%) predicted CsTPSb harbored all characteristic TPS motifs, presented as R(R/Q)SA(N/D)YXP(S/T/N)IW, R(D/N)(R/S), DD(I/V)YD, and (D/N)DL(G/A)TSSDE. Although the corresponding proteins from *C. sinensis* have not been functionally characterized yet, in basis of current phylogenetic analysis, and their high identity (>95% from RRX8W onwards) it could be inferred that *Cs7g18530*, and *Cs3g07850* encode sweet orange β-pinene and γ-terpinene synthases. In addition, if we compare their amino acid sequence between RDR and NSE/DTE signatures, which mostly corresponds to the catalytic pocket (Kampranis et al., 2007), the identity between these CsTPSs, and citrus characterized β-pinene and γ-terpinene synthases raises to 99%. Similarly, D-limonene synthase activity can be envisaged for Cs3g04170, and Cs3g04360, grouped with enzymes with this activity from *C. sinensis, C. limon*, and *C. unshiu*, and highly identical to them (>94% from RRX8W and >99% between conserved motifs). A second cluster of D-limonene synthases including a protein from *C. unshiu* and another putative D-limonene synthase from sweet orange (Cs3g04340) was also found.

Closely related to TPS-b family, a TPS-g cluster, corresponding to predicted acyclic MonoTPS, was found. It included five members, three of them having a predicted transit peptide to chloroplasts. All members of this cluster completely lacked RRX8W signature and poorly conserved RDR motif, being the most common version R(D/E)Q. By the other hand, DDIFD, and DDLGSAKDE were found to be absolutely conserved in all CsTPS-g, except in Cs2g22180, which presented DDLGTAREE. Cs2g22180 presented low identity (38.50% in average) with remaining sequences of the group, which showed an overall identity of 78.16%. Previously characterized citrus linalool synthases also clustered in this group (BAP75559 and BAP75560), closely related to some of the identified CsTPSs.

### *CsSesquiTPS* gene isolation and expression analysis

**Figure 6:**
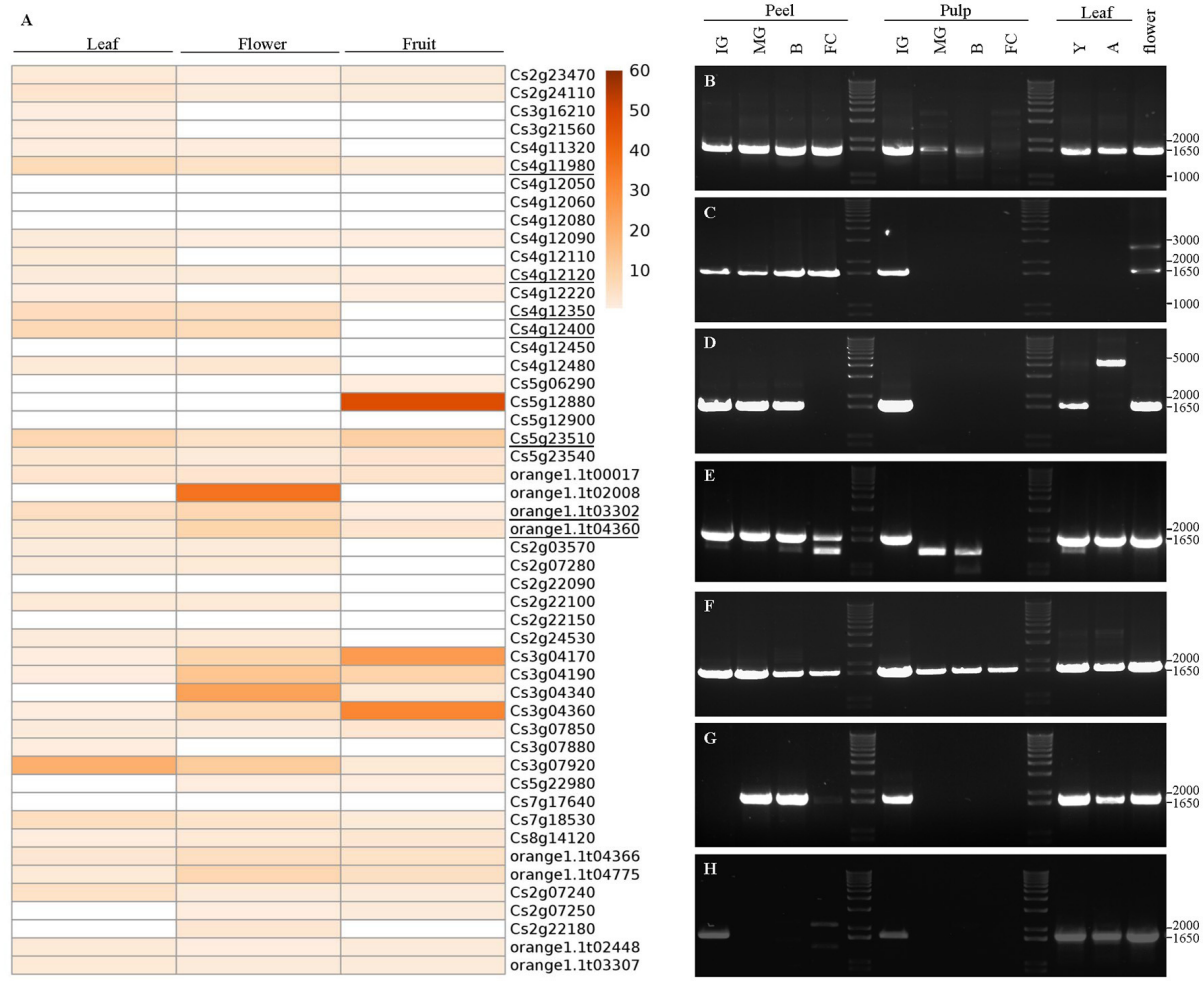
Expression of sesquiterpene synthase genes in different sweet orange tissues. **(A)** Heatmap, generated with ClustVis, showing the expression of *CsTPS* in leaf, flower and fruit tissues, according to RNA-seq data from CAP database. Genes weakly or highly expressed in the tissues are white (0.1− 0.5 reads per kilobase per million mapped reads), and red-colored (60 RPKM), respectively. The color scale on the right represents RPKM values. Genes *Cs5g23510, Cs4g12120, orange1.1t03302, Cs4g12350, Cs4g12400, orange1.1t04360*, and *Cs4g11980*, corresponding to *CsSesquiTPS 1*–*7*, respectively, are underlined. Expression of **(B)** CsSesquiTPS1, **(C)** CsSesquiTPS2, **(D)** CsSesquiTPS3, **(E)** CsSesquiTPS4, **(F)** CsSesquiTPS5, **(G)** CsSesquiTPS6, and **(H)** CsSesquiTPS7 was further investigated in peel and pulp from immature green (IG), mature green (MG), breaker **(B)**, and full colored (FC) fruit, young (Y), and adult **(A)** leaf, and flower.

In order to gain knowledge about CsTPSs, an expression analysis of putative full-length *CsTPSs* was performed using CAP RNA-seq data (Figure 6A). Resulting heatmap showed differential mRNA expression between genes. Eight genes showed low expression levels while other 22 were found to be expressed in all analyzed tissues (leaf, flower, and fruit). Four, one, and three genes showed tissue-specific expression in leaf, flower, or fruit, respectively. Most identified full length putative *CsTPS* genes belong to the TPS-a subfamily, from which, to our knowledge, just four proteins have been functionally characterized up to date. Conversely, numerous peptides with MonoTPS activity, almost covering every subclade from the phylogenetic tree (Figure 5) have been reported. Then, gene expression of seven putative *CsSesquiTPS* in different sweet orange tissues, such as peel, pulp, leaves, and flowers, was further examined.

For *Cs5g23510*, a unique sequence of 1,680 nt (*CsSesquiTPS1*, Acc. N° MF280919), encoding a protein of 559 amino acids (MW 64.54 kDa; pI 5.89) was obtained. This protein was 99.82% identical to that predicted in the CAP database, with just one amino acid change (M479I). Transcripts corresponding to this gene were observed in all tissues analyzed decreasing their accumulation in pulp as fruit matured (Figure 6B).

For *Cs4g12120*, a unique sequence of 1,689 nt was obtained (*CsSesquiTPS2*, Acc. N° MF280920). It encoded a 562 amino acid protein (MW 65.09 kDa; pI 5.79) identical to that annotated in the CAP database as Cs4g12120.2. Accumulation of this mRNA increased in orange peel along maturation, while in pulp it was only detected at the immature green stage. Expression of this gene was not detected in leaves at any developmental stage analyzed, while in flowers besides the mRNAs observed in fruit tissues, an additional transcript of about 1,400 nucleotides longer was also detected. The shorter predicted mRNA (Cs4g12120.1, 1,551 nt) was not detected at any of the tissues analyzed (Figure 6C).

Orange1.1t03302 cDNA clones corresponded to a unique ORF of 1,683 nt (*CsSesquiTPS3*, Acc N° MF280921) for a predicted protein of 560 amino acids (MW 64.25; pI 5.43), 74 more than that predicted in the CAP database, and contained all characteristic TPS motifs (Supplementary Figure 2A). This longer transcript was detected in the peel of orange fruit until degreening and in pulp of immature green fruits. In flowers and young leaves, mRNAs of the same size were detected, while in adult leaves only a much longer transcript (about 5,000 nt) was distinguished (Figure 6D).

For *Cs4g12350*, a 1,668 nt unique sequence (*CsSesquiTPS4*, Acc. N° MF280922), coding for a polypeptide of 555 amino acids (MW 64.37; pI 5.32) identical to that predicted in the CAP database, was obtained. This transcript was observed in peel, independently of fruit developmental/maturation stage, and in the pulp of immature green fruits. It was also detected in flowers and in all leaf tissues analyzed (Figure 6E). Shorter mRNAs were observed in peel from full colored fruits and in pulp from mature green and breaker fruits.

For *Cs4g12400* gene, two cDNA clones of 1,668 nt (Acc. N° MF280923 and MF280924 for clones *CsSesquiTPS5a* and *b*, respectively) were obtained, both coding proteins of 555 amino acids, highly identical between them (95.86%) and to that predicted by the CAP database (96.04–99.82% for clones a and b, respectively). Predicted protein (Cs4g12400) and CsSesquiTPS5a had only one difference, a conservative amino acid substitution at the N-terminus (K15Q, Supplementary Figure 2B). On the other hand, CsSesquiTPS5b presented 22 amino acid substitutions (13 of them conservative, Supplementary Figure 2B) when compared with CsSesquiTPS5a. Transcripts corresponding to Cs4g12400 were detected in all tissues analyzed (Figure 6F).

Three different cDNA clones were retrieved for *orange1.1t004360*, two of them (*CsSesquiTPS6a* Acc. N° MF280925, and *CsSesquiTPS6b* Acc. N° MF280926) encoding proteins identical to those predicted from transcripts orange1.1t004360.1, and orange1.1t004360.2, with 574, and 567 amino acids, respectively. The third isolated sequence (*CsSesquiTPS6c*, Acc. N° MF280927) coded for a polypeptide 99.6% identical to orange1.1t004360.2, with two non-conservative substitutions (Asp for Gly) at positions 105, and 319. Transcription of this gene was detected in flower, leaves, and fruit tissues at certain developmental stages (Figure 6G).

All cDNA clones corresponding to *Cs4g11980* showed an equal sequence of 1,701 nt (*CsSesquiTPS7*, Acc. N° MF280928), 99.29% identical to the ORF predicted in the CAP database. From the 12 nucleotide changes detected between both sequences, just six amino acid variations were found among them (Supplementary Figure 2C), showing 98.92% identity at amino acid level. Expression of this gene was detected in peel and pulp of immature green fruits, in leaves and flowers (Figure 6H).

### Functional characterization of the putative SesquiTPSs in *E. coli*

**Figure 7:**
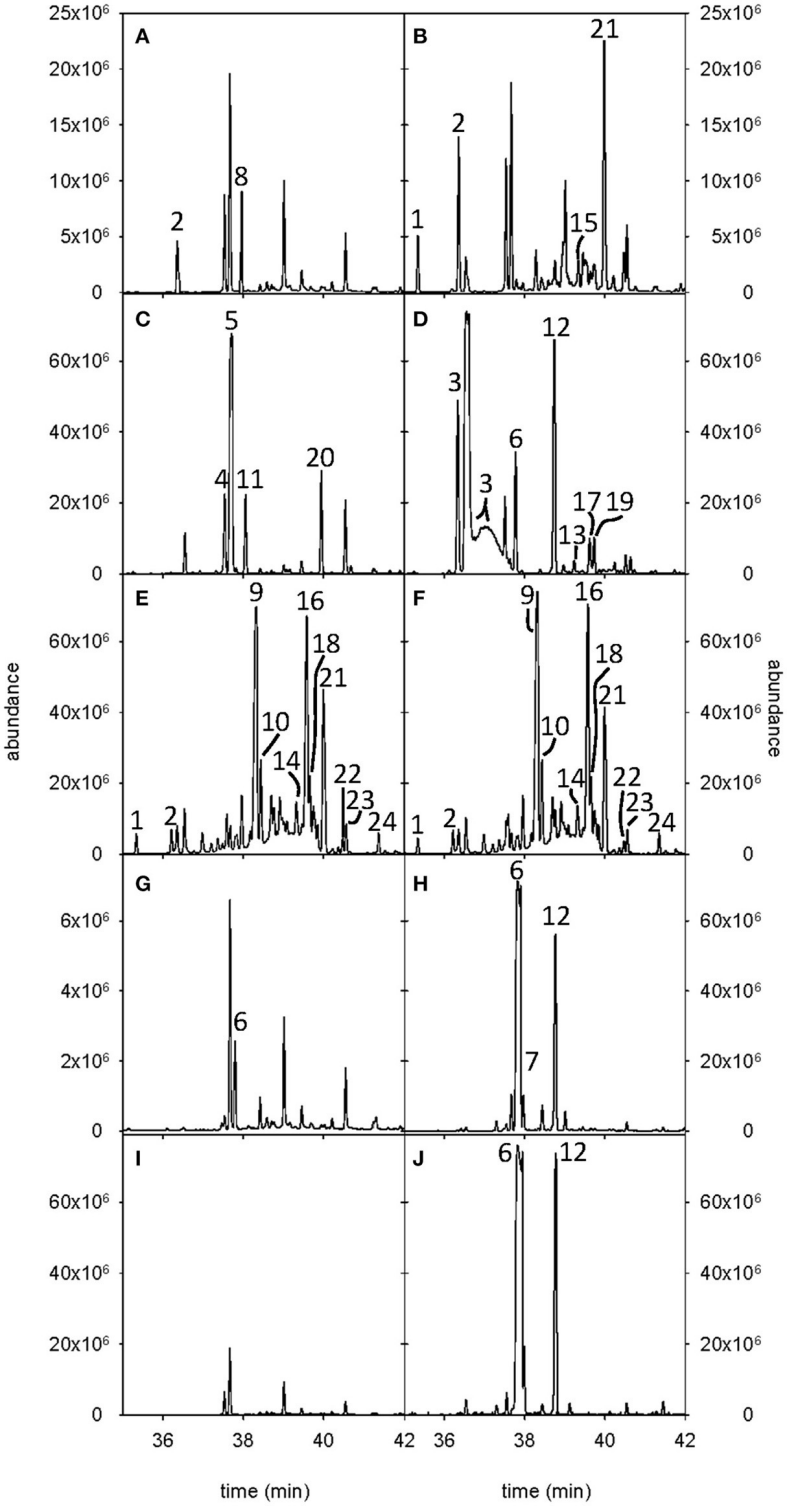
Total ion chromatograms of the products of the recombinant proteins using FPP as substrate. Analysed CsSesquiTPSs were **(A)** CsSesquiTPS1, **(B)** CsSesquiTPS2, **(C)** CsSesquiTPS3, **(D)** CsSesquiTPS4, **(E)** CsSesquiTPS5b, **(F)** CsSesquiTPS5a, **(G)** CsSesquiTPS6a, **(H)** CsSesquiTPS6b, **(I)** CsSesquiTPS6c, and **(J)** CsSesquiTPS7. The reaction products were identified by comparison of their GC-MS profiles with NIST libraries and authentic standards when available. Key to the sesquiterpene products were: 1, α-cubebene; 2, α-copaene; 3, β-elemene; 4, zingiberene; 5, *Z*-β-farnesene; 6, β-caryophyllene; 7, 10,10-dimethyl-2,6-dimethylene bicycle (7,2,0) undecane; 8, *Z*-β-cubebene; 9, allo-aromadendrene; 10, γ-gurjunene; 11, β-maaliene; 12 α-humulene; 13, γ-selinene; 14, (±) cadinene; 15, γ-cadinene; 16, ledene; 17, β-selinene; 18, longifolene; 19, α-selinene; 20, β-sesquiphellandrene; 21 β-cadinene; 22, 1,4-cadinadiene; 23, α-muurolene; 24, 4,11-selinadiene. Those unnumbered peaks correspond to terpene products synthetized by *Escherichia coli* endogenus enzymes or to PDMS fiber-residues.

Functional characterization was carried out by expression of recombinant proteins in *E. coli*, and *in vitro* enzyme assays using FPP as substrate. Analysis by SDS-PAGE showed that sufficient soluble protein was formed for all constructs (data not shown). Volatile terpenes produced by the different recombinant proteins were analyzed by GC-MS. Analysis of the sesquiterpene products generated by CsSesquiTPS1 showed the presence of a 62:38% mixture of (*Z*)-β-cubebene and α-copaene (Figure 7A). The major sesquiterpene product of CsSesquiTPS2 was tentatively identified as β-cadinene (59.19 ± 4.63% of total), followed by α-copaene (25.84 ± 2.76% of total) along with two minor products, α-cubebene and γ-cadinene (9.96 ± 1.11 and 4.98 ± 1.76% of total, respectively, Figure 7B). Enzyme coded by *CsSesquiTPS3* converted FPP to β-farnesene as the major product (74.74 ± 1.94% of total) with lesser amounts of β-sesquiphellandrene, zingiberene, and β-maaliene (16.07 ± 1.36, 4.78 ± 1.40, and 3.91 ± 1.10% of total, respectively, Figure 7C). More than half of CsSesquiTPS4 sesquiterpene production corresponded to β-elemene. In addition, between 6.22 and 12.68% of β-caryophyllene (6.22 ± 0.50%), α-humulene (12.68 ± 0.60%), and α-, β- and γ-selinene (9.82 ± 8.16, 9.84 ± 7.92, and 7.81 ± 4.70%, respectively) were detected (Figure 7D). Both *CsSesquiTPS5* clones (a and b) catalyzed the formation of multiple products from FPP, being all them bicyclic, except traces of the tricyclic sesquiterpene longifolene (Figures 7E,F). In both cases, the three major products (at least 20% each) were allo-aromadendrene (23.55 ± 12.43 and 25.67 ± 10.05% of total, for clones a and b, respectively), ledene (23.88 ± 0.80 and 20.43 ± 5.60% of total, for clones a and b, respectively) and β-cadinene (25.52 ± 6.79 and 31.58 ± 16.22% of total, for clones a and b, respectively). Trace to small amounts of α-cubebene, α-copaene, γ-gurjunene, (+/−)-cadinene, longifolene, α-muurolene, and 4,15-selinadiene were also found. In the case of *CsSesquiTPS6* clones, each showed different activities when FPP was used as substrate (Figures 7G–I). CsSesquiTPS6a produced exclusively β-caryophyllene, while CsSesquiTPS6b rendered higher amounts of β-caryophyllene (73.51 ± 0.26% of total) together with α-humulene (23.14 ± 0.30% of total). Clone CsSesquiTPS6c did not produce any sesquiterpene. As CsSesquiTPS6b, CsSesquiTPS7 produced β-caryophyllene and α-humulene, and in similar proportions (about 75:25%, respectively, Figure 7J).

### Protein modeling

**Figure 8:**
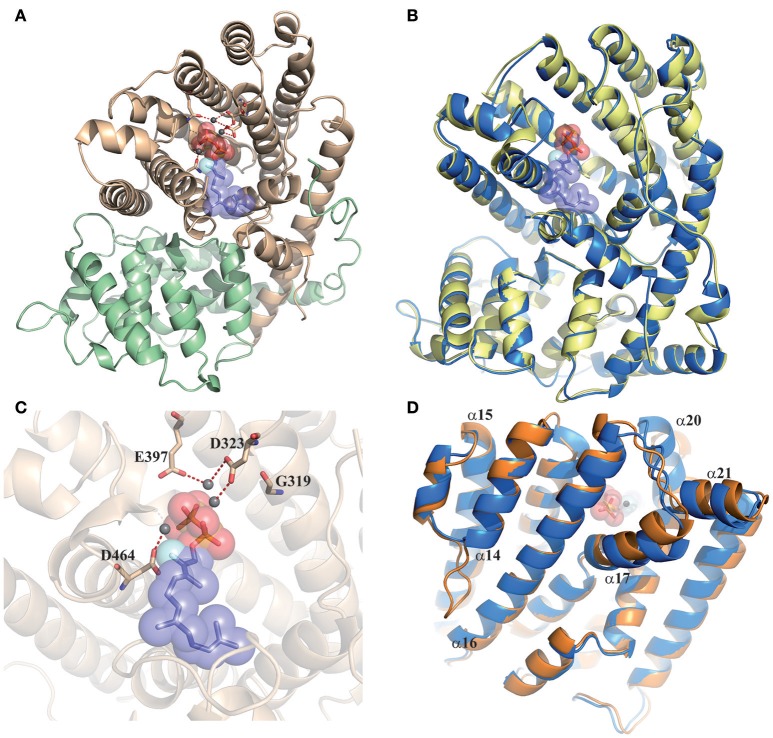
Tertiary structure models of sweet orange sesquiTPSs. **(A)** Modelled structure of CsSesquiTPS5a with FPP and a trio of Mg^2+^. The C-terminal domain is shown in pale brown, with three bound Mg^2+^ atoms (shown as dark gray spheres) coordinated by D309, D313, Q387, and N453. The N-terminal domain is displayed in pale green. Red-dotted lines represent polar interaction between Mg^2+^ atoms and D309, D313, Q387, and N453 residues. **(B)** Comparison of CsSesquiTPS7 (yellow) and CsSesquiTPS6b (blue) predicted tertiary structures. **(C)** Detail of CsSesquiTPS6c catalytic cleft in which D323, D464, E397 interaction with Mg^2+^ atoms is predicted, while G303 is not able to interact. **(D)** Comparison of CsSesquiTPS6a (orange) and CsSesquiTPS6b (blue) predicted tertiary structures.

The different proteins encoded by *CsSesquiTPS5, CsSesquiTPS6* and *CsSesquiTPS7* were 3D modeled based on the crystal structure of the δ-cadinene synthase from cotton (Gennadios et al., 2009, Protein Data Bank code 3G4D_(A). All predicted structural models show high quality (coverage >94%, sequence similarity >0.44, high GMQE and QMEAN scores), and fitted well with the crystallographic model, displaying the two TPS characteristic domains: the functional C-terminal domain consisting of 14 α-helices forming a central cavity and the amino terminal domain formed by two 10 antiparallel α-helices (Figure 8). The aspartate-rich motifs DDXXD (located on Helix α-13) and NSE/DTE motif (located helix α-20) both involved in binding a trio of divalent magnesium ions, co-orientated to the catalytic pocket.

Most of the non-equivalent amino acid substitutions identified between CsSesquiTPS5a and b were located in α-helices without causing significant alterations of the predicted structure (i.e., E134A, L243H, Y369C). Some others, such as S129P and R191C, were found on the outer surface of the protein, in contact with the solvent, and neither expected to induce alterations in its conformation. The change Q458H was found near to the active site. The light steric effect that this substitution could have may be somehow compensated by the Q482A change, which in the tertiary structure faces with the previous one, thus not altering size or conformation of the catalytic cavity. In the cleft, D309, and 313, N453, and Q387 were found to interact with the Mg^2+^ cluster. A representative model of CsSesquiTPS5 proteins is shown in Figure 8A.

Despite their differences at sequence level, CsSesquiTPS7, and CsSesquiTPS6b both produced β-caryophyllene and α-humulene, and in similar proportions (Figure 7). By modeling and comparing their structures, we found that most amino acid changes between both proteins were solvent exposed and were not expected to induce important changes in the protein structure (Figure 8B). Just A256E and I260R (counted on CsSesquiTPS6b sequence), both located on an α-helix, were found to disturb somewhat the facing N-terminal loop, in which E41D, and T44P substitutions could compensate the structural differences. Consequently, the predicted N-terminal end varies slightly between both proteins, but the predicted catalytic pocket remained almost identical. Contrary, on CsSesquiTPS6c, the D319G change prevents the interaction of this residue with one of the Mg^2+^ atoms (Figure 8C). On the other hand, in comparison to CsSesquiTPS6b, insertion of seven amino acids in the loop between α-helix 14 and 15 of CsSesquiTPS6a would allow establishment of new hydrophobic and hydrophilic interactions with faced helixes α-15 and α-16, the last harboring the E404 residue involved in interactions with the Mg^2+^ cluster (Figure 8D). Besides, the loop may also stabilize the α-helix 14 itself, providing extra rigidity to the preceding α-helix-13, which includes the DDXXD signature. Altogether, these effects may result in higher rigidity and stability of the C-terminal domain of the structure. Modelling also predicts small deviations of helixes α-17, α-20, and α-21, the second one including D471, also involved in binding the bivalent metal ion cofactor.

## Discussion

*Citrus sinensis* genome mining revealed 95 loci with high homology to known TPS from other plant species (Supplementary Table 3). In other angiosperm plants in which TPS family has been also characterized at a genomic level the number of identified *TPS* genes is between 42 and 76% lower to that of orange (Falara et al., 2011; Nieuwenhuizen et al., 2013; Liu et al., 2014). It has been proposed that the larger *TPS* gene families are found in species with specialized structures for storing volatile terpenes (Külheim et al., 2015), such as *Citrus*, which accumulate volatiles in oil glands. Therefore, *CsTPS* is the third largest family described in angiosperms species up to date, after that of *Eucalyptus* (Külheim et al., 2015) and grapevine (Martin et al., 2010).

As observed in other genomes (Aubourg et al., 2002; Martin et al., 2010; Falara et al., 2011; Nieuwenhuizen et al., 2013; Liu et al., 2014; Külheim et al., 2015) *CsTPSs* were organized in clusters (Figure 2), some of them formed by a functional gene and a pseudogene originated by partial duplication (or alternatively complete duplication and posterior partial deletion) of the former (Figure 2, i.e., *Cs5g22980*/*Cs5g22990, Cs7g18530*/*Cs7g18500*). For pairs of full length *TPSs* located in similar regions of the genome, phylogenetic analysis envisaged a common origin (Figure 5). Such are the cases, for example, of *Cs4g12480*/*Cs4g11320, Cs2g23470*/*Cs2g24110, Cs3g04170*/*Cs3g04360, Cs4g12090*/*Cs4g12120, Cs4g12050*/*Cs4g12060*/*Cs4g12080, Cs5g12880*/*Cs5g12900*, or *Cs5g23540*/*Cs5g23510*. Comparison of their genomic structure (Figure 3) and their high identity at the amino acid level (>94%, Supplementary Table 4) further supports their paralog nature. In a few cases, putative paralogs were found in different chromosomes, for example *Cs3g07920*/*Cs7g18530* (91.91% identity) or *Cs3g07880*/*Cs7g1764*0 (92.98% identity). *TPS* genes duplications can also be traced in other species (Aubourg et al., 2002; Martin et al., 2004, 2010; Falara et al., 2011; Nieuwenhuizen et al., 2013; Külheim et al., 2015), and this phenomena, followed by mutations and neofunctionalization, has been pointed out as the primary mechanism leading to plant TPS evolution and diversification (Trapp and Croteau, 2001). Besides, the hybrid nature of sweet orange [(*C.maxima* × *C. reticulata*) × *C. reticulate*], implies that diversification of gene families can also occur as a result of the combination of alleles from ancestral parents, as shown to happen for some nuclear genes studied (Garcia-Lor et al., 2013). In addition, ancestral parents can be distinguished based on their volatile profiles in different tissues (Yamamoto et al., 2013), thus advising that terpene metabolism is different between both species, and that sweet orange may have inherited TPS characters from both. As the genome analyzed in this work derives from a double-haploid *C. sinensis* callus line, allele's analysis was precluded. Further work with recently released genomes of pummelo, *Citrus ichangensis* Swing., citron and Chinese box orange (*Atalantia buxifolia* (Poir.) Oliv.) (Wang et al., 2017) joined to volatilomic analysis are required to shed light on the allelic variation present in the citrus *TPS* family as well as to infer the probable origin of orthologous genes.

The number of putative full length genes identified in the sweet orange genome (55, Supplementary Table 5) was within the range of putative functional genes identified in other angiosperms. It was similar to that identified in grapevine, approximately half to that identified in *Eucalyptus* species, about twice as much as *Arabidopsis*, soybean, and tomato and 5.5 times higher than that identified in apple (Aubourg et al., 2002; Martin et al., 2010; Falara et al., 2011; Nieuwenhuizen et al., 2013; Liu et al., 2014; Külheim et al., 2015). Following Trapp and Croteau (2001) criteria, who classified *TPS* genes according to intron/exon pattern, four class I putative full length *CsTPSs* were found in the sweet orange genome. Class I genes are involved in primary metabolism, and the rest of *TPS* genes involved in secondary metabolism are descendant of them by exon loose and diversification. Class I TPS family usually includes TPS-c subfamily, formed by copalyl diphosphate synthases, and considered the most ancestral TPS family, and TPS-e/f subfamily, corresponding to ent-kaurene, and geranyl linalool synthases. Consequently, phylogeny analysis located on the root two genes (*Cs5g31210, Cs5g15530*) with high homology to copalyl diphosphate synthases (Supplementary Table 3, Figure 5). Consistent with this function, predicted proteins lacked DDXXD signature, required for cleaving prenyl diphosphate unit, but have the DXDD motif necessary to cyclize GGPP to ent-copalyl diphosphate (Prisic et al., 2007). Putative ent-kaurene synthase (orange1.1t03278) and geranyl linalool synthase (Cs2g06470) were also identified based on their homology and phylogeny. The genomic structure, the larger size of predicted peptides and the analysis of conserved motifs support this classification (Figure 3, Supplementary Figure 1). For example, all four sequences have at the N-terminus a γ-diterpene synthases domain highly conserved among TPS-c, -e and -f groups (Trapp and Croteau, 2001; Cao et al., 2010). The number of *TPS-c* and *-e/f* genes described in fully annotated plant genomes is also small (1–14), with a maximum of three (*Oryza sativa* TPS-c) or 11 (*Eucalyptus globulus* TPS-e/f) members, indicating that in general, these genes did not experienced a high rate of duplication (Aubourg et al., 2002; Martin et al., 2010; Chen et al., 2011; Falara et al., 2011; Nieuwenhuizen et al., 2013; Liu et al., 2014; Külheim et al., 2015).

More than half of the remaining putative full length Class III Cs*TPSs* presented the 6 intron/7 exon architecture characteristic of mono- and SesquiTPSs (Trapp and Croteau, 2001; Aubourg et al., 2002; Martin et al., 2004, 2010) while in others, partial deletions/insertions conducing to alteration of this structure have occurred (Figure 3, Supplementary Table 5). Conservation was also found at exon length, intron phase and exon/intron boundaries (Figure 4). Just 37 of these genes coded for peptides harboring all the reported characteristic TPS motifs RRX8W, RDR, DDXXD, and NSE/DTE, which in addition, presented a conserved distribution along exons (Figure 3), as described previously (Trapp and Croteau, 2001). As expected, all putative full length class III CsTPSs claded together and subdivided in three subfamilies (Figure 5) TPS-a1, TPS-b, and TPS-g, which are angiosperm-specific (Chen et al., 2011).

Most members of CsTPS-b family sheltered a presumptive transit peptide and all the characteristic motifs of MonoTPS. Previously, functionally characterized citrus MonoTPSs also claded in this group. β-pinene (AAM53945 and BAD27260), γ-terpinene (BAD27258 and AAM53943), and D-limonene synthases (BAD27257, AAM53944, AAM53946, BAD27256, and BAF73932) from different *Citrus* types were closely clustered, suggesting that their evolution to different enzymatic activities occurred before *Citrus* speciation. A second cluster of putative D-limonene synthases was closely related to geraniol synthases (BAM29049 and possibly orange1.1t04366) than to other D-limonene synthases, suggesting that duplication of D-limonene synthase coding genes occurred before the divergence of *Citrus* species. Most of the variation found between MonoTPS from different *Citrus* types is located at the N-terminus, which is not directly involved in the determination of the product specificity, while they present high levels of identity between the TPS conserved signatures. However, the enzymatic activity of each protein cannot be accurately predicted, and should be confirmed experimentally, as single amino acid substitutions can have profound effects on catalytic activity. CsTPSg subfamily, closely related to CsTPS-b, was formed by predicted proteins lacking RRX8W motif (Supplementary Table 5), which is a common feature of this subfamily of acyclic TPSs (Chen et al., 2011). Consistently, the previously characterized linalool synthases from *C. unshiu* (Shimada et al., 2014) cladded in this group. In basis of the phylogenetic relationship and sequence identity, putative D-limonene and linalool synthases were the most abundant duplicated full length proteins found within the sweet orange genome. This is not surprising as D-limonene and linalool are among the most abundant volatiles emitted by sweet orange tissues (Supplementary Table 1). Different citrus genotypes, including oranges, mandarins, lemons, citrons, and grapefruits, also emit high levels of D-limonene and linalool from flowers (Azam et al., 2013b) and leaves (Azam et al., 2013a). Additionally, both compounds have been reported as involved in citrus resistance to different pest and pathogens (Ben-Yehoshua et al., 2008; Rodríguez et al., 2011, 2014, 2015; Shimada et al., 2014; Rodrigues Marques et al., 2015). The existence of different genes with a common origin encoding redundant proteins could indicate that diversification, in addition to sequence level, has occurred at the level of gene expression patterns. Results from RNA-seq data point to this direction, as different expression profiles were obtained for putative sweet orange D-limonene synthases from different clusters (*Cs3g04170*/*Cs3g04360* vs. *orange1.1t04366*; Figure 5). Similarly, linalool synthases cladding in different groups (*Cs2g07250* vs. *Cs2g07240*/*orange1.1t02448*/*orange1.1t03307*) showed different expression patterns (Figure 5). Although more detailed expression analysis are required to confirm expression differences between duplicated genes previous data reinforces this hypothesis. For example, D-limonene synthase *CitMSE2* from rough lemon was expressed specifically in peel fruit, while *CitMSE1* expression was lower in the peel and it was also detected in flowers, and juice sacs (Shimada et al., 2005). None of them was expressed in leaf or stem, so it may be assumed that further chloroplastic D-limonene synthases are still pendant of characterization. In the case of linalool synthases genes from Satsuma mandarin, the expression of one of them (corresponding to BAP75559) was induced in response to *Penicillium italicum* and *Xanthomonas citri* inoculation in leaves and peel of mature fruits, while the other (corresponding to BAP75560) was expressed at a much lower level, and induced only in leaves challenged with the bacterium (Shimada et al., 2014).

Despite the low percentage of sesquiterpenes emitted by sweet orange (Figure 1, Supplementary Table 1), the TPS-a subfamily, typically formed by SesquiTPSs (Trapp and Croteau, 2001; Chen et al., 2011), encompassed the largest number of putative full length *CsTPSs*. In other angiosperm plants in which the *TPS* family has been analyzed at genomic level, such as rice, grape, *Arabidopsis*, apple, eucalyptus, and tomato, TPS-a is also the largest subfamily (Aubourg et al., 2002; Martin et al., 2010; Chen et al., 2011; Falara et al., 2011; Nieuwenhuizen et al., 2013). The only exception up to date is soybean, in which TPS-g subfamily is the largest one (Liu et al., 2014). The high number of plant SesquiTPSs could be related to their possible role in defense. For example, feeding by larvae of the beetle *Diabrotica virgifera virgifera* to maize roots induced β-caryophyllene release to attract predatory entomopathogenic nematodes (Degenhardt et al., 2009) while antifungal sesquiterpene derivates are produced in response to fungal infection in maize (Huffaker et al., 2011). Rice and sorghum emit a complex mix of sesquiterpenes involved in indirect defense upon caterpillars damage (Yuan et al., 2008; Zhuang et al., 2012). Production of a sesquiterpene identified from a wild tomato cultivar provides resistance to different herbivores (Bleeker et al., 2012), while in *Arabidopsis* β-caryophyllene protects flowers from microbial infection (Huang et al., 2012). In citrus, studies regarding volatiles and pest/pathogen interactions are still scarce, but existing evidences indicate that they may play an important role in such interplays. Linalool/nerolidol synthase gene expression was induced in mandarin leaves by wounding and *Xhantomonas citri* infection (Shimada et al., 2014). Some mono and sesquiterpene compounds have been proposed to be important in the relation of citrus hosts with *D. citri*, vector of *Candidatus* Liberibacters (Patt and Sétamou, 2010; Coutinho-Abreu et al., 2014; Lin et al., 2016; dos Santos Andrade et al., 2016; Alquézar et al., 2017). However, in spite of their importance, just five citrus SesquiTPSs have been characterized up to date (Maruyama et al., 2001; Sharon-Asa et al., 2003; Shimada et al., 2012, 2014; Uji et al., 2015). The valencene synthase cloned from Valencia sweet orange coded for a protein virtually identical (99.64%) to Cs5g12900.2, being the only two amino acid changes located at the N-terminus. Probably, these two versions of the same gene in the same genotype correspond to alleles of the same gene, each coming from an ancestral parent. Most of the amino acid changes between Satsuma mandarin linalool/nerolidol synthase and Cs4g12110.1, which clustered together, were also found at the non-catalytic N-terminus, with just one substitution for an amino acid with similar accessible surface area in the C-terminal position (M351I counted on BAP75561 sequence). As nerolidol is accumulated in different Valencia sweet orange tissues (Njoroge et al., 2005; dos Santos Andrade et al., 2016), it can be assumed that Cs4g12110.1 encodes a functional nerolidol/linalool synthase despite the change in hydrophobicity of the single altered amino acid found in the C-terminus. Although germacrene-A, δ-elemene and β-farnesene synthases had been characterized from other *Citrus* types (Maruyama et al., 2001; Shimada et al., 2012; Uji et al., 2015), phylogenetic and homology analysis did not allow a reliable prediction of any orange putative SesquiTPS with these activities. Especially intriguing is the case of β-farnesene synthase, since the corresponding product was found between the components of the aromatic profile of orange leaves and flowers (Figure 1), while predicted CsTPS phylogenetically related to *Citrus junos* β-farnesene synthase lacked the RDR signature (Supplementary Table 5) essential for catalysis. Then, we attempted to isolate transcripts corresponding to *Cs4g11320* and *orange1.1t03302*, but we just cloned one type of transcripts, longer than predicted and containing essential motifs for TPS activity (Supplementary Figure 2A). Cloned *CsSesquiTPS3* coded for a functional β-farnesene synthase (Figure 7C) and was found to be expressed in fruits, leaves and flowers (Figure 6D). Besides, a (*Z*)-β-cubebene/α-copaene synthase, two β-caryophyllene synthases and three multiproduct enzymes yielding β-cadinene/α-copaene, β-elemene, and β-cadinene/ledene/allo-aromandendrene as major products were identified (Figure 7). Overall, about 50% of sesquiterpenes identified in orange emission profile were produced by the CsSesquiTPSs characterized in this work. However, transcripts accumulation, and correspondent sesquiterpene emission did not directly correlate in all cases. For example, transcripts from β-farnesene synthase were not found in mature orange leaves emitting this sesquiterpene (Figures 1, 6D). This apparent discrepancy probably arises from the fact that the emitted volatiles do not always coincide with the volatile compounds accumulated in a given plant/tissue, as it has been shown in some plants (Llusia and Peñuelas, 2000; Ormen et al., 2011). Even more, it can also occur that sesquiterpene synthase genes expression profile and accumulation of the corresponding product in plant tissues do not correlate, as it has been reported for germacrene-A synthase in lavender (Benabdelkader et al., 2015). Concretely, citrus terpenes are synthetized and stored in specialized oil glands, and although there are no works reporting comparative volatile emission/content, our unpublished results point that they can greatly differ in some tissues/developmental stages (i.e., while D-limonene contributed <3% to total volatiles emitted from peel of mature fruit it constitutes more than 90% of total volatiles accumulated in this tissue).

All but one of the CsSesquiTPSs characterized in this work could synthetize more than one compound from FPP (Figure 7). This is not unusual, and several multiproduct SesquiTPSs have been reported before (Lücker et al., 2004; Göpfert et al., 2009; Crocoll et al., 2010; Falara et al., 2011; Keeling et al., 2011; Nieuwenhuizen et al., 2013; Yang et al., 2013; Külheim et al., 2015; Yahyaa et al., 2015), some of them catalyzing the synthesis of more than 50 different sesquiterpenes (i.e., γ-humulene synthase from grand fir, Steele et al., 1998). Such ability has been associated to the SesquiTPS electrophilic reaction mechanism, which can have evolved to maximize the number of products while using the minimum genetic, and enzymatic resources (Steele et al., 1998). The highest number of sesquiterpene products was obtained from CsSesquiTPS5, for which two different transcripts coding for two different isoforms were isolated. Both predicted proteins, despite differing in some amino acids, rendered equivalent quaternary structures when modeled (Figure 8) and, in good agreement, same products profile when assayed in *E. coli* (Figures 7E,F). Similar results were obtained for CsSesquiTPS7 and CsSesquiTPS6b: different peptides with similar quaternary structure (Figure 8B) and same catalytic activity (Figures 7G,H). Alternative transcript *CsSesquiTPS6c* coded for a protein lacking first aspartate of metal binding signature (DDXXD), being then unable to establish the interaction with Mg^2+^ triplet (Figure 8C), essential for the initial ionization of FPP, and consequently inactive (Figure 7I). CsSesquiTPS6a produced *in vitro* about one order of magnitude less β-caryophyllene than CsSesquiTPS6b and no α-humulene (Figure 7G). CsSesquiTPS6a *in vitro* inferior catalytic activity should be related to a sequence of seven amino acids present in this protein and absent in CsSesquiTPS6b, as this is the only variation between both peptides. CsSesquiTPS6a modeling showed that these additional residues formed a longer loop between helixes α-14 and α-15 that may confer higher rigidity to some other α-helixes, even from the catalytic pocket (i.e., helix α-13, 16, and 20, Figure 8D). These conformational changes affecting position of essential motifs for SesquiTPS catalytic activity can explain the lower enzymatic capacity of CsSesquiTPS6a. However, to decipher relative individual importance of these conformational changes further study is required.

In conclusion, the CsTPS family has been characterized for the first time at a genomic level. Before this work, the only genomic approach reported to study *Citrus* TPS family, based on expressed sequence tags, had identified 6 and 9 sweet orange mono and SesquiTPSs, respectively (Dornelas and Mazzafera, 2007). Present work reveals that the TPS family is larger than predicted before, with 28, 18, 2, 2, and five putative functional TPS-a, TPS-b, TPS-c, TPS-e/f, and TPS-g proteins. These results could allow faster identification and characterization of remaining uncharacterized CsTPSs. Besides, 7 CsSesquiTPSs have been characterized, enriching our knowledge about the CsSesquiTPS family, which was poorly characterized up to date. Gaining knowledge about the enzymes responsible for the biosynthesis of terpenes in citrus, as well as to expand our understanding about the role of these compounds in defense, could allow the development of biotechnological strategies for the control of pests and diseases. We have recently shown that β-caryophyllene emitted by either a transgenic *Arabidopsis* line or a chemical dispenser repels *Diaphorina citri* (Alquézar et al., 2017). Then, the use of β-caryophyllene synthase genes identified in this work could allow the generation of cisgenic citrus cultivars potentially less attractive to this psyllid insect, vector of *Candidatus* Liberibacter species causing HLB, nowadays the most destructive disease of citrus worldwide (McCollum and Baldwin, 2016).

## Author contributions

BA performed genome analysis, sesquiterpene synthase isolation, expression analysis and functional characterization and drafted the manuscript; AR performed VOC analyses; MP performed protein molecular modeling and interpreted the results; LP conceived, designed, and supervised the study. All authors reviewed and approved the final version of the MS.

### Conflict of interest statement

The authors declare that the research was conducted in the absence of any commercial or financial relationships that could be construed as a potential conflict of interest.

## Footnotes

^1^http://faostat3.fao.org/home/E

^2^http://citrus.hzau.edu.cn/orange/

^3^http://www.ncbi.nlm.nih.gov/orffinder/

^4^http://phytozome.jgi.doe.gov/pz/portal.html#!info?alias=Org_Csinensis

^5^http://phytozome.jgi.doe.gov/pz/portal.html#!info?alias=Org_Cclementina

^6^http://pfam.xfam.org/

^7^http://weblogo.berkeley.edu.

^8^http://web.expasy.org/compute_pi/

^9^http://www.cbs.dtu.dk/services/ChloroP/

^10^http://www.cbs.dtu.dk/services/TargetP/

^11^http://biit.cs.ut.ee/clustvis/

^12^http://www.pymol.org/

^13^http://www.ncbi.nlm.nih.gov/cdd/

## Supplementary material

Supplementary Table 1 Primers used for the amplification of full length cDNAs (plasmid PJET1.2/blunt), expression and subcloning for functional analysis [plasmid pET-45b(+)] of *CsTPS* genes. Start and stop codons of the predicted proteins are underlined. Nucleotides complementary to destination vector are in italics and restriction sites used for cloning purposes are bold-lettered. Nucleotides introduced in primer sequences in order to maintain the ORF of the expression vector are lowercased. O, primer orientation. RE, restriction enzymes used for cloning.

Supplementary Table 2 Percentage emission of volatiles in different Pineapple sweet orange tissues. Data represent mean values ± SE and are derived from at least two samples replicates.

Supplementary Table 3 Tabulated information of the 95 putative *TPS* gene models identified in the sweet orange genome. The content of each column is as follows: Name: CAP-database nomenclature for the 95 putative terpene synthase (*TPS*) gene models. Genes *Cs5g23510, Cs4g12120, orange1.1t03302, Cs4g12350, Cs4g12400, orange1.1t04360*, and *Cs4g11980*, corresponding respectively to *CsSesquiTPS 1*–*7* cloned in this work, are underlined. Length: Predicted nucleotide number of the gene. Start: First position of the *TPS* coding sequence in the chromosome. Stop: Last position of the *TPS* coding sequence in the chromosome. Strand: (+) forward strand and (−) reverse strand relative to the chromosome. Chrom: Predicted chromosome in which the *TPS* gene model is located. Intron: number of annotated intron for *TPS* gene model. Exons: number of annotated exons for *TPS* gene model. Alternative transcripts: Number of predicted alternative transcripts for *TPS* gene model. CDS length: Predicted nucleotide number of coding sequence. Prot length: Predicted amino acid number of the *TPS* open reading frame. Homology analysis: Comparison, by using BLASTp algorithm, of predicted TPS protein with all the protein sequences in non-redundant protein sequences database (nr) from NCBI. Best hit: Accession number of the best matched database sequence. Best non-*Citrus* related match was selected. Max score: the highest alignment score from that database sequence. Total score: the total alignment scores from all alignment segments. Query coverage: the percentage of query covered by alignment to that database sequence. *E*-value: the best Expect value of all alignments from that database sequence. Ident: the highest percent identity of all query-subject alignments. Description: the description/title of matched database sequence. Identified motifs: predicted domains detected in pFAM^5^ and CCD^11^ resources.

Supplementary Table 4 Matrix of overall percent identity of amino acid predicted sequences between putative CsTPSs identified from the CAP database and with previously characterized CsTPSs. Genes *Cs5g23510, Cs4g12120, orange1.1t03302, Cs4g12350, Cs4g12400, orange1.1t04360*, and *Cs4g11980*, corresponding respectively to *CsSesquiTPS 1*–*7* cloned in this work, are underlined. Q94JS8, (E)-β-farnesene synthase from *Citrus junos*; AAQ04608, valencene synthase from *Citrus sinensis*; BAP75561, linalool synthase from *Citrus unshiu*; BAP75559, and BAP75560, linalool synthase from *Citrus unshiu*; BAF73932, and BAF73932, D-limonene synthase from *Citrus jambhiri*; BAD27256, and BAD27257, D-limonene synthase from *Citrus unshiu*; AOP12358, D-limonene synthase from *Citrus sinensis*; AAM53943, γ-terpinene synthase from *Citrus limon*; BAD27258, and BAD27259, γ-terpinene synthase from *Citrus unshiu*; AAM53944, and AAM53946, D-limonene synthase 1 from *Citrus limon*; AAM53945, β-pinene synthase from *Citrus limon*; BAM29049, geraniol synthase from *Citrus jambhiri*; BAD91046, (E)-β-ocimene synthase from *Citrus unshiu*; BAD91045, 1,8-cineole synthase from *Citrus unshiu*; BAF73933, β-pinene synthase from *Citrus jambhiri*, BAD27260, β-pinene synthase from *Citrus unshiu*; CuSTS-1, germacrene-A synthase from *Citrus unshiu*; BAP74389, δ-elemene synthase from *Citrus jambhiri*.

Supplementary Table 5 Tabulated information of the 55 putative class III terpene synthase proteins longer than 400 amino acids identified in the sweet orange genome. The content of each column is as follows: Name: CAP-database nomenclature for the 95 putative *TPS* gene models. Genes *Cs5g23510, Cs4g12120, orange1.1t03302, Cs4g12350, Cs4g12400, orange1.1t04360*, and *Cs4g11980*, corresponding respectively to *CsSesquiTPS 1*–*7* cloned in this work, are underlined. Intron: number of annotated intron for *TPS* gene model. Exon: number of annotated exons for *TPS* gene model. Class: TPS class (I-III) according to intron number. Family: classification of the TPS (TPS-a to TPS-h) according to phylogenetic analysis. Alternative transcripts: Number of predicted alternative transcripts for *TPS* gene model. CDS length: Predicted nucleotide number of coding sequence. Prot length: Predicted amino acid number of the *TPS* open reading frame. pI, theoretical isoelectric point; MW, predicted molecular weight, kDa; Plastidial import predictions: (I) Chloroplast import transit peptide prediction by Target P1.1 server, cTP meaning that chloroplast import signal has been detected with a predicted reliability value (R); (II) chloroplastic localization prediction according to ChloroP 1.1 server (II). Conserved motifs RDR, DDXXD and NSE/DTE. The amino acid sequence and their predicted position (E meaning exon) detected in each predicted protein is detailed. −, means that the motif was not localized.

Supplementary Figure 1 Alignment of the deduced protein sequences of putative full-length *Citrus* sinensis *TPS* genes with previously characterized *CsTPSs*. Q94JS8, (E)-β-farnesene synthase from *Citrus junos*; AAQ04608, valencene synthase from *Citrus sinensis*; BAP75561, linalool synthase from *Citrus unshiu*; BAP75559, and BAP75560, linalool synthase from *Citrus unshiu*; BAF73932, and BAF73932, D-limonene synthase from *Citrus jambhiri*; BAD27256, and BAD27257, D-limonene synthase from *Citrus unshiu*; AOP12358, D-limonene synthase from *Citrus sinensis*; AAM53943, γ-terpinene synthase from *Citrus limon*; BAD27258, and BAD27259, γ-terpinene synthase from *Citrus unshiu*; AAM53944, and AAM53946, D-limonene synthase 1 from *Citrus limon*; AAM53945, β-pinene synthase from *Citrus limon*; BAM29049, geraniol synthase from *Citrus jambhiri*; BAD91046, (E)-β-ocimene synthase from *Citrus unshiu*; BAD91045, 1,8-cineole synthase from *Citrus unshiu*; BAF73933, β-pinene synthase from *Citrus jambhiri*, BAD27260, β-pinene synthase from *Citrus unshiu*; CuSTS-1, germacrene-A synthase from *Citrus unshiu*; BAP74389, δ-elemene synthase from *Citrus jambhiri*. TPS conserved motifs are bold lettered.

Supplementary Figure 2 Comparison of amino acid sequences between predicted transcripts in the CAP database and those obtained in this work. **(A)**. Comparison of nucleotide sequences (lowercases) of *orange1.1t03302* predicted transcript in the CAP database **(A)** and those obtained in this work **(B)**. Identical nucleotides are white lettered on a black frame. Deduced amino acid for each triplet are detailed in capital letters. Characteristic *TPS* signatures are red-lettered. **(B)** Alignment of the deduced amino acid sequences of Cs4g12400 annotated in the CAP database (Cs4g12400) and from cDNAs cloned in this work (CsSesquiTPS5 **(A,B)**. Identical amino acids and conservative substitutions are shown on a black or gray background, respectively. **(C)** Alignment of the deduced amino acid sequences of Cs4g11980 annotated in the CAP database and from cDNAs cloned in this work (CsSesquiTPS7). Identical amino acids and conservative substitutions are shown on a black or gray background, respectively.
