# Supplementary material for: Genomic Analysis of Terpene Synthase Family and Functional Characterization of Seven Sesquiterpene Synthases from Citrus sinensis
Source: Front Plant Sci. 2017 Aug 24;8:1481. doi: 10.3389/fpls.2017.01481 (PMC5573811; doi:10.3389/fpls.2017.01481)
Supplement: Supplementary Table 1 — Primers used for the amplification of full length cDNAs (plasmid PJET1.2/blunt), expression and subcloning for functional analysis [plasmid pET-45b(+)] of CsTPS genes. Start and stop codons of the predicted proteins are underlined. Nucleotides complementary to destination vector are in italics and restriction sites used for cloning purposes are bold-lettered. Nucleotides introduced in primer sequences in order to maintain the ORF of the expression vector are lowercased. O, primer orientation. RE, restriction enzymes used for cloning. [file Table1.docx]

**Supplementary Table 1**. Primers used for the amplification of full length cDNAs (plasmid PJET1.2/blunt), expression and subcloning for functional analysis (plasmid pET-45b(+)) of *CsTPS* genes. Start and stop codons of the predicted proteins are underlined. Nucleotides complementary to destination vector are in italics and restriction sites used for cloning purposes are bold-lettered. Nucleotides introduced in primer sequences in order to maintain the ORF of the expression vector are lowercased. O, primer orientation. RE, restriction enzymes used for cloning.

| **Primer** | **Sequence (5´🡪 3´)** | **O** | **RE** | **Plasmid** | **Target gene** |
| --- | --- | --- | --- | --- | --- |
| B120 | ATGTCTTTAGAAGTTTCAGC | S |  | pJET1.2/blunt | *CsSesquiTPS1* (*Cs5g23510*) |
| B121 | TCATATCGGCACAGGATT | AS |  | pJET1.2/blunt |  |
| B122 | ATGTCTCTTCAAGTTTCAGC | S |  | pJET1.2/blunt | *CsSesquiTPS2* (*Cs4g12120*) |
| B123 | TCATATCGGCACAGGATTAA | AS |  | pJET1.2/blunt |  |
| B27 | AAAAATGTCCGCTCAAGTTC | S |  | pJET1.2/blunt | *CsSesquiTPS4* (*Cs4g12350*) |
| B211 | TCATATAGTGACAGGGTCTC | AS |  | pJET1.2/blunt |  |
| B25 | AACGGAATGAAAGATATGTC | S |  | pJET1.2/blunt | *CsSesquiTPS3* (*orange1.1t03302*) |
| B26 | CTTCAGATCGCAAGGGGTTC | AS |  | pJET1.2/blunt |  |
| B27 | AAAAATGTCCGCTCAAGTTC | S |  | pJET1.2/blunt | *CsSesquiTPS5*(*Cs4g12400*) |
| B28 | TCAGATGGTAACAGGGTCTC | AS |  | pJET1.2/blunt |  |
| B73 | GCATGAGGGATCTTAAGAG | S |  | pJET1.2/blunt | *CsSesquiTPS6* (*orange1.1t04360*) |
| B74 | GCTTACATGGGAAGAGGATCAAC | AS |  | pJET1.2/blunt |  |
| B205 | ATGGATCTTAAGAGTCTTCC | S |  | pJET1.2/blunt | *CsSesquiTPS7* (*Cs4g11980*) |
| B206 | TTACATGGGAAGAGGATCAA | AS |  | pJET1.2/blunt |  |
| B247 | *ACAAGAGTCC****GGATCC***ccTGTCTTTAGAAGTTTC | S | *Bam*HI/*Sal*I | pET-45b(+) | *CsSesquiTPS1* (*Cs5g23510*) |
| B246 | *CCGCAAGCTT****GTCGAC***TCATATGGGCACAGG | AS |  | pET-45b(+) |  |
| B273 | *ACAAGAGTCC****GGATCC***ccTGTCTTTGGAAGTTTC | S | *Bam*HI | pET-45b(+) | *CsSesquiTPS2* (*Cs4g12120*) |
| B269 | *CTCCCAATTG****GGATCC***TCATATCGGCACAGG | AS |  | pET-45b(+) |  |
| B275 | *ACAAGAGTCCGGATCC*ccTGTCCGCTCAAGTTC | S | *Bam*HI | pET-45b(+) | *CsSesquiTPS4* (*Cs4g12350*) |
| B274 | *CTCCCAATTGGGATCC*TCATATAGTGACAGGG | AS |  | pET-45b(+) |  |
| B284 | *ACAAGAGTCC****GGATCC***ccTGGCTCGAGTTTTTC | S | *Bam*HI | pET-45b(+) | *CsSesquiTPS3* (*orange1.1t03302*) |
| B283 | *CTCCCAATTG****GGATCC***TCAGATCGCAAGGGG | AS |  | pET-45b(+) |  |
| B253 | *ACAAGAGTCC****GGATCC***ccTGTCCGCTCAAG | S | *Bam*HI/*Sal*I | pET-45b(+) | *CsSesquiTPS5* (*Cs4g12400*) |
| B252 | *CCGCAAGCTT****GTCGAC***TCAGATGGTAACAGG | AS |  | pET-45b(+) |  |
| B200 | *ACAAGAGTCC****GGATCC***GAGGGATCTTAAGAGTGTTC | S | *Bam*HI/*Pst*I | pET-45b(+) | *CsSesquiTPS6* (*orange1.1t04360*) |
| B199 | *GCAAGCTTGTCGAC****CTGCAG***TTACATGGGAAGAGGATC | AS |  | pET-45b(+) |  |
| B267 | *ACAAGAGTCC****GGATCC***ccTGGATCTTAAGAGT | S | *Bam*HI | pET-45b(+) | *CsSesquiTPS7* (*Cs4g11980*) |
| B266 | *CTCCCAATTG****GGATCC***TTACATGGGAAGAGG | AS |  | pET-45b(+) |  |
