# Supplementary material for: Genomic Analysis of Terpene Synthase Family and Functional Characterization of Seven Sesquiterpene Synthases from Citrus sinensis
Source: Front Plant Sci. 2017 Aug 24;8:1481. doi: 10.3389/fpls.2017.01481 (PMC5573811; doi:10.3389/fpls.2017.01481)
Supplement: Supplementary Table 2 — Percentage emission of volatiles in different Pineapple sweet orange tissues. Data represent mean values ± SE and are derived from at least two samples replicates. [file Table2.docx]

**Supplementary Table 2.** Percentage emission of volatiles in different Pineapple sweet orange tissues. Data represent mean values ± SE and are derived from at least two samples replicates.

| **Number** | **Compound** | **CAS** | **RT** | **Young leaf** | | **Adult leaf** | | **Mature fruit** | | **Pulp** | | **Flower** | |
| --- | --- | --- | --- | --- | --- | --- | --- | --- | --- | --- | --- | --- | --- |
|  |  |  |  | **Mean %** | **SE** | **Mean %** | **SE** | **Mean %** | **SE** | **Mean %** | **SE** | **Mean %** | **SE** |
| 1 | Butanoic acid, ethyl ester | **105-54-4** | **16.41** |  |  |  |  | 0.83 | 0.29 |  |  |  |  |
| 2 | (*E*)-2-Hexenal | **6728-26-3** | **18.76** |  |  | 0.22 | 0.06 |  |  |  |  |  |  |
| 3 | 1-hexanol | **111-27-3** | **19.17** |  |  |  |  | 0.14 | 0.02 |  |  |  |  |
| 4 | 2-heptanone | **110-43-0** | **20.05** |  |  |  |  | 0.06 | 0.02 |  |  |  |  |
| 5 | Hexanoic acid, methyl ester | **106-70-7** | **21.24** |  |  |  |  | 0.03 | 0.02 |  |  |  |  |
| 6 | α-thujene | **2867-05-2** | **21.83** | 2.46 | 0.93 | 2.01 | 0.18 |  |  |  |  | 0.56 | 0.32 |
| 7 | α-pinene | **7785-70-8** | **22.27** | 3.42 | 0.80 | 2.50 | 0.42 |  |  | 0.91 | 0.00 | 0.51 | 0.11 |
| 8 | α-fenchene | **471-84-1** | **22.95** |  |  | 0.02 | 0.00 |  |  |  |  | 0.01 | 0.00 |
| 9 | Camphene | **79-92-5** | **23.08** |  |  | 0.07 | 0.01 |  |  |  |  |  |  |
| 10 | β-terpinene | **99-84-3** | **23.69** | 44.67 | 2.20 | 15.85 | 0.60 |  |  | 0.99 | 0.03 | 13.07 | 0.41 |
| 11 | β-myrcene | **123-35-3** | **23.90** | 1.42 | 0.04 | 4.11 | 0.02 |  |  | 5.06 | 0.09 | 12.15 | 0.13 |
| 12 | Butanoic acid, butyl ester | **109-21-7** | **23.89** |  |  |  |  | 1.85 | 0.80 |  |  |  |  |
| 13 | Hexanoic acid, ethyl ester | **123-66-0** | **23.99** |  |  |  |  | 11.16 | 2.51 |  |  |  |  |
| 14 | β-pinene | **127-91-3** | **24.14** | 2.87 | 0.24 | 2.90 | 0.21 |  |  |  |  |  |  |
| 15 | 3-hexen-1-ol, acetate (*Z*)- | **3681-71-8** | **24.24** | 4.74 | 1.93 |  |  |  |  |  |  |  |  |
| 16 | Acetic acid, hexyl ester | **142-92-7** | **24.44** |  |  |  |  | 0.48 | 0.10 |  |  |  |  |
| 17 | α-phellandrene | **99-83-2** | **24.94** |  |  | 2.05 | 0.14 |  |  |  |  | 0.18 | 0.06 |
| 18 | 3-Carene | **13466-78-9** | **25.10** | 2.61 | 0.92 | 6.87 | 0.26 |  |  |  |  | 0.55 | 0.21 |
| 19 | 1-hexanol, 2-ethyl | **104-76-7** | **25.17** | 1.80 | 1.71 |  |  |  |  |  |  |  |  |
| 20 | α-terpinene | **99-86-5** | **25.30** | 1.31 | 0.21 | 1.91 | 0.26 |  |  |  |  | 0.24 | 0.12 |
| 21 | (*E*)-β-ocimene | **3779-61-1** | **25.50** | 1.37 | 0.64 | 2.91 | 0.01 | 0.37 | 0.14 |  |  | 0.51 | 0.06 |
| 22 | Limonene | **138-86-3** | **25.75** | 10.76 | 5.82 | 8.04 | 0.08 | 2.49 | 0.97 | 69.29 | 1.41 | 9.00 | 0.29 |
| 23 | β-ocimene | **13877-91-3** | **26.00** | 11.26 | 0.77 | 17.78 | 0.06 | 9.54 | 1.79 | 1.12 | 0.03 | 6.33 | 2.02 |
| 24 | γ-terpinene | **99-85-4** | **26.70** | 1.93 | 0.40 | 2.43 | 0.23 |  |  |  |  | 0.43 | 0.21 |
| 25 | (*Z*)-β-terpineol | **7299-41-4** | **27.22** | 0.30 | 0.13 | 1.08 | 0.29 |  |  |  |  | 0.57 | 0.14 |
| 26 | Butanoic acid, pentyl ester | **540-18-1** | **27.33** |  |  |  |  | 0.08 | 0.04 |  |  |  |  |
| 27 | Heptanoic acid, ethyl ester | **106-30-9** | **27.43** |  |  |  |  | 0.12 | 0.02 |  |  |  |  |
| 28 | 2-carene | **554-61-0** | **27.54** |  |  | 1.17 | 0.02 |  |  |  |  |  |  |
| 29 | Terpinolene | **586-62-9** | **27.70** | 1.00 | 0.20 | 4.00 | 0.11 |  |  | 0.13 | 0.01 |  |  |
| 30 | Linalool | **78-70-6** | **27.83** | 1.83 | 0.81 | 7.28 | 1.08 | 0.13 | 0.03 | 0.85 | 0.08 | 28.58 | 2.08 |
| 31 | Octanoic acid, methyl ester | **111-11-5** | **28.36** |  |  |  |  | 0.65 | 0.54 |  |  |  |  |
| 32 | (*Z*)-*p*-2-menthen-1-ol | **29803-82-5** | **28.36** |  |  | 0.27 | 0.09 |  |  |  |  |  |  |
| 33 | Allo-ocimene | **673-84-7** | **28.73** |  |  | 0.34 | 0.01 |  |  |  |  |  |  |
| 34 | d-2,8-*p*-menthadien-1-ol | **22771-44-4** | **29.00** |  |  | 0.20 | 0.03 |  |  | 0.06 | 0.00 |  |  |
| 35 | Perilla alcohol | **536-59-4** | **29.10** |  |  |  |  | 0.14 | 0.07 |  |  |  |  |
| 36 | α-ionol | **25312-34-9** | **29.23** |  |  | 0.16 | 0.00 |  |  | 0.03 | 0.00 |  |  |
| 37 | Citronellal | **2385-77-5** | **29.55** | 3.49 | 3.42 | 0.23 | 0.09 |  |  | 0.07 | 0.00 |  |  |
| 38 | (*E*)-Butanoic acid, 3-hexenyl ester | **53398-84-8** | **30.26** |  |  |  |  | 0.08 | 0.02 |  |  |  |  |
| 39 | (*Z*)-Butanoic acid, 3-hexenyl ester | **16491-36-4** | **30.43** |  |  |  |  | 4.56 | 0.21 |  |  |  |  |
| 40 | Hexanoic acid, hexyl ester | **6378-65-0** | **30.58** |  |  |  |  | 12.10 | 3.31 |  |  |  |  |
| 41 | Octanoic acid, ethyl ester | **106-32-1** | **30.69** |  |  |  |  | 12.98 | 0.58 |  |  |  |  |
| 42 | 4-Terpineol | **562-74-3** | **31.08** | 0.51 | 0.30 | 1.26 | 0.19 |  |  |  |  |  |  |
| 43 | Acetic acid, octyl ester | **112-14-1** | **31.09** |  |  |  |  |  |  | 0.53 | 0.00 |  |  |
| 44 | Decanal | **112-31-2** | **31.27** | 0.60 | 0.48 |  |  |  |  | 0.16 | 0.00 |  |  |
| 45 | α-terpineol | **98-55-5** | **31.44** | 0.55 | 0.20 | 1.26 | 0.17 |  |  | 0.14 | 0.00 | 0.96 | 0.58 |
| 46 | (*Z*)-carveol | **1197-06-4** | **31.58** |  |  |  |  |  |  | 0.12 | 0.00 |  |  |
| 47 | β-citronellol | **106-22-9** | **31.81** |  |  | 0.45 | 0.04 |  |  |  |  |  |  |
| 48 | Nerol | **106-25-2** | **31.95** |  |  | 0.75 | 0.02 |  |  |  |  |  |  |
| 49 | Butanoic acid, 2-methyl-, hexyl ester | **10032-15-2** | **32.00** |  |  |  |  | 0.08 | 0.00 |  |  |  |  |
| 50 | Carvone | **99-49-0** | **32.04** |  |  |  |  |  |  | 0.07 | 0.01 |  |  |
| 51 | (*E*)-2-hexenoic acid, butyl ester | **54411-16-4** | **32.19** |  |  |  |  | 0.27 | 0.17 |  |  |  |  |
| 52 | β-cyclocitral | **432-25-7** | **32.33** |  |  |  |  | 0.37 | 0.17 |  |  |  |  |
| 53 | Neral | **106-26-3** | **32.45** |  |  | 0.48 | 0.10 |  |  |  |  |  |  |
| 54 | Hexanoic acid, 2-methylbutyl ester | **2601-13-0** | **32.50** |  |  |  |  | 0.07 | 0.03 |  |  |  |  |
| 55 | Geraniol | **106-24-1** | **32.60** |  |  | 0.22 | 0.00 |  |  |  |  |  |  |
| 56 | Geranial | **141-27-5** | **33.29** |  |  | 0.77 | 0.15 |  |  |  |  |  |  |
| 57 | Hexanoic acid, pentyl ester | **540-07-8** | **33.55** |  |  |  |  | 0.08 | 0.05 |  |  |  |  |
| 58 | Butanoic acid, heptyl ester | **5870-93-9** | **33.58** |  |  |  |  | 0.05 | 0.03 |  |  |  |  |
| 59 | Hexanoic acid, 3-methyl-2-butenyl ester | **76649-22-4** | **33.69** |  |  |  |  | 0.06 | 0.03 |  |  |  |  |
| 60 | Butanoic acid, 2-octyl ester | **20286-44-6** | **34.02** |  |  |  |  | 0.45 | 0.03 |  |  |  |  |
| 61 | Perilla aldehyde | **2111-75-3** | **34.16** |  |  |  |  |  |  | 0.33 | 0.02 |  |  |
| 62 | Carveol acetate | **97-42-7** | **34.98** |  |  |  |  |  |  | 0.13 | 0.01 |  |  |
| 63 | Geranyl acetate | **105-87-3** | **34.68** |  |  | 0.05 | 0.00 |  |  |  |  |  |  |
| 64 | (*Z*)-carvyl acetate | **1205-42-1** | **35.15** |  |  |  |  |  |  | 0.07 | 0.01 |  |  |
| 65 | Citronellyl acetate | **150-84-5** | **35.33** |  |  |  |  |  |  | 0.10 | 0.01 |  |  |
| 66 | Neryl acetate | **141-12-8** | **35.61** |  |  |  |  |  |  | 0.11 | 0.02 |  |  |
| 67 | (-)-Perillyl alcohol | **18457-55-1** | **35.76** |  |  |  |  |  |  | 0.20 | 0.14 |  |  |
| 68 | α-cubebene | **17699-14-8** | **36.06** | 0.19 | 0.13 | 0.03 | 0.02 |  |  | 0.13 | 0.01 |  |  |
| 69 | Methyl anthranilate | **134-20-3** | **36.12** |  |  |  |  |  |  |  |  | 12.0 | 0.7 |
| 70 | Linalyl formate | **115-99-1** | **36.18** |  |  |  |  |  |  | 0.08 | 0.01 |  |  |
| 71 | (*Z*)-hexanoic acid, 3-hexenyl ester | **31501-11-8** | **36.27** |  |  |  |  | 1.09 | 0.30 |  |  |  |  |
| 72 | Cyclohexene, 2-ethenyl-1,3,3-trimethyl- | **5293-90-3** | **36.49** |  |  | 0.03 | 0.02 |  |  |  |  |  |  |
| 73 | Decanoic acid, ethyl ester | **110-38-3** | **37.09** |  |  |  |  | 0.14 | 0.05 |  |  |  |  |
| 74 | α-copaene | **3856-25-5** | **37.06** |  |  | 0.36 | 0.00 | 0.20 | 0.01 | 0.53 | 0.01 | 0.20 | 0.06 |
| 75 | β-elemene | **515-13-9** | **37.30** |  |  | 3.44 | 0.01 | 2.48 | 0.05 | 0.39 | 0.00 | 1.82 | 0.58 |
| 76 | β-cubebene | **13744-15-5** | **37.30** |  |  |  |  |  |  | 0.29 | 0.01 |  |  |
| 77 | *p*-menth-1-en-9-ol | **18479-68-0** | **37.76** |  |  |  |  |  |  | 0.13 | 0.01 |  |  |
| 78 | (*Z*)-β-farnesene | **28973-97-7** | **38.39** |  |  | 1.01 | 0.38 |  |  |  |  | 1.65 | 0.55 |
| 79 | β-caryophyllene | **87-44-5** | **38.59** | 0.89 | 0.06 | 3.53 | 0.02 | 1.28 | 0.32 | 0.37 | 0.02 | 2.47 | 0.53 |
| 80 | β-cedrene | **546-28-1** | **38.78** |  |  | 0.18 | 0.05 |  |  |  |  | 0.13 | 0.05 |
| 81 | Allo-aromadendrene | **25246-27-9** | **39.10** |  |  |  |  |  |  | 0.29 | 0.00 |  |  |
| 82 | γ-gurjunene | **22567-17-5** | **39.13** |  |  |  |  | 1.37 | 0.11 |  |  |  |  |
| 83 | α-humulene | **6753-98-6** | **39.56** |  |  | 0.82 | 0.01 | 0.17 | 0.02 | 0.10 | 0.00 | 0.36 | 0.08 |
| 84 | γ-selinene | **515-17-3** | **39.73** |  |  |  |  |  |  | 0.10 | 0.00 |  |  |
| 85 | α-farnesene | **502-61-4** | **39.75** |  |  | 0.34 | 0.01 |  |  |  |  | 0.45 | 0.12 |
| 86 | 3,7(11)-selinadiene | **6813-21-4** | **39.99** |  |  |  |  | 0.71 | 0.07 | 0.25 | 0.00 |  |  |
| 87 | β-selinene | **17066-67-0** | **40.19** |  |  |  |  | 3.06 | 0.18 | 0.86 | 0.04 | 0.02 | 0.01 |
| 88 | Valencene | **4630-07-3** | **40.42** |  |  |  |  | 22.05 | 3.88 | 11.73 | 0.00 |  |  |
| 89 | Eremophilene | **10219-75-7** | **40.44** |  |  | 0.04 | 0.02 |  |  |  |  | 0.04 | 0.01 |
| 90 | α-selinene | **473-13-2** | **40.55** |  |  | 0.04 | 0.02 | 1.98 | 0.18 | 0.74 | 0.00 | 0.02 | 0.02 |
| 91 | δ-selinene | **473-14-3** | **40.67** |  |  |  |  |  |  | 0.38 | 0.02 | 0.14 | 0.06 |
| 92 | β-cadinene | **523-47-7** | **40.73** |  |  | 0.14 | 0.00 | 1.62 | 0.07 | 0.33 | 0.00 |  |  |
| 93 | α-panasinsene | **56633-28-4** | **41.25** |  |  |  |  | 2.75 | 0.16 | 0.84 | 0.00 |  |  |
| 94 | (+/-)-trans-nerolidol | **40716-66-3** | **41.32** |  |  |  |  | 0.66 | 0.03 |  |  | 6.84 | 2.26 |
| 95 | Octanoic acid, hexyl ester | **1117-55-1** | **41.52** |  |  |  |  | 0.87 | 0.32 |  |  |  |  |
| 96 | α-sinensal | **4955-32-2** | **44.77** |  |  | 0.28 | 0.02 |  |  |  |  |  |  |
| 97 | Juniper camphor | **473-04-1** | **45.08** |  |  |  |  | 0.36 | 0.06 | 0.08 | 0.01 |  |  |
| 98 | Farnesol | **4602-84-0** | **45.12** |  |  |  |  |  |  |  |  | 0.22 | 0.03 |
| 99 | β-sinensal | **3779-62-2** | **46.15** |  |  | 0.13 | 0.00 |  |  |  |  |  |  |
