# Supplementary material for: Genomic Analysis of Terpene Synthase Family and Functional Characterization of Seven Sesquiterpene Synthases from Citrus sinensis
Source: Front Plant Sci. 2017 Aug 24;8:1481. doi: 10.3389/fpls.2017.01481 (PMC5573811; doi:10.3389/fpls.2017.01481)
Supplement: Supplementary Figure 1 — Alignment of the deduced protein sequences of putative full-length Citrus sinensis TPS genes with previously characterized CsTPSs. Q94JS8, (E)-β-farnesene synthase from Citrus junos; AAQ04608, valencene synthase from Citrus sinensis; BAP75561, linalool synthase from Citrus unshiu; BAP75559, and BAP75560, linalool synthase from Citrus unshiu; BAF73932, and BAF73932, D-limonene synthase from Citrus jambhiri; BAD27256, and BAD27257, D-limonene synthase from Citrus unshiu; AOP12358, D-limonene synthase from Citrus sinensis; AAM53943, γ-terpinene synthase from Citrus limon; BAD27258, and BAD27259, γ-terpinene synthase from Citrus unshiu; AAM53944, and AAM53946, D-limonene synthase 1 from Citrus limon; AAM53945, β-pinene synthase from Citrus limon; BAM29049, geraniol synthase from Citrus jambhiri; BAD91046, (E)-β-ocimene synthase from Citrus unshiu; BAD91045, 1,8-cineole synthase from Citrus unshiu; BAF73933, β-pinene synthase from Citrus jambhiri, BAD27260, β-pinene synthase from Citrus unshiu; CuSTS-1, germacrene-A synthase from Citrus unshiu; BAP74389, δ-elemene synthase from Citrus jambhiri. TPS conserved motifs are bold lettered. [file SupplementaryFigure1.DOCX]

Cs2g22180 ------------------------------------------------------------ 0

Cs2g07250 ------------------------------------------------------------ 0

BAP75559 ------------------------------------------------------------ 0

Cs2g07240 ------------------------------------------------------------ 0

BAP75560 ------------------------------------------------------------ 0

orange1.1t02448 ------------------------------------------------------------ 0

orange1.1t03307 ------------------------------------------------------------ 0

orange1.1t02008 ------------------------------------------------------------ 0

Cs4g11980 ------------------------------------------------------------ 0

orange1.1t04360.1 ------------------------------------------------------------ 0

orange1.1t04360.2 ------------------------------------------------------------ 0

Cs4g12080 ------------------------------------------------------------ 0

Cs4g12050 ------------------------------------------------------------ 0

Cs4g12060 ------------------------------------------------------------ 0

Cs5g12880 ------------------------------------------------------------ 0

Cs5g12900.2 ------------------------------------------------------------ 0

AAQ04608 ------------------------------------------------------------ 0

Cs4g12350 ------------------------------------------------------------ 0

Cs4g12400 ------------------------------------------------------------ 0

Cs4g12220 ------------------------------------------------------------ 0

Cs4g12480 ------------------------------------------------------------ 0

Cs4g11320 ------------------------------------------------------------ 0

orange1.1t03302.1 ------------------------------------------------------------ 0

Q94JS8 ------------------------------------------------------------ 0

Cs4g12450 ------------------------------------------------------------ 0

orange1.1t00017.2 ------------------------------------------------------------ 0

Cs5g06290 ------------------------------------------------------------ 0

Cs3g21560 ------------------------------------------------------------ 0

Cs3g21590c ------------------------------------------------------------ 0

Cs4g12110.1 ------------------------------------------------------------ 0

BAP75561 ------------------------------------------------------------ 0

Cs3g16210 ------------------------------------------------------------ 0

Cs2g23470 ------------------------------------------------------------ 0

Cs2g24110.1 ------------------------------------------------------------ 0

Cs5g23510.1 ------------------------------------------------------------ 0

Cs5g23540 ------------------------------------------------------------ 0

BAP74389 ------------------------------------------------------------ 0

Cs4g12090 ------------------------------------------------------------ 0

CuSTS1 ------------------------------------------------------------ 0

Cs4g12120.1 ------------------------------------------------------------ 0

Cs4g12120.2 ------------------------------------------------------------ 0

Cs2g22090 ------------------------------------------------------------ 0

Cs2g22100.1 ------------------------------------------------------------ 0

Cs2g07280 ------------------------------------------------------------ 0

Cs2g24530.1 ------------------------------------------------------------ 0

Cs5g22980 ------------------------------------------------------------ 0

Cs3g04340 ------------------------------------------------------------ 0

BAD27256 ------------------------------------------------------------ 0

AAM53944 ------------------------------------------------------------ 0

BAF73932 ------------------------------------------------------------ 0

AAM53946 ------------------------------------------------------------ 0

BAD27257 ------------------------------------------------------------ 0

AOP12358 ------------------------------------------------------------ 0

Cs3g04170 ------------------------------------------------------------ 0

Cs3g04360 ------------------------------------------------------------ 0

Cs3g04190 ------------------------------------------------------------ 0

orange1.1t04775.1 ------------------------------------------------------------ 0

orange1.1t04366 ------------------------------------------------------------ 0

BAM29049 ------------------------------------------------------------ 0

Cs2g22150 ------------------------------------------------------------ 0

BAD27258 ------------------------------------------------------------ 0

BAD27259 ------------------------------------------------------------ 0

Cs3g07850 ------------------------------------------------------------ 0

AAM53943 ------------------------------------------------------------ 0

Cs7g18530 ------------------------------------------------------------ 0

Cs3g07920 ------------------------------------------------------------ 0

BAF73933 ------------------------------------------------------------ 0

AAM53945 ------------------------------------------------------------ 0

BAD27260 ------------------------------------------------------------ 0

BAD91045 ------------------------------------------------------------ 0

Cs3g07880 ------------------------------------------------------------ 0

Cs7g17640 ------------------------------------------------------------ 0

Cs2g03570 ------------------------------------------------------------ 0

Cs8g14120.1 ------------------------------------------------------------ 0

BAD91046 ------------------------------------------------------------ 0

Cs5g15530 ------------------------------------------------------------ 0

Cs5g31210 MSSHYYSTNLFYPVVPSGTRRSNSSSFNSELPSFWPFQTKEKRINFNVRPRCSAISRPLI 60

Cs2g06470 ------------------------------------------------------------ 0

orange1.1t03278 ------------------------------------------------------------ 0

Cs2g22180 ------------------------------------------------------------ 0

Cs2g07250 ------------------------------------------------------------ 0

BAP75559 ------------------------------------------------------------ 0

Cs2g07240 ------------------------------------------------------------ 0

BAP75560 ------------------------------------------------------------ 0

orange1.1t02448 ------------------------------------------------------------ 0

orange1.1t03307 ------------------------------------------------------------ 0

orange1.1t02008 ------------------------------------------------------------ 0

Cs4g11980 ------------------------------------------------------------ 0

orange1.1t04360.1 ------------------------------------------------------------ 0

orange1.1t04360.2 ------------------------------------------------------------ 0

Cs4g12080 ------------------------------------------------------------ 0

Cs4g12050 ------------------------------------------------------------ 0

Cs4g12060 ------------------------------------------------------------ 0

Cs5g12880 ------------------------------------------------------------ 0

Cs5g12900.2 ------------------------------------------------------------ 0

AAQ04608 ------------------------------------------------------------ 0

Cs4g12350 ------------------------------------------------------------ 0

Cs4g12400 ------------------------------------------------------------ 0

Cs4g12220 ------------------------------------------------------------ 0

Cs4g12480 ------------------------------------------------------------ 0

Cs4g11320 ------------------------------------------------------------ 0

orange1.1t03302.1 ------------------------------------------------------------ 0

Q94JS8 ------------------------------------------------------------ 0

Cs4g12450 ------------------------------------------------------------ 0

orange1.1t00017.2 ------------------------------------------------------------ 0

Cs5g06290 ------------------------------------------------------------ 0

Cs3g21560 ------------------------------------------------------------ 0

Cs3g21590c ------------------------------------------------------------ 0

Cs4g12110.1 ------------------------------------------------------------ 0

BAP75561 ------------------------------------------------------------ 0

Cs3g16210 ------------------------------------------------------------ 0

Cs2g23470 ------------------------------------------------------------ 0

Cs2g24110.1 ------------------------------------------------------------ 0

Cs5g23510.1 ------------------------------------------------------------ 0

Cs5g23540 ------------------------------------------------------------ 0

BAP74389 ------------------------------------------------------------ 0

Cs4g12090 ------------------------------------------------------------ 0

CuSTS1 ------------------------------------------------------------ 0

Cs4g12120.1 ------------------------------------------------------------ 0

Cs4g12120.2 ------------------------------------------------------------ 0

Cs2g22090 ------------------------------------------------------------ 0

Cs2g22100.1 ------------------------------------------------------------ 0

Cs2g07280 ------------------------------------------------------------ 0

Cs2g24530.1 ------------------------------------------------------------ 0

Cs5g22980 ------------------------------------------------------------ 0

Cs3g04340 ------------------------------------------------------------ 0

BAD27256 ------------------------------------------------------------ 0

AAM53944 ------------------------------------------------------------ 0

BAF73932 ------------------------------------------------------------ 0

AAM53946 ------------------------------------------------------------ 0

BAD27257 ------------------------------------------------------------ 0

AOP12358 ------------------------------------------------------------ 0

Cs3g04170 ------------------------------------------------------------ 0

Cs3g04360 ------------------------------------------------------------ 0

Cs3g04190 ------------------------------------------------------------ 0

orange1.1t04775.1 ------------------------------------------------------------ 0

orange1.1t04366 ------------------------------------------------------------ 0

BAM29049 ------------------------------------------------------------ 0

Cs2g22150 ------------------------------------------------------------ 0

BAD27258 ------------------------------------------------------------ 0

BAD27259 ------------------------------------------------------------ 0

Cs3g07850 ------------------------------------------------------------ 0

AAM53943 ------------------------------------------------------------ 0

Cs7g18530 ------------------------------------------------------------ 0

Cs3g07920 ------------------------------------------------------------ 0

BAF73933 ------------------------------------------------------------ 0

AAM53945 ------------------------------------------------------------ 0

BAD27260 ------------------------------------------------------------ 0

BAD91045 ------------------------------------------------------------ 0

Cs3g07880 ------------------------------------------------------------ 0

Cs7g17640 ------------------------------------------------------------ 0

Cs2g03570 ------------------------------------------------------------ 0

Cs8g14120.1 ------------------------------------------------------------ 0

BAD91046 ------------------------------------------------------------ 0

Cs5g15530 ---------------------MDDNQVEVLEVSASNEIKKLDHIIKSMLGSMDG-----G 34

Cs5g31210 QGEYADVFQNGLPVIKWKEIVEDDIEEDRIEVCASNEIKERVHAIKSMLGSMED-----G 115

Cs2g06470 -------------------------------MELPQLCNIQALVKEIKGGMFSNIDYPNS 29

orange1.1t03278 -------MILDIEAIRWRPGTANHGSKVGTEAKTNNLCFQR--TKERTEKMFDK-----I 46

**SAYDTAW** **Q**XX**DGSW**

Cs2g22180 ------------------------------------------------------------ 0

Cs2g07250 ------------------------------------------------------------ 0

BAP75559 ------------------------------------------------------------ 0

Cs2g07240 ------------------------------------------------------------ 0

BAP75560 ------------------------------------------------------------ 0

orange1.1t02448 ------------------------------------------------------------ 0

orange1.1t03307 ------------------------------------------------------------ 0

orange1.1t02008 ------------------------------------------------------------ 0

Cs4g11980 ------------------------------------------------------------ 0

orange1.1t04360.1 ------------------------------------------------------------ 0

orange1.1t04360.2 ------------------------------------------------------------ 0

Cs4g12080 ------------------------------------------------------------ 0

Cs4g12050 ------------------------------------------------------------ 0

Cs4g12060 ------------------------------------------------------------ 0

Cs5g12880 ------------------------------------------------------------ 0

Cs5g12900.2 ------------------------------------------------------------ 0

AAQ04608 ------------------------------------------------------------ 0

Cs4g12350 ------------------------------------------------------------ 0

Cs4g12400 ------------------------------------------------------------ 0

Cs4g12220 ------------------------------------------------------------ 0

Cs4g12480 ------------------------------------------------------------ 0

Cs4g11320 ------------------------------------------------------------ 0

orange1.1t03302.1 ------------------------------------------------------------ 0

Q94JS8 ------------------------------------------------------------ 0

Cs4g12450 ------------------------------------------------------------ 0

orange1.1t00017.2 ------------------------------------------------------------ 0

Cs5g06290 ------------------------------------------------------------ 0

Cs3g21560 ------------------------------------------------------------ 0

Cs3g21590c ------------------------------------------------------------ 0

Cs4g12110.1 ------------------------------------------------------------ 0

BAP75561 ------------------------------------------------------------ 0

Cs3g16210 ------------------------------------------------------------ 0

Cs2g23470 ------------------------------------------------------------ 0

Cs2g24110.1 ------------------------------------------------------------ 0

Cs5g23510.1 ------------------------------------------------------------ 0

Cs5g23540 ------------------------------------------------------------ 0

BAP74389 ------------------------------------------------------------ 0

Cs4g12090 ------------------------------------------------------------ 0

CuSTS1 ------------------------------------------------------------ 0

Cs4g12120.1 ------------------------------------------------------------ 0

Cs4g12120.2 ------------------------------------------------------------ 0

Cs2g22090 ------------------------------------------------------------ 0

Cs2g22100.1 ------------------------------------------------------------ 0

Cs2g07280 ------------------------------------------------------------ 0

Cs2g24530.1 ------------------------------------------------------------ 0

Cs5g22980 ------------------------------------------------------------ 0

Cs3g04340 ------------------------------------------------------------ 0

BAD27256 ------------------------------------------------------------ 0

AAM53944 ------------------------------------------------------------ 0

BAF73932 ------------------------------------------------------------ 0

AAM53946 ------------------------------------------------------------ 0

BAD27257 ------------------------------------------------------------ 0

AOP12358 ------------------------------------------------------------ 0

Cs3g04170 ------------------------------------------------------------ 0

Cs3g04360 ------------------------------------------------------------ 0

Cs3g04190 ------------------------------------------------------------ 0

orange1.1t04775.1 ------------------------------------------------------------ 0

orange1.1t04366 ------------------------------------------------------------ 0

BAM29049 ------------------------------------------------------------ 0

Cs2g22150 ------------------------------------------------------------ 0

BAD27258 ------------------------------------------------------------ 0

BAD27259 ------------------------------------------------------------ 0

Cs3g07850 ------------------------------------------------------------ 0

AAM53943 ------------------------------------------------------------ 0

Cs7g18530 ------------------------------------------------------------ 0

Cs3g07920 ------------------------------------------------------------ 0

BAF73933 ------------------------------------------------------------ 0

AAM53945 ------------------------------------------------------------ 0

BAD27260 ------------------------------------------------------------ 0

BAD91045 ------------------------------------------------------------ 0

Cs3g07880 ------------------------------------------------------------ 0

Cs7g17640 ------------------------------------------------------------ 0

Cs2g03570 ------------------------------------------------------------ 0

Cs8g14120.1 ------------------------------------------------------------ 0

BAD91046 ------------------------------------------------------------ 0

Cs5g15530 EIST**S**AY**DTAW**VALIKDIDGN-DAPQFPSCLQWIADN**Q**LP**DGSW**GDDK--IFLAHDRLIN 91

Cs5g31210 EISV**SAYDTAW**VALVEDIQGS-GAPQFPSSLHWIANS**Q**LP**DGSW**GDHL--LFSAHDRLIN 172

Cs2g06470 FVSP**SAYDTAW**LAMIPDSEQPFSRPMFENCLNWVLNN**Q**RE**DGSW**GELDGHGNNTIESLPA 89

orange1.1t03278 ELSV**S**P**YDTAW**VAMVPSLELP-QAPCFPQCINWLLDN**Q**VN**DGSW**GLHNRPSWLVKDAVLC 105

Cs2g22180 -------------------------------------------------MP--------- 2

Cs2g07250 -----------------------------------------------MAFSSSS------ 7

BAP75559 -----------------------------------------------MAFSSSS------ 7

Cs2g07240 -----------------------------------------------MAFPSKI------ 7

BAP75560 -----------------------------------------------MAFSSKD------ 7

orange1.1t02448 -----------------------------------------------MAFSSKD------ 7

orange1.1t03307 -----------------------------------------------MAFSSKD------ 7

orange1.1t02008 ---------------------------------------------MA---LQAL------ 6

Cs4g11980 ------------------------------------------------------------ 0

orange1.1t04360.1 ------------------------------------------------------------ 0

orange1.1t04360.2 ------------------------------------------------------------ 0

Cs4g12080 ------------------------------------------------------------ 0

Cs4g12050 ------------------------------------------------------------ 0

Cs4g12060 ------------------------------------------------------------ 0

Cs5g12880 ------------------------------------------------------------ 0

Cs5g12900.2 ------------------------------------------------------------ 0

AAQ04608 ------------------------------------------------------------ 0

Cs4g12350 ------------------------------------------------------------ 0

Cs4g12400 ------------------------------------------------------------ 0

Cs4g12220 ------------------------------------------------------------ 0

Cs4g12480 ------------------------------------------------------------ 0

Cs4g11320 ------------------------------------------------------------ 0

orange1.1t03302.1 ------------------------------------------------------------ 0

Q94JS8 ------------------------------------------------------------ 0

Cs4g12450 ------------------------------------------------------------ 0

orange1.1t00017.2 ------------------------------------------------------------ 0

Cs5g06290 ------------------------------------------------------------ 0

Cs3g21560 ------------------------------------------------------------ 0

Cs3g21590c ------------------------------------------------------------ 0

Cs4g12110.1 ------------------------------------------------------------ 0

BAP75561 ------------------------------------------------------------ 0

Cs3g16210 -----------------MCNVRLRLLLNLRAVKSIKCTSLKAREDHT---VQLL------ 34

Cs2g23470 ------------------------------------------------------------ 0

Cs2g24110.1 ------------------------------------------------------------ 0

Cs5g23510.1 ------------------------------------------------------------ 0

Cs5g23540 ------------------------------------------------------------ 0

BAP74389 ------------------------------------------------------------ 0

Cs4g12090 ------------------------------------------------------------ 0

CuSTS1 ------------------------------------------------------------ 0

Cs4g12120.1 ------------------------------------------------------------ 0

Cs4g12120.2 ------------------------------------------------------------ 0

Cs2g22090 ------------------------------------------------------------ 0

Cs2g22100.1 ------------------------MELKLASLAYSSCVVNVQTC-------KIL------ 23

Cs2g07280 ------------------------------------------------------------ 0

Cs2g24530.1 ------------------------------------------------------------ 0

Cs5g22980 --------------------------------------------------MSSS------ 4

Cs3g04340 ------------------------------------------------------------ 0

BAD27256 --------------------------------------------------MSSC------ 4

AAM53944 --------------------------------------------------MSSC------ 4

BAF73932 --------------------------------------------------MSSC------ 4

AAM53946 --------------------------------------------------MSSC------ 4

BAD27257 --------------------------------------------------MSSC------ 4

AOP12358 --------------------------------------------------MSSC------ 4

Cs3g04170 --------------------------------------------------MSSC------ 4

Cs3g04360 --------------------------------------------------MSSC------ 4

Cs3g04190 --------------------------------------------------MSSC------ 4

orange1.1t04775.1 ------------------------------------------------------------ 0

orange1.1t04366 ------------------------------------------------------------ 0

BAM29049 --------------------------------------------------MSSS------ 4

Cs2g22150 ------------------------------------------------------------ 0

BAD27258 ------------------------MALNLLSSLPAAGNFTILSLPLSS------------ 24

BAD27259 ------------------------MALNLLSSLPAAGNFTILSLPLSS------------ 24

Cs3g07850 ------------------------MALNLLSSLPAACNFTRLSLPLSS------------ 24

AAM53943 ------------------------MALNLLSSLPAACNFTRLSLPLSS------------ 24

Cs7g18530 ------------------------MALNLLSSLPAACNFTRLSLPLSS------------ 24

Cs3g07920 ------------------------MALNLLSSLPAACNFTRLSLPLSS------------ 24

BAF73933 ------------------------MALNLLSSIPAACNFTRLSLPLSS------------ 24

AAM53945 ------------------------MALNLLSSIPAACNFTRLSLPLSS------------ 24

BAD27260 ------------------------MALNLLSSLPAAGNFTILSLPLSS------------ 24

BAD91045 ------------------------MALNLLSSLPAACNFTRLSLPLSS------------ 24

Cs3g07880 ------------------------MALNLLSPLPATCNFTRLSFRLSS------------ 24

Cs7g17640 ------------------------MALNLLSSLPATCNFTRLSLRLSS------------ 24

Cs2g03570 ------------------------MAHQLIASVPAANFTRLQPRSCISSVG--------- 27

Cs8g14120.1 ------------------------MAHQLMTSVPAL-TRLQEPRSFISSLGSPS------ 29

BAD91046 ------------------------MAHQLMTSVPAL-TRLQEPRSFISSLGSP------- 28

Cs5g15530 TLACIVALKSWNIHL-DKCEKGISFVKGNLSKLE-NENEEHTTCGFEVAFPSLLEIARSL 149

Cs5g31210 TLACVVALRSWNIHH-DKCHKGLLFFKENISKLE-NENDEHMPIGFEVAFPSLLEIARSL 230

Cs2g06470 TLACIIVLKRWNSGNPHQIQNGLDYVRANVEKLVGDNNYEQRPRWFAIVFPAMLELASAV 149

orange1.1t03278 TLACVLALKRWGIGE-EQMNKGIQFIMSNFASV--TDEKQQTPVGFDIIFPGMIECAQDL 162

**RR**X

Cs2g22180 ----------TTSCVSLRLGPLVSLRTRSWQQGRSL-------------HSN-SLSCICQ 38

Cs2g07250 -----RAKLSGTSHISKDPDKISKTSRPNLIEFTPS-------------PTIYQKGCITS 49

BAP75559 -----RAKLSATSHISKAPDKISKTSRPSLIEFTPS-------------PTIYQKGCITS 49

Cs2g07240 -----I--SSASSHVQVIPEQTSKVGKQNLSDINSL-------------LPN-NKNGNIN 46

BAP75560 -----I--SSDSSHIHFIPKHISKVGNRNLNNINSL-------------LPN-NKKGSIN 46

orange1.1t02448 -----I--SSDSSHIQVIPKHISKVGNPNLSNINSF-------------MPN-NKKGNIN 46

orange1.1t03307 -----I--SSDSSHIQVIPKHISKVGNPNLSNINSV-------------LPN-NKKGNIN 46

orange1.1t02008 -----VPSSFLQSVSLSFTKVHPNFFY--NNQLSCKTNSPS--KVLCLAYKNNQEND**RP**L 57

Cs4g11980 -------------------------------------MDLK--SLPSSKESTKADEN**RR**S 21

orange1.1t04360.1 ------------------------------------MRDLK--SVLSSKESTKADVN**RR**S 22

orange1.1t04360.2 ------------------------------------MRDLK--SVLSSKESTKADVN**RR**S 22

Cs4g12080 ---------------------------------MALQDSEV--PSS---ILNATAGN**RP**T 22

Cs4g12050 ---------------------------------MALQDSEV--PAS---ILNATGGN**RP**T 22

Cs4g12060 ---------------------------------MALQDSEV--PSS---ILNATAGN**RP**T 22

Cs5g12880 --------------------------------------------------MSSGETF**RP**T 10

Cs5g12900.2 --------------------------------------------------MSSGETF**RP**T 10

AAQ04608 --------------------------------------------------MSSGETF**RP**T 10

Cs4g12350 ---------------------------------MSAQ--VL--ATV---SSSTEKTV**RP**I 20

Cs4g12400 ---------------------------------MSAQ--VL--ATV---SSSTEKTV**RP**I 20

Cs4g12220 ------------------------------------------------------------ 0

Cs4g12480 ------------------------------------M--LL--AAV---SSSTEETV**RP**I 17

Cs4g11320 ---------------------------------MSIP--LL--AAV---SSSTEETV**RP**I 20

orange1.1t03302.1 ------------------------------MKDMSIP--LL--AAV---SSSTEETV**RP**I 23

Q94JS8 ------------------------------MKDMSIP--LL--AAV---SSSTEETV**RP**I 23

Cs4g12450 ---------------------------------------MS--TPV---PTVVEETV**RP**I 16

orange1.1t00017.2 ----------------------------------MST--PV--PAA---VSSSIGEA**RP**I 19

Cs5g06290 -------------------------------------MSIQ--VPQISSQNAKSQVM**RR**T 21

Cs3g21560 -------------------------------------------------MPKGGDID**RR**T 11

Cs3g21590c -------------------------------------------------MSEGGDFN**RR**T 11

Cs4g12110.1 ---------------------------------MSFPVSAS--PNKVIRINAEKEST**RR**S 25

BAP75561 ---------------------------------MLFQVSAS--PNKVIRINAEKEST**RR**S 25

Cs3g16210 -----KHKKLRQELINLFTSKLKSLQK--AIENMSLQVSAS--PTKIIQRNAEKDYT**RR**S 85

Cs2g23470 ---------------------------------MSLEVSAS--SAKVI-QNSGKDST**RR**S 24

Cs2g24110.1 ------------------------------------------------------------ 0

Cs5g23510.1 ---------------------------------MSLEVSAS--PAKVI-QNAGKDST**RG**S 24

Cs5g23540 ---------------------------------MSLEVSAC--PAKII-QNAGKDST**RG**S 24

BAP74389 ---------------------------------MSFAVSAS--PAKVI-QNAGKDTT**RR**S 24

Cs4g12090 ---------------------------------MSLQVSAS--PTKIIQRNAEKDST**RR**S 25

CuSTS1 ---------------------------------MSLEVSAP--PA-KVIQNAGKDST**RR**S 24

Cs4g12120.1 ---------------------------------MSLEVSAS--PA-KVIQNAGKDST**RR**S 24

Cs4g12120.2 ---------------------------------MSLEVSAS--PA-KVIQNAGKDST**RR**S 24

Cs2g22090 -------------------MACSN-------------------NSIVACNGSNDPLQ**RR**S 22

Cs2g22100.1 -----FSPSVAARGKNGRSRLIRN-------------------IASNNSTKPLQAKQ**RR**S 59

Cs2g07280 -------------------------MEQGKQLGHQKQL-VL--SCQMNSKACNVIPP**RQ**S 32

Cs2g24530.1 ----------------------------------------------MNSKACNVIPP**RQ**S 14

Cs5g22980 -----IIPSTFIISVNGFKLCPPLATNRVTTTRMSEYK-PA--QCFASAQPDTAAVV**RR**S 56

Cs3g04340 ------------------------------------------------------------ 0

BAD27256 -----INPSTLVTSVNGFK-CLPLATNKAAIRIMAKNK-PV--QCLVSAKYDNLTVD**RR**S 55

AAM53944 -----INPSTLVTSVNAFK-CLPLATNKAAIRIMAKYK-PV--QCLISAKYDNLTVD**RR**S 55

BAF73932 -----INPSTLVTSVNAFK-CLPLATNKAAIRIMAKYK-PV--QCLISAKYDNLTVD**RR**S 55

AAM53946 -----INPSTLVTSANGFK-CLPLATNKAAIRIMAKNK-PV--QCLVSAKYDNLIVD**RR**S 55

BAD27257 -----INPSTLATSVNGFK-CLPLATNRAAIRIMAKNK-PV--QCLVSTKYDNLTVD**RR**S 55

AOP12358 -----INPSTLATSVNGFK-CLPLATNRAAIRIMAKNK-PV--QCLVSTKYDNLTVD**RR**S 55

Cs3g04170 -----INPSTLVTSINGFK-CLPLATNKAAIRIMAKNK-PV--QCLVSAKYDNLTVD**RR**S 55

Cs3g04360 -----INPSTLVTSINGFK-CLPLATNKAAIRIMAKNK-PV--QCLVSAKYDNLTVD**RR**S 55

Cs3g04190 -----INPSTLVTSVNGFK-CLPLATNRAAIRIMAKNK-PV--QCLVSAKYYNLTVD**RR**S 55

orange1.1t04775.1 ---------------------------------MAKNK-PV--QCLVSAKYDNLTVD**RR**S 24

orange1.1t04366 ------------------------------------------------------------ 0

BAM29049 -----INPSTLVTSVNGFK-CLPLTTNKAAIRIMAKNK-PL--QCLVSAKYDNLTVD**RR**S 55

Cs2g22150 ------------------------------------------------------------ 0

BAD27258 -------------KVNGFVPPITRVQY----------P-MA--ASTTSIKPVDQTII**RR**S 58

BAD27259 -------------KVNGFVPPITRVQY----------P-MA--ASTTSIKPVDQTII**RR**S 58

Cs3g07850 -------------KVNGFVPPITRVQY----------P-MA--ASTTSIKPVDQTII**RR**S 58

AAM53943 -------------KVNGFVPPITQVQY----------P-MA--ASTSSIKPVDQTII**RR**S 58

Cs7g18530 -------------KVNGFVPPITRVQY----------P-MA--ASTTSIKPVDQNII**RR**S 58

Cs3g07920 -------------KVNGFVPPITRVQY----------H-VA--ASTTPIKPVDQTII**RR**S 58

BAF73933 -------------KVNGFVPPITRVQY----------H-VA--ASTTPIKPVDQTII**RR**S 58

AAM53945 -------------KVNGFVPPITRVQY----------H-VA--ASTTPIKPVDQTII**RR**S 58

BAD27260 -------------KVNGFVPPITRVQY----------P-MA--ASTTXIKPVDQTII**RR**S 58

BAD91045 -------------KVNGFVPPITRVQY----------P-MA--ASTTTIKPVDQTII**RR**S 58

Cs3g07880 -------------NVNGPVPLITRVQY----------R-MA--ASTTTIKPADQSFI**RR**S 58

Cs7g17640 -------------NVNGPVPLITRVQY----------R-MA--ASTTTIKPADQSFI**RR**S 58

Cs2g03570 --------SLNISNSNGFRASPVQ---------------CT--AANATSVCEKAISN**RR**S 62

Cs8g14120.1 -----ISKSNSKSNSNGFCASPIQ---------------CM--AAT-KARDKAINDN**RR**S 66

BAD91046 --------SISKSNSNGFCASPIQ---------------CM--SAT-KVRDKAINDN**RR**S 62

Cs5g15530 DIEIPDHSHVLQNIYAMRNFKLKRIPKEKLHSVPTTLLFSL-EGMPELGWEKLMKLQFQN 208

Cs5g31210 DIEVPYDSPVLQKIYDKRNLKLTRIPKDIMHNVPTTLLHSL-EGMPDLNWQKLLKLQCQD 289

Cs2g06470 GLEIVFPHPIRGAVIDILNQRQQILDSEELVGKNHYPPLLAYLEALPPLYDAHQEQIAKR 209

orange1.1t03278 NLNLPLRSSDINAMLERRHLELNRNYTTGRKEYLAYVSEGI---GKLQDWEMVMKYQRKN 219

XXXXXXX**W**

Cs2g22180 LSSLPKPLVTDFKTSPRQNVLTYEGDRGTKSLEEELQERTRKALR-KSS-----NDPTAT 92

Cs2g07250 DNTVASSPLKHFT-HTTRHPSFFEHDIQVE-HSRKLKDLKHIFSL-VG------GNSFEG 100

BAP75559 DNTVASPPLKHFT-HTTRHPSFFDHDIQVE-HSRKLKEFKHIFSL-VG------GNSFEG 100

Cs2g07240 DSIGVSAPLKHVT-YPCEHRSFNNDDIHID-HAKKLDAVKHILIR-VGD-----DDPFEG 98

BAP75560 DNIGVSARLKRFT-YPSEHSSNFNDDIHIK-HAKKLEVIKHILIK-LGD-----DDSFEG 98

orange1.1t02448 DNIGVSAPLKHFT-YPSEHPSNFNDDIHIK-HAKKLEVIKHILIK-LGD-----DDSFEG 98

orange1.1t03307 DNIGVSARLKGFT-YPSEHPSNFNHDIHIK-HAKKLEVIKHILIK-LGD-----DDSFEG 98

orange1.1t02008 GNFRPTI**W**KDGSISSPVLEV------ETYDKLNEEMKDHVKEMLV-AS---R--NDPVEE 105

Cs4g11980 SNYHPSI**W**GDHFINVSS--NDKYPNA-EVEKRFETLKAEVEKLLM-SNNTAW--KTIEEI 75

orange1.1t04360.1 SNYHPSI**W**GDHFINVSS--NEKYTNT-EVEKRFETLKAEIEKLLV-SNNTAW--KTLEEI 76

orange1.1t04360.2 SNYHPSI**W**GDHFINVSS--NEKYTNT-EVEKRFETLKAEIEKLLV-SNNTAW--KTLEEI 76

Cs4g12080 ASYHPTL**W**GEKFLDHSSVDDSVAMDATIDQDEFEALKQKIKNMLI-SP--TD--K-SFQK 76

Cs4g12050 ASYHPTL**W**GEKFLDCSSADDSVAMDPTIDQDEFEGLKQKIKNMLN-SP--TD--K-SFQK 76

Cs4g12060 ASYHPTL**W**GEKFLDYSSVDDSVAMDPTIDQDEFEALKQKIKNMLI-SP--TD--K-SFQK 76

Cs5g12880 ADFHPSL**W**RNHFLKGAS--DFKTVDHTATQERHEALKEEVRRMIT-DA--ED--K-PVQK 62

Cs5g12900.2 ADFHPSL**W**RNHFLKGAS--DFKTVDHTATQERHEALKEEVRRMIT-DA--ED--K-PVQK 62

AAQ04608 ADFHPSL**W**RNHFLKGAS--DFKTVDHTATQERHEALKEEVRRMIT-DA--ED--K-PVQK 62

Cs4g12350 AGFHPNL**W**GDYFLTLAS--DCKTNDT-THQEEYEALKQEVRSMIT-AT--AD--T-PAQK 71

Cs4g12400 AGFHPNL**W**GDYFLTLAS--DCKTNDT-THQEEYEALKQEVRSMIT-AT--AD--T-PAQK 71

Cs4g12220 -------------------------------------------MA-SD--AD--K-PAQK 11

Cs4g12480 ADFHPTL**W**GNHFLKSAA--DVETIDA-ATQEQHAALKQEVRRMIT-TT--AN--K-LAQK 68

Cs4g11320 ADFHPTL**W**GNHFLKSAA--DVETIDA-ATQEQHAALKQEVRRMIT-TT--AN--K-LAHK 71

orange1.1t03302.1 ADFHPTL**W**GNHFLKSAA--DVETIDA-ATQEQHAALKQEVRRMIT-TT--AN--K-LAQK 74

Q94JS8 ADFHPTL**W**GNHFLKSAA--DVETIDA-ATQEQHAALKQEVRRMIT-TT--AN--K-LAQK 74

Cs4g12450 AGFSPTV**W**GNHFLKSAS--DFKAIDA-TTQELYEALKQEVRMMIT-AT--AD--K-IADK 67

orange1.1t00017.2 AGFHPNL**W**GHHFLKSSF--DFQTIDT-TTQEQYDALKQEVRRMIT-PA--VD--E-ISHK 70

Cs5g06290 ANFHPSV**W**GDRFANYTA--EDKMNHA-RDLKELKALKEEVGRKLL-AT-------AGPIQ 70

Cs3g21560 ANYHPDI**W**GDRFINYNS--EDEIYHG-AQSQEIEELKEEVRRELL-GC--S---ADSLTQ 62

Cs3g21590c ANYHPNI**W**GDRFINYNA--EDEIYHG-AQSQEIEELKEEVRRELL-GS--G---VDSLSR 62

Cs4g12110.1 ANFDPTI**W**GDYFLSYTG--DFKESGD-A-SVKHQELKKEIRTMLR-AD--IN--KPSQTK 76

BAP75561 ANFDPTI**W**GDYFLSYTG--DFKESGD-A-SVKHQELKKEIRTMLR-AD--IN--KPTQTK 76

Cs3g16210 ANFHPSI**W**GDRFLSYTS--DSMEKDD-GS-AKHQELKEEIRRMLK-AY--IN--KPTQNN 136

Cs2g23470 ANFRPSI**W**GDHFLQYTC--DYQETDD--RSLKHLELKKEIRRMLK-AL------NKTSHT 73

Cs2g24110.1 ------------------------------------------------------------ 0

Cs5g23510.1 ANFPPSI**W**GDHFFQYTC--DSQETDD--QNVKHLELKKEIRRMLK-AA------NKTSHT 73

Cs5g23540 ANFPPSI**W**GDHFFQYTC--DSQETDD--QNVKHLELKKEIRRMLK-AV------NKTSHT 73

BAP74389 ANFHPSI**W**GDHFLQYTC--DSQEPDD-GSNVKHLELKKEIRRMLK-AD------NKPSRT 74

Cs4g12090 ANFHPSI**W**GDRFLSYTS--DSMETDD-GSNVKHLELKKEIRRMLK-AD------NKPSRT 75

CuSTS1 ANYHPSI**W**GDHFLQYTC--DTQETDD-GSNVKHLELKKEIRRMLK-AD------NKPSCT 74

Cs4g12120.1 ANYHPSI**W**GDHFLQYTC--DTQETDD-GSNVKHLELKKEIRRMLK-AD------NKPSRT 74

Cs4g12120.2 ANYHPSI**W**GDHFLQYTC--DTQETDD-GSNVKHLELKKEIRRMLK-AD------NKPSRT 74

Cs2g22090 ANYHASI**W**NPELIESFT---TPYTY-ELYANRLDELKQKAKDLFA-SAK-----ESTSEL 72

Cs2g22100.1 ANYHPSI**W**NPELIESFT---TDYTY-ELYAGRLEKLKQEAKELFA-STK------GSYDR 108

Cs2g07280 ANYKPNI**W**KYDFIQSLH---SKYKE-EGYRSRAEKLKNDVKQMFL-EA------ADLLAK 81

Cs2g24530.1 ANYKPNI**W**KYDFIQSLH---SKYKE-EGCRSRAEKLTNDVKQMFL-EA------ADLLAK 63

Cs5g22980 GNYQPSI**W**DHDFLHSFS---CNFTG-ESYKKQAENLKGKVKTMIN-EV---SVTNRPLDQ 108

Cs3g04340 ------------------------------------------------------------ 0

BAD27256 ANYQPSI**W**DHDFLQSLN---SNYTD-ETYKRREEELKGKVMTTIK-DV------TEPLNQ 104

AAM53944 ANYQPSI**W**DHDFLQSLN---SNYTD-EAYKRRAEELRGKVKIAIK-DV------IEPLDQ 104

BAF73932 ANYQPSI**W**DHDFLQSLN---SNYTD-EAYKRRAEELRGKVKIAIK-DV------IEPLDQ 104

AAM53946 ANYQPSI**W**DHDFLQSLN---SNYTD-ETYRRRAEELKGKVKIAIK-DV------TEPLDQ 104

BAD27257 ANYQPSI**W**DHDFLQSLN---SNYTD-ETYKRRAEELKGKVKTAIK-DV------TEPLDQ 104

AOP12358 ANYQPSI**W**DHDFLQSLN---SNYTD-ETYKRRAEELKGKVKTAIK-DV------TEPLDQ 104

Cs3g04170 ANYQPSI**W**DHDFLQSLN---SNYTD-QTYRRRAEELKGKVKTAIK-DV------TEPLDQ 104

Cs3g04360 ANYQPSI**W**DHDFLQSLN---SNYTD-QTYRRRAEELKGKVKTAIK-DV------TEPLDQ 104

Cs3g04190 ANYQPSI**W**DHDFLQSLN---SNYT------------------------------------ 76

orange1.1t04775.1 ANYQPSI**W**DHDFLQSLN---SKYTD-EAYKRRAEELKGKVKIAIK-DV------IEPLDQ 73

orange1.1t04366 -----------MAKNKP---VQCLD-EAYKRRAEELKGKVKIAIK-DV------IEPLDQ 38

BAM29049 ANYQPSI**W**DHDFLQSLN---SKYTD-EAYKRRAEGLKGKVKIAIK-DV------IEPLDQ 104

Cs2g22150 --------------------------------------------------------MLPI 4

BAD27258 ADYGPTI**W**SFDYIQSLD---SKYKG-ESYARQLEKLKEQVSAMLQ-QDNKVVD-LDPLHQ 112

BAD27259 ADYGPTI**W**SFDYIQSLD---SKYKG-ESYARQLEKLKEQVSAMLQ-QDNKVVD-LDPLHQ 112

Cs3g07850 ADYGPTI**W**SFDYIQSLD---SKYKG-ESYARQLEKLKEQVSAMLQ-QDNKVVD-LDPLHQ 112

AAM53943 ADYGPTI**W**SFDYIQSLD---SKYKG-ESYARQLEKLKEQVSAMLQ-QDNKVVD-LDPLHQ 112

Cs7g18530 ADYGPTI**W**SFDYIQSLD---SKYKG-ESYARQLEKLKEQVSAMLQ-QDNKVVD-LDPLHQ 112

Cs3g07920 ADYGPTI**W**SFDYIQSLD---SKYKG-ESYARQLEKLKEQVSAMLQ-QDDKVVD-LDPLHQ 112

BAF73933 ADYGPTI**W**SFDYIQSLD---SKYKG-ESYARQSEKLKEQVSAMLQ-QDDKVVD-LDPLHQ 112

AAM53945 ADYGPTI**W**SFDYIQSLD---SKYKG-ESYARQLEKLKEQVSAMLQ-QDNKVVD-LDTLHQ 112

BAD27260 ADYGPTI**W**SFDYIQSLD---SKYKG-ESYARQLEKLKEQVSAMLQ-QDNKVVD-LDPLHQ 112

BAD91045 ADYGPTI**W**SVDYIQSLD---SEYKE-KSYARQLQKLKEQVSAMLQ-QDNKVVD-LDPLHQ 112

Cs3g07880 ADYGPTI**W**SFDYIQSLD---SKYNG-ESYARQLEKLKEQVRAMLQ-GGDKVVD-LDPLHQ 112

Cs7g17640 ADYGPTI**W**SFDYIQSLD---SKYNG-ESYARQLEKLKEQVRAMLQ-RDDKVVD-LDPLHQ 112

Cs2g03570 ANYQPSM**W**GYDYLQSLS---NEYVV-ESYAQRIEKLKGEVRLMLE-N--KEVDYVDALHQ 115

Cs8g14120.1 ANYQPSI**W**SYDYLQSLS---NGYAG-ESCAQRIEKLKGEVRLMLD-NYKELDDYVDALYY 121

BAD91046 ANYQPSM**W**SYDYLQSLS---NGYVG-ESCAQRIEKLKGEVRLMLD-NYKEVDDYVDALHC 117

Cs5g15530 ----GSFL-----FSLSSTAYAFMQT-QDENCLKYLTQVVRRFNG-GVPSSYPM-DLFER 256

Cs5g31210 ----GSFL-----FSPSSTAYALMQT-KDENCLKYLTKAVQRFNG-GVPNVYPV-DIFEH 337

Cs2g06470 LSFDGSLF-----HSPSATARAYMAT-GNDKCLAYLQSLVQTCADDGVPPLYPIDEDLMK 263

orange1.1t03278 ----GSLF-----NSPSTTAAALTHF-HNAGCLHYLSSLLEKFGN-AVPTVHPL-DIYIN 267

**D**X**DD**

Cs2g22180 MKLIDTIQRLGIGYHFEDEIMERLERFSDG---DA--------AGEENLFENALRFRLLR 141

Cs2g07250 LVMIDAVQRLGIEYLFKDEIEEILQRQYIISSTCG--------GHLHDLQEVALRFRLLR 152

BAP75559 LVMIDAVQRLRIEYLFKDEIEEILQRQYIISSTCG--------GHLHDLQEVALRFRLLR 152

Cs2g07240 LAMIDTVQRLGIDYYFQDEIEQILQRQSIVFSNHG--------DRHNDLQEVALRFRLLR 150

BAP75560 LAMIDVVQRLGIDYYFQDEIELILRRQYSIFFTDG--------DRYNDLQEVALRFRLLR 150

orange1.1t02448 LAMIDVVQRLGIDYYFQDEIELILRRQYSIFFTDG--------DRYNDLQEVALRFRLLR 150

orange1.1t03307 LAMIDVVQRLGIDYYFQDEIELILRRQYSIFFTDG--------DRYNDLQEVALRFRLLR 150

orange1.1t02008 VVLINLLCRLGVSYHFENEIEERLNHIFEMQPDLA---AE----KDCDLYATAILFRVFR 158

Cs4g11980 VTLVDQLQRLGVAYHFENEIKEDLQSIYNSHVNSN---CDVNYDHNNDLYTVALRFRLLR 132

orange1.1t04360.1 VAIVNQLQRLGVAYHFENEIKEALQTIYDSHVNGN---CDVNYDHNNDLYIVALRFRLLR 133

orange1.1t04360.2 VAIVNQLQRLGVAYHFENEIKEALQTIYDSHVNGN---CDVNYDHNNDLYIVALRFRLLR 133

Cs4g12080 LSLIDAVQRLGVAYHFEREIEDELEKLSHDEY------------DGNDVHTVALRFRLLR 124

Cs4g12050 LSLIDAVQRLGVAYHFEREIEDELEKLSHDEY------------DGNDVHTVALRFRLLR 124

Cs4g12060 LSLIDAVQRLGVAYHFEREIEDELEKLSPDEY------------DGNDVHSVALRFRLLR 124

Cs5g12880 LRLIDEVQRLGVAYHFEKEIEDAIQKLCPNYIH-------S---NSPDLHTVSLHFRLLR 112

Cs5g12900.2 LRLIDEVQRLGVAYHFEKEIEDAIQKLCPIYID-------S---NRADLHTVSLHFRLLR 112

AAQ04608 LRLIDEVQRLGVAYHFEKEIGDAIQKLCPIYID-------S---NRADLHTVSLHFRLLR 112

Cs4g12350 LQLVDAVQRLGVAYHFEQEIEDAMEKIYHDDFD--------N-IDDVDLYTVSLRFRLLR 122

Cs4g12400 LQLVDAVQRLGVGYHFEQEIEDALENIFHNSFD--------N-NDDVDLHTVSLRFRLLR 122

Cs4g12220 LCLIDVVQRLGVAYHFIKEIDDALEKISHDPFD-----------DKDDIYIVSLCFRLLR 60

Cs4g12480 LHMIDAVQRLGVAYHFEKEIEDELGKVSHDL-------------DSDDLYVVSLRFRLFR 115

Cs4g11320 LHMIDSVQRLGVAYHFEKQIEDELGKLSHDL-------------DSDDLYVVSLRFRLFR 118

orange1.1t03302.1 LHMIDAVQRLGVAYHFEKEIEDELGKVSHDL-------------DSDDLYVVSLRFRLFR 121

Q94JS8 LHMIDAVQRLGVAYHFEKEIEDELGKVSHDL-------------DSDDLYVVSLRFRLFR 121

Cs4g12450 LHLIDAVQRLGVAYHFEKEIEDELEKILDHLDN-------DN-IGGDDFYTLSLSFRLLR 119

orange1.1t00017.2 LHLIDAVQRLGVAYQFEKEIEDELQKLANDLGS-----------DSDNLYTVSLRFRLLR 119

Cs5g06290 LNLIDAIQRLGVGYHFERELEQALQHLYNEKYSD-----D---DTEDDLYRISLRFRLLR 122

Cs3g21560 LKLIDAILRLGVGYQFERELEEALQNLCDAYSNNF---YN---IEDDDPYHVALRFRLLR 116

Cs3g21590c LKLIDAILRLGVGYQFERELEEALQNLYDAFSNHF---YN---IEDNDLYHVALRFRILR 116

Cs4g12110.1 LDLIDDIQRLGVSYHFESEIDEILQKMHEANQDCD---LGDD-ENVQELYYISLQFRLLR 132

BAP75561 LDLIDDIQRLGVSYHFESEIDEILRKMHEANQDCD---LGDD-ENVQELYYISLHFRLLR 132

Cs3g16210 LDLIDAIQRLGVSYHFESEIDEILGKLHEAHQDCG---LGDN-EN-DELYYISLQFRLLR 191

Cs2g23470 LELIDAIQRLGVSYHFESEIDEILGKMHEAYRECD---LWDN-EN-DKLYYISLQFRLFR 128

Cs2g24110.1 --------------------------MHKAYRDGD---LWDN-EN-DKLYYIALQFRLFR 29

Cs5g23510.1 LELIDAIQRLGVSYHFENEIDEILGKMHKTYRDCD---LCDN-EN-DGLYYISLQFRLFR 128

Cs5g23540 LELIDAIQRLGVSYHFESEIDEILGKMHKTYRDCD---LCDN-EN-DELYYISLQFRLFR 128

BAP74389 LELIDAIQRLGVSYHFESEIDEILGRVHQAYQESD---LCVN-EN-DGLYYISLQFRLLR 129

Cs4g12090 LQLIDAIQRLGVSYHFESEIDEILGKMHKASQDSD---LCDN-EN-DELYYISLHFRLLR 130

CuSTS1 LQLIDAIQRLGVSYHFESEIDEILGKMHKAYQDSD---LCDN-EN-DELYYISLHFRLLR 129

Cs4g12120.1 LQLIDAIQRLGVSYHFEN------------------------------------------ 92

Cs4g12120.2 LQLIDAIQRLGVSYHFESEIDEILGKMHKASQDSD---LCDN-EN-DELYYISLHFRLLR 129

Cs2g22090 LKLTDSVQKLGVAYHFEEEIKEAMNILKGD-A------------TIKDLNATSLHFRLLR 119

Cs2g22100.1 LKLIDSLQRLGVAYHFEQEIEEAVNLLTKDDN------------TIKDLNETAFHFRILR 156

Cs2g07280 LELIDRICKLGLSYLFEEKIREVLVDTVAFLKND------TGCLQVKDLYATALCFKLLR 135

Cs2g24530.1 LELIDRICKLGLSYLFEEQIREILVDTVAFLKND------TGCLEVKDLYATALCFKLLR 117

Cs5g22980 LELIENLQRLGLAYHFETEIKNILHNIYNNK-DD--------KWKNENLYATSLEFRLLR 159

Cs3g04340 ----MYLQRLGLAYHFETEIRKILHNIYNSNKDY--------NWRKENLYATSLEFRLLR 48

BAD27256 LELIDSLQRLGLAYHFETEIRNILHDIYNSNNDY--------VWRKENLYATSLEFRLLR 156

AAM53944 LELIDNLQRLGLAHRFETEIRNILNNIYNNNKDY--------NWRKENLYATSLEFRLLR 156

BAF73932 LELIDNLQRLGLAHRFETEIRNILNNIYNNNKDY--------NWRKENLYATSLEFRLLR 156

AAM53946 LELIDNLQRLGLAYRFETEIRNILHNIYNNNKDY--------VWRKENLYATSLEFRLLR 156

BAD27257 LELIDNLQRLGLAYHFEPEIRNILRNIHNHNKDY--------NWRKENLYATSLEFRLLR 156

AOP12358 LELIDNLQRLGLAYHFEPEIRNILRNIHNHNKDY--------NWRKENLYATSLEFRLLR 156

Cs3g04170 LELIDNLQRLGLAYHFETEIRNILHNIYNNNKDY--------IWRKANLYATSLEFRLLR 156

Cs3g04360 LELIDNLQRLGLAYHFEPEIRNILRNIHNHNKDY--------NWRKENLYATSLEFRLLR 156

Cs3g04190 --------------------------------------------RKENLYATSLEFRLLR 92

orange1.1t04775.1 LELIDNLQRLGLAHRFETEIRNILNNIYNNNKDY--------NWRKENLYATSLEFRLLR 125

orange1.1t04366 LELIDNLQRLGLAHRFETEIRNILNNIYNNNKDY--------NWRKENLYATSLEFRLLR 90

BAM29049 LELIDNLQRLGLAHRFETEIRNILNNIYNNNKDY--------NWRKENLYATSLEFRLLR 156

Cs2g22150 LELIDDVQRLGLGYRFENEIKRALHRILSWQGYD-------HVNPEKDLHVTALRFRLLR 57

BAD27258 LELIDNLHRLGVSYHFEDEIKRTLDRIHNKNL-------------MKIYMPQALKFRILR 159

BAD27259 LELIDNLHRLGVSYHFEDEIKRTLDRIHNKNT-------------NENLYATALKFRILR 159

Cs3g07850 LELIDNLHRLGVSYHFEDEIKRTLDRIHNKNT-------------NKSLYARALKFRILR 159

AAM53943 LELIDNLHRLGVSYHFEDEIKRTLDRIHNKNT-------------NKSLYARALKFRILR 159

Cs7g18530 LELIDNLHKLGVSYHFEDEKKRTLDRIHTKNT-------------NKSLYATALKFRILR 159

Cs3g07920 LDLIDNLHRLGVSYHFEDEIKRTLDRIHNKNT-------------NKSLYATALKFRILR 159

BAF73933 LELIDNLHRLGVSYHFEDEIKRTLDRIHNKNT-------------NKSLYATALKFRILR 159

AAM53945 LELIDNLHRLGVSYHFEDEIKRTLDRIHNKNT-------------NKSLYATALKFRILR 159

BAD27260 LELIDNLHRLGVSYHFEDEIKRTLDRIHNKNT-------------NENLYATALKFRILR 159

BAD91045 LELIDNLHRLGVSYHFEDEIKRTLDRIHNKNT-------------NKSLYARALKFRILR 159

Cs3g07880 LELIDNLHRLGVSYHFEDEIKRTLERICNKNT-------------EKSLYTVALKFRILR 159

Cs7g17640 LELIDNLHRLGVSYHFEDEIKRTLERICNKNT-------------EKSLYNVALKFRILR 159

Cs2g03570 LEIVDNLQRLGVSYHFEDEIKRFLSRIYNERNSR--SSYHAKEKQESSLYAVALEFRLLR 173

Cs8g14120.1 LEIVDNLQRLGVSYHFEGEIKRFLNRIYNKRNSRRSSTYHAKENQESLLYAASLEFRLLR 181

BAD91046 LEIVDNLQRLGVSYHFEGEIKRFLNSIYNKRNSRRSSTYHAKENQESLLYAASLEFRLLR 177

Cs5g15530 LWAVDRLQRLGISRYFRSEIKECMDYIYRYWNEKGI-FSGRN-TRIP**D**I**DD**TSMGFRLLR 314

Cs5g31210 LWAVDRLQRLGISRYFQPEIKECLDYVYRYWTEEGI-CWARN-TRVQ**D**L**DD**TSMGFRLLR 395

Cs2g06470 LCIVNQLQRLGLAEHFLQEIKDVLAQVYRNYKNEELSANPIN-SEPTQLYKDSLAFQLLR 322

orange1.1t03278 LTMVESLESLGVDRHFRTEIKRVLDETYRFWLQG------EE-EIFLDLTTCAMAFRLLR 320

Cs2g22180 HNGLPACT-DIFKKFINKE------------GKLKE---SVSKDTRGMLSLYEASYLATQ 185

Cs2g07250 QEGYYVPA-DMFNNFMIKE------------GRFSQF--NVSEDIGRLMEVYEASQLSIA 197

BAP75559 QEGYYVPA-DMFNNFRIKE------------GRFSRI--NVSEDIGTLMEVYEASQLSIA 197

Cs2g07240 QQGYYVSP-DVFNNFRNKE------------GKSKQ---NISGDINGLMSLYEASQLSIV 194

BAP75560 QQGYYVSA-DVFNRFRNKE------------GEFKQ---NISEDINGLMSLYEASQLSIG 194

orange1.1t02448 QQGYYVSP-DVFNRFRNKE------------GEFKQ---NVSEDINGLMSLYEASQLSIG 194

orange1.1t03307 QQGYYVSP-DVFNRFRNKE------------GEFKQ---NVSEDINGLMSLYEASQLSIG 194

orange1.1t02008 QHGFNVSS-GVFNKFKDHD------------GNFKE---SLTSDARGMLSLYEATHLRVH 202

Cs4g11980 QHGYKVSA-DIFKKFKDEK------------GEFKA---MLTNDARGLLCLYEASYLRVQ 176

orange1.1t04360.1 QHGYKVSA-DIFKKFRDEK------------GEFKA---MLTNDAKGLLCLYEASYLRVQ 177

orange1.1t04360.2 QHGYKVSA-DIFKKFRDEK------------GEFKA---MLTNDAKGLLCLYEASYLRVQ 177

Cs4g12080 QQGYRISC-DIFGGFKDDR------------GKFKV---SLINDLTGMLSLYEAAHLRIR 168

Cs4g12050 QQGYRISC-DIFSGFKDDQ------------GKFKV---SLINDVTGMLSLYEAAHLRIH 168

Cs4g12060 QQGYRISC-DIFGGFKDDR------------GKFKV---SLINDVTGMLNLYEAAHLRIR 168

Cs5g12880 QQGIKISC-DVFEKFKDDE------------GRFKS---SLINDVQGMLSLYEAAYMAVR 156

Cs5g12900.2 QQGIKISC-DVFEKFKDDE------------GRFKS---SLINDVQGMLSLYEAAYMAVR 156

AAQ04608 QQGIKISC-DVFEKFKDDE------------GRFKS---SLINDVQGMLSLYEAAYMAVR 156

Cs4g12350 QQGFKVPC-DVFEKFKDDE------------GKFKA---SLVKDVQGILSLYEAGHLAIR 166

Cs4g12400 QQGFKVSC-DVFEKFKDDE------------GKFKA---SLVKEVQGILSLYEAGHLAIR 166

Cs4g12220 QHGIKMSC-DVFEKFKDDD------------GKFKA---SLTNDVEGMLSLYEVAHLAIP 104

Cs4g12480 QQGVKISC-DVFEKFKDDE------------GKFKE---SLINDIRGMFSLHEAAYLAVR 159

Cs4g11320 QQGVKISC-DVFEKFKDDE----------------------------------------- 136

orange1.1t03302.1 QQGVKISC-DVFDKFKDDE------------GKFKE---SLINDIRGMLSLYEAAYLAIR 165

Q94JS8 QQGVKISC-DVFDKFKDDE------------GKFKE---SLINDIRGMLSLYEAAYLAIR 165

Cs4g12450 QQGVKISC-DVFEKFKNNE------------GKFKA---SMINDVQVMLSLYEAAHLAIN 163

orange1.1t00017.2 QQRVKISC-DVFEKFKDDE------------GKFKA---SMINNVRGMLSLYEAAHLAVH 163

Cs5g06290 QHGYNVSC-DKFNMFKDDK------------GNFKE---SLASDALGMLSLYEAAHLGVH 166

Cs3g21560 QRGYKVSC-DIFNQFKDGK------------GNFKE---SLKTDVSGMLSLYEAAHLGVH 160

Cs3g21590c QRGYKVSC-DIFNKFKDGK------------GNFKE---SLKIDVSGMLSLYEAAHLGVH 160

Cs4g12110.1 QNGYKISA-DVFNSFKDSN------------GNFKS---FLKRDIRGMLSLYEAAHLRVH 176

BAP75561 QNGYKISA-DVFNSFKDSN------------GNFKS---FLKRDIRGMLSLYEAAHLRVH 176

Cs3g16210 QHGCKISA-DVFKRFKDSD------------GNFKT---SLAKDVGGMLSLYEATHLGVH 235

Cs2g23470 QNGYRISS-DVFNTFKGSD------------GKFTA---SLAKDVRGMLSLYEATHLRVH 172

Cs2g24110.1 QNGYRISA-DVFNTFKGSD------------GKFKA---SLAKDVRGMLSLCEATHLRVH 73

Cs5g23510.1 QNGYRISA-DVFNAFMGSD------------GKFMA---SLAKDVRGMLSFYEATHLRVH 172

Cs5g23540 QNGYRISA-DVFNTFEGSD------------GKFKA---SLAKDVRGMLSLYEATHLRVH 172

BAP74389 ENGYRISA-DVFNKFRDID------------GNFKP---SLAKDVRGMLSLYEATHLRVH 173

Cs4g12090 QNGYKISA-DVFKKFKDTD------------GNFKT---SLAKDVRGMLSLYEATHLGVH 174

CuSTS1 QNGYKISA-DVFKKFKDRD------------GNFKT---SLAKDVRGMLSLYEATHLGVH 173

Cs4g12120.1 ----------VFKKFKDTD------------GNFKT---SLAKDVRGMLSLYEATHLGVH 127

Cs4g12120.2 QNGYKISA-DVFKKFKDTD------------GNFKT---SLAKDVRGMLSLYEATHLGVH 173

Cs2g22090 EHGHPVS---VFDKFRKRD------------GRFHD---NLREDIEGLLNLFEASFLGIE 161

Cs2g22100.1 EHGHSIST-EVFDKFKNVD------------GRFDE---SLEADITGLLSLFEASFLGSV 200

Cs2g07280 QHGYEISQ-DVFLDFMDET------------GTTFST--SKCTDIKGLIELCEASQLALE 180

Cs2g24530.1 QHGYEISQ-DMFIDFMDET------------GTTFST--SKCTDIKGLIELFEASHLALE 162

Cs5g22980 QHGYNVSQ-EVFSSFRDKN------------GGFI------CNDFKGILRLYEASYCSLE 200

Cs3g04340 QHGYPVSQ-DVFNGFKDDK------------GGFI------CNDFKEIMSLHEASYYSFE 89

BAD27256 QHGYPVSQ-EVFNGFKDDQ------------GGFI------CDDFKGVLSLHEASYFSLE 197

AAM53944 QHGYPVSQ-EVFNGFKDDQ------------GGFI------CDDFKGILSLHEASYYSLE 197

BAF73932 QHGYPVSQ-EVFNGFKDDQ------------GGFI------CDDFKGILSLHEASYYSLE 197

AAM53946 QHGYPVSQ-EVFNGFKDDQ------------GGFI------FDDFKGILSLHEASYYSLE 197

BAD27257 QHGYPVSQ-EVFSGFKDDK------------VGFI------CDDFKGILSLHEASYYSLE 197

AOP12358 QHGYPVSQ-EVFSGFKDDK------------VGFI------CDDFKGILSLHEASYYSLE 197

Cs3g04170 QHGYPVSQ-EVFSGFKDDK------------GGFI------CDDFKGILSLHEASYYSLE 197

Cs3g04360 QHGYPVSQ-EVFSGFKDDK------------GGFI------CDDFKGILSLHEASYYSLE 197

Cs3g04190 QHGYPVSQ-EVFNGFKDDQ------------GGFI------YDDFKGILSLHEASYYSLE 133

orange1.1t04775.1 QHGYPVSQ-EVFNGLKDGQ------------GGFI------CDDFKGILSLHEASYYSLE 166

orange1.1t04366 QHGYPVSQ-EVFNGLKDGQ------------GGFI------CDDFKGILSLHEASYYSLE 131

BAM29049 QHGYPVSQ-EVFNGLKDGQ------------GGFI------CDDFKGILSLHEASYYSLE 197

Cs2g22150 QHGFDISQGNNLQYCFEVQRSLRHSWTLKPEGNFTE---TVHKDVKGVLSLNEASYLAFE 114

BAD27258 QYGYNTPVKETFSHFMDEK------------GSFKSS--SHSDDCKGMLALYEAAYLLVE 205

BAD27259 QYGYNTPVKETFSHFMDEK------------GSFKSS--SHSDDCKGMLALYEAAYLLVE 205

Cs3g07850 QYGYENPVKESFSRFMDEK------------GSFKLS--SHSDECKGMLALYEAAYLLVE 205

AAM53943 QYGYKTPVKETFSRFMDEK------------GSFKLS--SHSDECKGMLALYEAAYLLVE 205

Cs7g18530 QYGYNTPVKETFSRFMDEK------------ESFKLS--SHSDECKGMLALHEAAYLLVE 205

Cs3g07920 QYGYNTPVKETFSRFMDEK------------GIFKLS--SHSDDCKGMLALYEAAYLLVE 205

BAF73933 QHGYNTPVKETFSRFMDEK------------GIFKLS--SHSDDCKGMLALYEAAYLLVE 205

AAM53945 QYGYNTPVKETFSRFMDEK------------GSFKSS--SHSDDCKGMLALYEAAYLLVE 205

BAD27260 QYGYNTPVKETFSRFMDEK------------GSFKSS--SHGDDCKGMLALYEAAYLLVE 205

BAD91045 QYGYNTPVKETFSRFMDEK------------GSFKLS--SHSDDCKGMLALYEAAYLLVE 205

Cs3g07880 QYGYDTPVKETFSCFMDEK------------GSFKSS--SHGDDCKGMLALYEAAYLLVE 205

Cs7g17640 QYGYDTSVKETFSCFMDEK------------GSFKSS--SHGDDCKGMLALYEAAYLLVE 205

Cs2g03570 QHGFDMHAQGTLSLFMDEK------------GKLKS---CLGDDIKGILALYEAAYLLVE 218

Cs8g14120.1 QHGYDIHAHGTLSSFMDEK------------GKFKS---CLGDDIKGILALYEAAYLLGE 226

BAD91046 QHGYDIHAHGTLSSFMDEK------------GKFKS---CLGDDIKGILALYEAAYLLGE 222

Cs5g15530 LHGYDVSA-DVLQYFEEGG-------------EFFCFLGETSQAITATFNLYRASQVQFP 360

Cs5g31210 LHGYDVSA-EAFEHFEKGG-------------EFFCFVGQSTQAVTGVYNLYRASQVLFP 441

Cs2g06470 MHGYSVSP-WTFCWFLNNP------------EILDEIEKNPEYFSSVMLNVYRATDLMFA 369

orange1.1t03278 LNGYDVSS-DPLTQFAEDN-------------QFFNSLKGHLKDIRAVLELYRASQITIY 366

:

Cs2g22180 DK-DILFEAMEFTKTELTQSVSLM-------D-SQSSRHVVQALALPRHLR-MSRSEAGN 235

Cs2g07250 GE-EGLDEAGQFSAKMLNECMTHL-------D-HNQALAIGNTLRHPYHKS-LPRFMAKD 247

BAP75559 GE-EGLDEAGHFSAKMLNECMTHL-------D-HYHALAIGNTLRHPYHKS-LPRFMAKD 247

Cs2g07240 GE-DVLDEAGDFSVNLLSEYATHL-------ADYDLAGLVKHLLLHPYRKS-LS--SARN 243

BAP75560 GE-DGLDEAGHFSATHL--------------ANYDLAGVVEHLLLYPYRKS-LS--PAKN 236

orange1.1t02448 GE-DVLDEAGHFSATHL--------------ANYDLAGVVEHLLLHPYRKS-LS--PAKN 236

orange1.1t03307 GE-DVLDEAGHFSATHL--------------ANYDLAGVVEHLLLHPYRKS-LS--PAKN 236

orange1.1t02008 GE-DILEEALTFTTAHLKSMAAP-----N--LDQNLAKHINDALEQPLHMG-VPRIEAHK 253

Cs4g11980 GE-NILEEACEFSRKHLKSLLPH--------ISTSLANQVEHSLEIPLHRG-MPRLEARQ 226

orange1.1t04360.1 GE-NILEEACEFSRKHLKSLLSH--------LSTPLADQVEHSLEIPLHRG-MPRLEARQ 227

orange1.1t04360.2 GE-NILEEACEFSRKHLKSLLSH--------LSTPLADQVEHSLEIPLHRG-MPRLEARQ 227

Cs4g12080 GE-DILDEAVAFTTSHLESMVTQ--------VSPQLSDEILHALNRPIRRG-LPRLEAIY 218

Cs4g12050 GE-DILDDALALTTSHLESMVTQ--------VSPQLSDEILHALNRPIRRG-LPRLEAIY 218

Cs4g12060 GE-DILDEALAFTTSHLESMVTQ--------VSPQLSDEILHALNRPIRRG-LPRLEAVY 218

Cs5g12880 GE-HILDEAIAFTTTHLKSLVAQ----DH--VTPKLAEQINHALYRPLRKT-LPRLEARY 208

Cs5g12900.2 GE-HILDEAIAFTTTHLKSLVAQ----DH--VTPKLAEQINHALYRPLRKT-LPRLEARY 208

AAQ04608 GE-HILDEAIAFTTTHLKSLVAQ----DH--VTPKLAEQINHALYRPLRKT-LPRLEARY 208

Cs4g12350 GE-DILDEAIAFTRTHLKSMASD----DV--CPNNLAEQINHALDCPLRRA-FPRVETRF 218

Cs4g12400 GE-DILDEAIAFTRTHLQSMVSH----DV--RPNNLAEQINHALDCPLRRA-LPRVETRF 218

Cs4g12220 GE-DILDEAIVFTTTHLKSMVTN----SQ--VNSTFAEQIRHSLRVPLRRA-VSRLESRY 156

Cs4g12480 GE-DILDEAIAFTTSHLKSIISISDH-SH--VNSKLPEQIRRSLQIPLRKA-AARLEARY 214

Cs4g11320 ----------------------------------------------------AARLEARY 144

orange1.1t03302.1 GE-DILDEAIVFTTTHLKSVISISDH-SH--VNSNLAEQIRHSLQIPLRKA-AARLEARY 220

Q94JS8 GE-DILDEAIVFTTTHLKSVISISDH-SH--ANSNLAEQIRHSLQIPLRKA-AARLEARY 220

Cs4g12450 GE-DILDEAIVFTTTHLKSMVSH--------ASPNLAEQINHALKLPLRKA-LPRLEARY 213

orange1.1t00017.2 GE-VILDEAIVFTTTHLKSMISR-----V--ISNNLAEQIQHALRLPLRKA-LPRLEARY 214

Cs5g06290 GE-DILDEAIAFTTTHLKSVATH--------LSNPLKAQVRHALRQPLHRG-LPRLEHRR 216

Cs3g21560 GE-DILDEAIAFTTAHLQSSM---------------APQVIHALRQPLHKG-MPRLEARS 203

Cs3g21590c GE-DILDEAIAFTTAHLQSSM---------------APQVIHALRQPLYKG-LPRIEARF 203

Cs4g12110.1 GE-NILNEALTFTVTHLESFTS------Q--SNTQLAAQVNRALNRPIRKS-LPRLEAKH 226

BAP75561 GE-NILNEALTFTVTHLESFTS------Q--SNTQLAAQVNRALNRPIRKS-LPRLEAKH 226

Cs3g16210 EE-NILDEALAFTTSHLESIAAH-----Q--IGSPLAERVKHALIQPIHRG-LQRLEARH 286

Cs2g23470 EE-NILDEALAFTTSHLESIATH-----Q--IRSPLAEQVKHALVQPIHKG-LQRLEARE 223

Cs2g24110.1 EE-NILDEALAFTTSHLESIATH-----Q--IRSPLAEQVKHALVQPIHKG-LQRLEARQ 124

Cs5g23510.1 EE-NILDEALAFTSSHLESVAK------Q--VSSPLAEQVKHALVQPIHKG-LERLEARH 222

Cs5g23540 GE-NILDEALAFTTSHLESVAK------Q--VCSPLAEQVKHALVQPIHKG-LERLEARH 222

BAP74389 EE-NILDEAHAFATSHLESIATH-----Q--ISSPLAEQVKHALFQPIHKG-VQRLEARH 224

Cs4g12090 EE-DILDEALAFTTSHLESIATH-----Q--IRSPLVEQVKHALVQPIHRG-LQRLEARQ 225

CuSTS1 EE-DILDEALAFTTSHLESIATH-----Q--IRSPLVEQVKHALVQPIHRG-LQRLEARQ 224

Cs4g12120.1 EE-DILDEALAFTTSHLESIATH-----Q--IRSPLVEQVKHALVQPIHRG-LQRLEARQ 178

Cs4g12120.2 EE-DILDEALAFTTSHLESIATH-----Q--IRSPLVEQVKHALVQPIHRG-LQRLEARQ 224

Cs2g22090 G-EDVLEEANIFCTEHLKESLGTL------GSKIILAEQVQQSLDIPSYWR-MPRIEAQN 213

Cs2g22100.1 EEEDVLEEAKSFSTKHLNDYLLGK------LETNILTEKLQQSLHIPLYWR-MQRNEAQN 253

Cs2g07280 GE-SILDEAKVFSGGTLKGIYSS--------LNTDLAQTVARVLELPSHWR-VPWYEVRW 230

Cs2g24530.1 GE-SILDEAKVFSGGTLKGIYSS--------LNTDLAQTVARVLELPSHWR-VPWYEVRW 212

Cs5g22980 GE-SIMEEAWQFTSKHLKQCLNSN----K--DDEDLNEQARRALELPLHWR-MPRLEARW 252

Cs3g04340 GE-SIMEEAWQFTSKHLKEVMISK-S-KQ--GDVFVAEQAKRALELPLHWK-VPMLEARQ 143

BAD27256 GE-SIMEEAWQFTSKHLKEVMISK-S-KQ--GDVFVAEQAKRGLELPLHWK-VPMLEARW 251

AAM53944 GE-SIMEEAWQFTSKHLKEVMISK-N-ME--EDVFVAEQAKRALELPLHWK-VPMLEARW 251

BAF73932 GE-SIMEEAWQFTSKHLKEVMISK-N-ME--EDVFVAEQAKRALELPLHWK-VPMLEARW 251

AAM53946 GE-SIMEEAWQFTSKHLKEVMISK-S-ME--EDVFVAEQAKRALELPLHWK-VPMLEARW 251

BAD27257 GE-SIMEEAWQFTSKHLKEMMITSNS-KE--EDVFVAEQAKRALELPLHWKKVPMLEARW 253

AOP12358 GE-SIMEEAWQFTSKHLKEMMITSNS-KE--EDVFVAEQAKRALELPLHWK-APMLEARW 252

Cs3g04170 GE-SIMEEAWQFTSKHLKETMIISNS-KE--EYVFVAEQAKRALELPLHWK-VPTLEARW 252

Cs3g04360 GE-SIMEEAWQFTSKHLKETMIISNS-KE--EYVFVAEQAKRALELPLHWK-VPMLEARW 252

Cs3g04190 GE-SIMEEAWQFTK------------------------QAKRAVELPLHWK-VPMLEARW 167

orange1.1t04775.1 GE-SIMEEAWQFTSKHLKEAMISK-S-KE--EHVFVAEQAKRTLELPLHWN-VPMLEARW 220

orange1.1t04366 GE-SIMEEAWQFTSKHLKEVMISK-S-KE--EHVFVAEQAKRTLELPLHWN-VPMLEARW 185

BAM29049 GE-SIMEEAWQFTSKHLKEVMISK-S-KE--EHVFVAEQAKRALELPLHWK-VPMLEARW 251

Cs2g22150 GE-DILNEALTFSRTHLNQLKTK--------LNPYMSELVSHSLELPRHYR-MRRLEVRW 164

BAD27258 EESSIFRDAIRFTTAYLKEWVVKHDI-DK-NDDEYLCTLVKHALELPLHWR-MRRLEARW 262

BAD27259 EESSIFRDAIRFTTAYLKEWVVKHDI-DK-NDDEYLCTLVKHALELPLHWR-MRRLEARW 262

Cs3g07850 EESSIFRDAIRFTTAYLKEWVAKHDI-DK-NDDEYLCTLVKHALELPLHWR-MRRLEA-- 260

AAM53943 EESSIFRDAIRFTTAYLKEWVAKHDI-DK-NDNEYLCTLVKHALELPLHWR-MRRLEARW 262

Cs7g18530 EESSIFRDAKSFTTAYLKEWVIKHDN-NK-HDDEHLCTLVNHALELPLHWR-MPRLEARW 262

Cs3g07920 EESSIFRDATSFTTAYLKEWVIKHDN-IK-HDDEHLCTLVNHALELPLHWR-MPRLEA-- 260

BAF73933 EESSIFRDATSFTTAYLKEWVIKHDN-IK-HDDEHLCTLVNHALELPLHWR-MPRLEARW 262

AAM53945 EESSIFRDAKSFTTAYLKEWVIEHDN-NK-HDDEHLCTLVNHALELPLHWR-MPRLEARW 262

BAD27260 EESSIFRDAKSFTTAYLKEWVIKHDN-NK-HDDEHLCTLVNHALELPLHWR-MPRLEARW 262

BAD91045 EESSIFRDAIRFTTAYLKEWVVKHDI-DK-NDDEYLCTLVKHALELPLHWR-MRRLEARW 262

Cs3g07880 GESSIFRDAISFTTAYLKEWAAKHDN-NK-HHNEYLCTLVNHALELPLHWR-TRRLEARW 262

Cs7g17640 GESSIFRDAISFTTAYLKEWAAKHDN-NK-HHNEYLCTLVNHALELPLHWR-TRRLEARW 262

Cs2g03570 EESNIFNEAINFTTTHLKEYVKHNNN----DDDGYLSTLVEHALELPLHWR-MVRLEARW 273

Cs8g14120.1 EESTIFHEAINFTTTHLEEYVKKHND-DDDDDGGYFSALVKHALELPLHWR-MVRLEARW 284

BAD91046 EESTIFHEAINFTTTHLEEYVKKHND-----DDGYFSALVNHALELPLHWR-MVRLEARW 276

Cs5g15530 -EEKILEKAKQFSARFLREKQAANKLLDKWIMAKDLPGEVGYALDIPWFAS-LPRIEARF 418

Cs5g31210 -GEKILGHAKQFSAKYLKAKTA-ADLLDKWIIAKDLPGEVAFAMEVPWYAC-LPRLETRL 498

Cs2g06470 -EEYELQEARSFARKSLEKTMTTGSRGPDDVTFTSFRKVIEHELDFPWLTR-MEHLEYRM 427

orange1.1t03278 PDESVLEKQQFWTSHFLKQELSSGSI-HSNRFSQNVSSQVEDALKFPYHVN-LERLAHRR 424

Cs2g22180 YINEYSQS--------------WNHDQTLLQLAKSDFNMVQSLHQRELAEIK-------- 273

Cs2g07250 VFFGNFQGER------------RWMLHVLKEIAKKDFNMVHALHHKEIVQVT-------- 287

BAP75559 VFLSNFQGE--------------RRLHVLKEIAKKDFNMVQALHQKEIVQVT-------- 285

Cs2g07240 FFHGNFQGNE------------YVWILDLKELANMDFKLVQSLHQKEIVQIS-------- 283

BAP75560 FFHGNFQGSE------------YIWILDLQELANMDFKLVQSLHQKEIVQIS-------- 276

orange1.1t02448 FFHGNFQGNE------------YIRILDLQELANMDFKLVQSLHQKEIVQIS-------- 276

orange1.1t03307 FFHGNFQGNE------------YIWILDLEELANMDFKLVQSLHQKEIVQIS-------- 276

orange1.1t02008 FISFYEH--D------------DSKNDTLLKFAKLDFNRVQLLHQQELAYIT-------- 291

Cs4g11980 YISIYEA-DN------------STRNELILELAKLDFNLLQELHRRELSEIS-------- 265

orange1.1t04360.1 YISIYEA-DN------------STRNELILELAKLDFNLLQALHRIELSEIS-------- 266

orange1.1t04360.2 YISIYEA-DN------------STRNELILELAKLDFNLLQALHRIELSEIS-------- 266

Cs4g12080 YIDLYSQ-DD------------SKDKAILLKFAKLDFSMLQVIHRKELSIIT-------- 257

Cs4g12050 YINLYSQ-DD------------SKDKAILLKFAKLDFCMLQGIHRKELSIIT-------- 257

Cs4g12060 YIDLYSR-DD------------SKDKAILLKFAKLDFCMLQVIHRKELSIIT-------- 257

Cs5g12880 FMSMINSTSD------------HLHNKTLLNFAKLDFNILLELHKEELNELT-------- 248

Cs5g12900.2 FMSMINSTSD------------HLHNKTLLNFAKLDFNILLELHKEELNELT-------- 248

AAQ04608 FMSMINSTSD------------HLCNKTLLNFAKLDFNILLELHKEELNELT-------- 248

Cs4g12350 FLSVYPR--V------------DKHDKTLLKFAKLDFNLVQRIHQKELSAIT-------- 256

Cs4g12400 FLSVYPR--D------------DKHDKTLLKFAKLDFNLVQRIHQKELSAIT-------- 256

Cs4g12220 FLDIYSR--D------------DLHDKTLLNFAKLDFNILQAMHQKEASEIT-------- 194

Cs4g12480 FLDIYSR--D------------DLHDETLLKFAKLDFNILQAAHQKEASIMT-------- 252

Cs4g11320 FLDIYSR--D------------DLHDETLLKFAKLDFNILQAAHQKEASIM--------- 181

orange1.1t03302.1 FLDIYSR--D------------DLHDETLLKFAKLDFNILQAAHQKEASIM--------- 257

Q94JS8 FLDIYSR--D------------DLHDETLLKFAKLDFNILQAAHQKEASIMT-------- 258

Cs4g12450 FLDVCSR--G------------DMHDKSLLKFAKLDFNLLQAAHQKEVSDMT-------- 251

orange1.1t00017.2 YLNMYSR--D------------DLHDETLLKFAKLDFNLLQAAHQKELSDMT-------- 252

Cs5g06290 YISIYQD-D-------------ASHYKALLTLAKLDFNLVQSLHKKELCEIS-------- 254

Cs3g21560 FISFSQD-HE------------PLHNKALLKLAKLDFNHVQSLHKIELSEIS-------- 242

Cs3g21590c FISLYQE-HE------------PLHNKALLKLAKLDFNQVQSLYKIELSEIS-------- 242

Cs4g12110.1 YMPIYQK-D-------------PSHNKDLLTFAMLDFNILQKQHQEELRDIV-------- 264

BAP75561 YMPIYQK-D-------------PSHNKDLLTFAMLDFNILQKQHQEELRDIV-------- 264

Cs3g16210 YIPIYQE-Q-------------SSHNEALLTFAKLDFNKLQKLHQKELGDIS-------- 324

Cs2g23470 YIPIYHE-E-------------PSHNEALLTFAKLDFNKLQKLHQKELGDIS-------- 261

Cs2g24110.1 HIPIYQE-E-------------SSHNEALLTFAKLDFNKLQKLHQKELGDIS-------- 162

Cs5g23510.1 YIPIYQG-E-------------SSHNEALLTFAKLDFNRLQKLHQKELGDIS-------- 260

Cs5g23540 YIPIYQG-E-------------SSHNEALLTFAKLDFNGLQKLHQKELGDIS-------- 260

BAP74389 YISIYRE-E-------------SSHNEALLTFAKLDFNKLQKLHQKELSDIS-------- 262

Cs4g12090 YIPIYQE-E-------------SPHNEALLTFAKLDFNKLQKLHQKELGDIS-------- 263

CuSTS1 YIPIYQE-E-------------SPHNEALLTFAKLDFNKLQKLHQKELGDIS-------- 262

Cs4g12120.1 YIPIYQE-E-------------SPHNEALLTFAKLDFNKLQKLHQKELGDIS-------- 216

Cs4g12120.2 YIPIYQE-E-------------SPHNEALLTFAKLDFNKLQKLHQKELGDIS-------- 262

Cs2g22090 FIKLYPT-DD------------E-SSPILLTLAKLDYNLVQSIHQQELKELA-------- 251

Cs2g22100.1 FINLYPT-DV------------PKNLVVLLELAKLDYNLVQSIHQKELKELA-------- 292

Cs2g07280 QINSYEK-EK------------H-MNTILLELAKLNFNIVQATLQNDLRELS-------- 268

Cs2g24530.1 QINSYEK-EK------------H-MNTILLELAKLNFNIVQATLQNDLRELS-------- 250

Cs5g22980 FINVYEK-RK------------D-KNHALLELAKLDFNILQATYQEELKDIS-------- 290

Cs3g04340 FTRKNLK-IF------------E-GI------------------HAWLQLIF-------- 163

BAD27256 FIDVYEK-RE------------D-KNHLLLELAKLEFNVLQAIYQEELKDVS-------- 289

AAM53944 FIHIYER-RE------------D-KNHLLLELAKMEFNTLQAIYQEELKEIS-------- 289

BAF73932 FIHIYER-RE------------D-KNHLLLELAKMEFNTLQAIYQEELKEIS-------- 289

AAM53946 FIHVYEK-RE------------D-KNHLLLELAKMEFNTLQAIYQEELKEIS-------- 289

BAD27257 FIHVYEK-RE------------D-KNHLLLELAKLEFNTLQAIYQEELKDIS-------- 291

AOP12358 FIHVYEK-RE------------D-KNHLLLELAKLEFNTLQAIYQEELKDIS-------- 290

Cs3g04170 FIHVYEK-RE------------D-KNHLLLELAKLEFNTLQAIYQEELKDIS-------- 290

Cs3g04360 FIHVYEK-RE------------D-KNHLLLELAKLEFNTLQAIYQEELKDIS-------- 290

Cs3g04190 FIHVYEK-RE------------D-KNHLLLELAKLEFNTLQAIYQEELKDIS-------- 205

orange1.1t04775.1 FIHVYEK-RE------------D-KNHLLLELAKLEFNTLQAIYQEELKDIS-------- 258

orange1.1t04366 FIHVYEK-RE------------D-KNHLLLELAKLEFNTLQAIYQEELKDIS-------- 223

BAM29049 FIHVYEK-RE------------D-KNHLLLELAKLEFNTLQAIYQEELKDIS-------- 289

Cs2g22150 YTEAYRK-K-------------D-ANCMLLEFAKLDFNRVQSSYQEEIKNLT-------- 201

BAD27258 FIDVYES-GP------------D-MNPILLELAKLDYNIVQAIHQEDLKYVS-------- 300

BAD27259 FIDVYES-GP------------D-MNPILLELAKLDYNIVQAIHQEDLKYVS-------- 300

Cs3g07850 ------------------------------------------------------------ 260

AAM53943 FIDVYES-GP------------D-MNPILLELAKVDYNIVQAVHQEDLKYVS-------- 300

Cs7g18530 FIDVCEN-GP------------D-MNPILLELAKVDFNIVQAVHQENLKYAS-------- 300

Cs3g07920 ------------------------------------------------------------ 260

BAF73933 FIDVYEN-GP------------D-MSPILLELAKVDFNIVQAVHQENLKYAS-------- 300

AAM53945 FIDVYEN-GP------------H-MNPILLELAKVDFNIVQAVHQENLKYAS-------- 300

BAD27260 FIDVYEN-GP------------D-MNPILLELAKVDFNIVQAVHQENLKYAS-------- 300

BAD91045 FIDVYES-GP------------D-MNPILLDLAKLDFNIVQAVHQEDIKYAS-------- 300

Cs3g07880 FIDVYQS-GP------------S-TNPILLDLAKLDFNIVQAVHQEDIKYASSA------ 302

Cs7g17640 FIDVYQS-GP------------N-TNPILLDLAKLDFNIVQAVHQEDIKYASREFRYIIS 308

Cs2g03570 FIDMYQR-GP------------D-VNQVLVELAKLDFNAVQAEHQEELKYVS-------- 311

Cs8g14120.1 FIDVYER-GT------------D-MNPVLVELAKLDFNSVQAAHQDELKYVS-------- 322

BAD91046 FIDVYER-GT------------D-MNPVLVELAKLDFNSVQAAHQDELKYVS-------- 314

Cs5g15530 YIKQYGGEDDVWIGKTLYRMPKI-SNNFYLQLAKLDYNNCQAIHQSEWISMQ-------- 469

Cs5g31210 YIEQYGGEDDVWIGKTLYRMPYV-NNDVYLELAKLDYNNCQAVHRTEWDSLQ-------- 549

Cs2g06470 WIEEKD-MNALWMGKASFHRLSCSYNDKLMQLAIQNYGFRQLVYKRELEELK-------- 478

orange1.1t03278 NINLYN-VDNMRILKTSYCSLNI-GNEYFQKLAVDDFNICQSMHIEELKHLE-------- 474

Cs2g22180 -------------------RWWKQLGLVDKL----------------------------- 285

Cs2g07250 -------------------KWWKDLGLAKKL----------------------------- 299

BAP75559 -------------------KWWKDLGLTKKL----------------------------- 297

Cs2g07240 -------------------KWWKELGLAKKL----------------------------- 295

BAP75560 -------------------SWWRELGLAKKL----------------------------- 288

orange1.1t02448 -------------------SWWRELGLAKKL----------------------------- 288

orange1.1t03307 -------------------SWWRELGLAKKL----------------------------- 288

orange1.1t02008 -------------------RWMKGLNFGSTY----------------------------- 303

Cs4g11980 -------------------RWWKDIDFATKL----------------------------- 277

orange1.1t04360.1 -------------------RWWKDIDFATKL----------------------------- 278

orange1.1t04360.2 -------------------RWWKDIDFATKL----------------------------- 278

Cs4g12080 -------------------EWWKILDVEINL----------------------------- 269

Cs4g12050 -------------------RWWKNLDVEINL----------------------------- 269

Cs4g12060 -------------------EWWKNLDVEINL----------------------------- 269

Cs5g12880 -------------------KWWKDLDFTTKL----------------------------- 260

Cs5g12900.2 -------------------KWWKDLDFTTKL----------------------------- 260

AAQ04608 -------------------KWWKDLDFTTKL----------------------------- 260

Cs4g12350 -------------------RWWKDLDFTTKL----------------------------- 268

Cs4g12400 -------------------RWWKDLDFTTKL----------------------------- 268

Cs4g12220 -------------------RWWRDLDFLKKL----------------------------- 206

Cs4g12480 -------------------RWWNDLGFPKKV----------------------------- 264

Cs4g11320 ------------------------------------------------------------ 181

orange1.1t03302.1 ------------------------------------------------------------ 257

Q94JS8 -------------------RWWNDLGFPKKV----------------------------- 270

Cs4g12450 -------------------RWWIDLDFSTKL----------------------------- 263

orange1.1t00017.2 -------------------RWWKDLDIPTKL----------------------------- 264

Cs5g06290 -------------------RWWKDLDFARKL----------------------------- 266

Cs3g21560 -------------------RWWKDLNFAGKL----------------------------- 254

Cs3g21590c -------------------RWWKDLDFARKL----------------------------- 254

Cs4g12110.1 -------------------RWWKNFDVPNKL----------------------------- 276

BAP75561 -------------------RWWKNFDVPNKL----------------------------- 276

Cs3g16210 -------------------RWDISAIDQLPEYMKLCYRALLDVYSEAEKDLAPQGKLYRL 365

Cs2g23470 -------------------RWWKELDFTHKL----------------------------- 273

Cs2g24110.1 -------------------RWWKELDFTHKL----------------------------- 174

Cs5g23510.1 -------------------RWWKELDFAHKL----------------------------- 272

Cs5g23540 -------------------RWWKELDFAHKL----------------------------- 272

BAP74389 -------------------RWWKELDFAHNL----------------------------- 274

Cs4g12090 -------------------RWWKELDFAHKL----------------------------- 275

CuSTS1 -------------------RWWKELDFAHKL----------------------------- 274

Cs4g12120.1 -------------------RWWKELDFAHKL----------------------------- 228

Cs4g12120.2 -------------------RWWKELDFAHKL----------------------------- 274

Cs2g22090 -------------------RWWSNLGFKEKL----------------------------- 263

Cs2g22100.1 -------------------RWWRELGFHETL----------------------------- 304

Cs2g07280 -------------------RWWKNLGLIENL----------------------------- 280

Cs2g24530.1 -------------------RWWKNLGLIENL----------------------------- 262

Cs5g22980 -------------------GWWKDKGLGEKL----------------------------- 302

Cs3g04340 -------------------MWWKDIGLAEKL----------------------------- 175

BAD27256 -------------------RWWKDIGLGEKL----------------------------- 301

AAM53944 -------------------GWWKDTGLGEKL----------------------------- 301

BAF73932 -------------------GWWKDTGLGEKL----------------------------- 301

AAM53946 -------------------GWWKDTGLGEKL----------------------------- 301

BAD27257 -------------------GWWKDTGLGEKL----------------------------- 303

AOP12358 -------------------GWWKDTGLGEKL----------------------------- 302

Cs3g04170 -------------------GWWKDTGLGEKL----------------------------- 302

Cs3g04360 -------------------GWWKDTALGEKL----------------------------- 302

Cs3g04190 -------------------GWWKDTGLGEKG----------------------------- 217

orange1.1t04775.1 -------------------EWWKDIGLAEKL----------------------------- 270

orange1.1t04366 -------------------GWWKDTGLGEKL----------------------------- 235

BAM29049 -------------------GWWKETGLGEKL----------------------------- 301

Cs2g22150 -------------------RWWEDIDLAKNL----------------------------- 213

BAD27258 -------------------RWWMKTGLGEKL----------------------------- 312

BAD27259 -------------------RWWMKTGLGEKL----------------------------- 312

Cs3g07850 -------------------RWWKKTGLGEKL----------------------------- 272

AAM53943 -------------------RWWKKTGLGEKL----------------------------- 312

Cs7g18530 -------------------RWWKKTGLGENL----------------------------- 312

Cs3g07920 -------------------RWWKKTGLGEKL----------------------------- 272

BAF73933 -------------------RWWKKTGLGENL----------------------------- 312

AAM53945 -------------------RWWKKTGLGENL----------------------------- 312

BAD27260 -------------------RWWKKTGLGENL----------------------------- 312

BAD91045 -------------------RWWKKIGLGERL----------------------------- 312

Cs3g07880 ------------------------------------------------------------ 302

Cs7g17640 LEFFSASLHLWKCGDPHTRMWWKKTGLGERL----------------------------- 339

Cs2g03570 -------------------RWWRKTGLGE-L----------------------------- 322

Cs8g14120.1 -------------------WWWRKTGLGE-L----------------------------- 333

BAD91046 -------------------WWWRKTGLGE-L----------------------------- 325

Cs5g15530 -------------------RWYTKCKLEN-F----------------------------- 480

Cs5g31210 -------------------KWYAACKLDK-F----------------------------- 560

Cs2g06470 -------------------RWSRDNNLSD-M----------------------------- 489

orange1.1t03278 -------------------RWVVEKRLDK-L----------------------------- 485

**RDR**

Cs2g22180 ---------GFG**RDR**PLECFL---WT-VGIFPEPYYSN---------------------- 310

Cs2g07250 ---------PFA**RD**QPLKWYI---WS-MACLTDPSLSE---------------------- 324

BAP75559 ---------PFA**RD**QPLKWYI---WS-MACLTDPSLSE---------------------- 322

Cs2g07240 ---------EFA**RD**QPLKWYM---WS-MACFTDPSLSW---------------------- 320

BAP75560 ---------EFA**R**EQPVKWYV---WS-MACFTDPNLSW---------------------- 313

orange1.1t02448 ---------EFA**R**EQPVKWYV---WS-MACFTDPNLSW---------------------- 313

orange1.1t03307 ---------EFA**R**EQPVKWYV---WS-MACFTDPNLSW---------------------- 313

orange1.1t02008 ---------SYS**RDR**TVEIYL---WS-VAQYFEPHFSR---------------------- 328

Cs4g11980 ---------PFA**RDR**LVECYF---WI-LGVYFEPKYST---------------------- 302

orange1.1t04360.1 ---------PFA**RDR**LVECYF---WI-LGVYFEPKYST---------------------- 303

orange1.1t04360.2 ---------PFA**RDR**LVECYF---WI-LGVYFEPKYST---------------------- 303

Cs4g12080 ---------PYA**R**N**R**VVECYF---WA-MGVYFEPRYSF---------------------- 294

Cs4g12050 ---------PYA**R**N**R**VVECYF---WA-MGVYFEPRYSF---------------------- 294

Cs4g12060 ---------PYA**R**N**R**VVECYF---WA-MGVYFEPRYSF---------------------- 294

Cs5g12880 ---------PYA**R**D**R**LVELYF---WD-LGTYFEPQYAF---------------------- 285

Cs5g12900.2 ---------PYA**R**D**R**LVELYF---WD-LGTYFEPQYAF---------------------- 285

AAQ04608 ---------PYA**R**D**R**LVELYF---WD-LGTYFEPQYAF---------------------- 285

Cs4g12350 ---------PYA**R**D**R**IVELYF---WI-VGTYFEPKYTL---------------------- 293

Cs4g12400 ---------PYA**R**D**R**IVELYF---WI-VGTYFEPKYTL---------------------- 293

Cs4g12220 ---------PYI**R**E**R**VVELYF---WMLVGVSYEPNFST---------------------- 232

Cs4g12480 ---------PYA**RDR**IVETYI---WMLLGVSYEPNLAF---------------------- 290

Cs4g11320 ------------------------------------------------------------ 181

orange1.1t03302.1 ------------------------------------------------------------ 257

Q94JS8 ---------PYA**RDR**IIETYI---WMLLGVSYEPNLAF---------------------- 296

Cs4g12450 ---------AYA**RDR**IVELYF---WILMGAYYEPKYAF---------------------- 289

orange1.1t00017.2 ---------PYA**RDR**MVEVYF---WTLVGVYYEPKYTF---------------------- 290

Cs5g06290 ---------PFA**RDR**MVECFF---WI-LGVYFEPNYSL---------------------- 291

Cs3g21560 ---------PFA**RDR**VVESYF---WI-VGVYFEPEYSL---------------------- 279

Cs3g21590c ---------PFA**RDR**VVECYF---WI-VGVYFEPQYSL---------------------- 279

Cs4g12110.1 ---------PFI**RDR**VVEGYF---WI-LGVYFEPKFLL---------------------- 301

BAP75561 ---------PFI**RDR**VVEGYF---WI-LGVYFEPKFLL---------------------- 301

Cs3g16210 HYAKKAVEYSSMKNIVKNCFFEAKWC-HQNYIPTMDEYMTVALVTSAYPMLSTTSFVGMG 424

Cs2g23470 --------PF-I**RDR**VAEGYF---WA-IGAYFEPQHSF---------------------- 298

Cs2g24110.1 --------PF-I**RDR**VAELYF---WV-IGVYFEPQYSF---------------------- 199

Cs5g23510.1 --------PF-V**RDR**IAEGYF---WA-VGVYFEPQYSF---------------------- 297

Cs5g23540 --------PF-V**RDR**IAEVYF---WA-VGVHFEPQYSF---------------------- 297

BAP74389 --------PFTI**RDR**IAECYF---WA-VAVYFEPQYSL---------------------- 300

Cs4g12090 --------PF-I**RDR**VAECYF---WI-LGVYFEPQYSF---------------------- 300

CuSTS1 --------PF-I**RDR**VAECYF---WI-LGVYFEPQYSF---------------------- 299

Cs4g12120.1 --------PF-I**RDR**VAECYF---WI-LGVYFEPQYSF---------------------- 253

Cs4g12120.2 --------PF-I**RDR**VAECYF---WI-LGVYFEPQYSF---------------------- 299

Cs2g22090 ---------SFA**RDR**LMENYL---LV-MGLCFKAQFSK---------------------- 288

Cs2g22100.1 ---------TFS**RDR**LMENYL---WA-MGIVFEPQFTK---------------------- 329

Cs2g07280 ---------NFS**RDR**LVECFL---CA-VGLVYEPNCSC---------------------- 305

Cs2g24530.1 ---------NFS**RDR**LVECFL---CA-VGLVYEPNCSC---------------------- 287

Cs5g22980 ---------SFA**R**S**R**LVTSFF---WG-MGMVFEPQFAY---------------------- 327

Cs3g04340 ---------SFA**RD**SLVASFVRSMGS-MGIVFKPQLAY---------------------- 203

BAD27256 ---------SFA**RD**SLVASFV---WS-MGIVFEPQFAY---------------------- 326

AAM53944 ---------SFA**R**N**R**LVASFL---WS-MGIAFEPQFAY---------------------- 326

BAF73932 ---------SFA**R**N**R**LVASFL---WS-MGIAFEPQFAY---------------------- 326

AAM53946 ---------SFA**R**N**R**LVASFL---WS-MGIAFEPQFAY---------------------- 326

BAD27257 ---------SFA**R**N**R**LVASFL---WS-MGIAFEPQFAY---------------------- 328

AOP12358 ---------SFA**R**N**R**LVASFL---WS-MGIAFEPQFAY---------------------- 327

Cs3g04170 ---------SFA**R**N**R**LVASFL---WS-MGIAFEPQFAY---------------------- 327

Cs3g04360 ---------SFA**R**N**R**LVASFL---WS-MGIAFEPQFAY---------------------- 327

Cs3g04190 ---------LGEKLSFAASFL---WS-MGIGSEPQFAF---------------------- 242

orange1.1t04775.1 ---------SFA**RD**SLVASFVRSMGS-MGIVFKPQLAY---------------------- 298

orange1.1t04366 ---------SFA**RD**SLVASFL---WS-MGVGSEPQFAY---------------------- 260

BAM29049 ---------SFA**RD**SLVASFL---WS-MGIGSEPQFAY---------------------- 326

Cs2g22150 ---------KFA**RDR**LMECFI---WS-VGMVPKPQYSN---------------------- 238

BAD27258 ---------NFA**RDR**VVENFF---WT-VGDIFEPQFGY---------------------- 337

BAD27259 ---------NFA**RDR**VVENFF---WT-VGDIFEPQFGY---------------------- 337

Cs3g07850 ---------NFA**RDR**VVENFF---WT-VGDIFEPQFGY---------------------- 297

AAM53943 ---------NFA**RDR**VVENFF---WT-VGDIFEPQFGY---------------------- 337

Cs7g18530 ---------NFV**RDR**IVENFM---WT-VGEKFEPQFGY---------------------- 337

Cs3g07920 ---------NFA**RDR**VVENFF---WT-VGDIFEPQFGY---------------------- 297

BAF73933 ---------NFV**RDR**IVENFL---WT-VGEKFEPQFGY---------------------- 337

AAM53945 ---------NFV**RDR**IVENFM---WT-VGEKFEPQFGY---------------------- 337

BAD27260 ---------NFV**RDR**IVENFM---WT-VGEKFEPQFGY---------------------- 337

BAD91045 ---------NFA**RDR**IMENFF---WT-VGVIFEPNFGY---------------------- 337

Cs3g07880 -------------ETSGKTYK---LC-GL------------------------------- 314

Cs7g17640 ---------NFA**RDR**IMENFF---WT-VGVIFEPNFGY---------------------- 364

Cs2g03570 ---------HFA**RDR**IMENFF---WA-LGEVWEPQFGY---------------------- 347

Cs8g14120.1 ---------HFA**RDR**IVENFF---WA-LGEIWEPQFGY---------------------- 358

BAD91046 ---------HFA**RDR**ILENFF---WA-LGEIWEPQFGY---------------------- 350

Cs5g15530 ---------GTSKRALLLAYFVASAS-I--YEPERKR----------------------- 505

Cs5g31210 ---------GITRRALLLAYFVASAS-I--YEPARWQ----------------------- 585

Cs2g06470 ---------GFGREKTTYCYFATAAA-IGVSLPYDSH----------------------- 516

orange1.1t03278 ---------KFARQKQTYCYFSVAAT-L--FSPELSD----------------------- 510

Cs2g22180 ------------------------------------------------------------ 310

Cs2g07250 ------------------------------------------------------------ 324

BAP75559 ------------------------------------------------------------ 322

Cs2g07240 ------------------------------------------------------------ 320

BAP75560 ------------------------------------------------------------ 313

orange1.1t02448 ------------------------------------------------------------ 313

orange1.1t03307 ------------------------------------------------------------ 313

orange1.1t02008 ------------------------------------------------------------ 328

Cs4g11980 ------------------------------------------------------------ 302

orange1.1t04360.1 ------------------------------------------------------------ 303

orange1.1t04360.2 ------------------------------------------------------------ 303

Cs4g12080 ------------------------------------------------------------ 294

Cs4g12050 ------------------------------------------------------------ 294

Cs4g12060 ------------------------------------------------------------ 294

Cs5g12880 ------------------------------------------------------------ 285

Cs5g12900.2 ------------------------------------------------------------ 285

AAQ04608 ------------------------------------------------------------ 285

Cs4g12350 ------------------------------------------------------------ 293

Cs4g12400 ------------------------------------------------------------ 293

Cs4g12220 ------------------------------------------------------------ 232

Cs4g12480 ------------------------------------------------------------ 290

Cs4g11320 ------------------------------------------------------------ 181

orange1.1t03302.1 ------------------------------------------------------------ 257

Q94JS8 ------------------------------------------------------------ 296

Cs4g12450 ------------------------------------------------------------ 289

orange1.1t00017.2 ------------------------------------------------------------ 290

Cs5g06290 ------------------------------------------------------------ 291

Cs3g21560 ------------------------------------------------------------ 279

Cs3g21590c ------------------------------------------------------------ 279

Cs4g12110.1 ------------------------------------------------------------ 301

BAP75561 ------------------------------------------------------------ 301

Cs3g16210 DIVTKESFEWLFSNPRFIRASSVVCRLMDDMASHEFEQSRGHVASSVECYMKQHGAEEEA 484

Cs2g23470 ------------------------------------------------------------ 298

Cs2g24110.1 ------------------------------------------------------------ 199

Cs5g23510.1 ------------------------------------------------------------ 297

Cs5g23540 ------------------------------------------------------------ 297

BAP74389 ------------------------------------------------------------ 300

Cs4g12090 ------------------------------------------------------------ 300

CuSTS1 ------------------------------------------------------------ 299

Cs4g12120.1 ------------------------------------------------------------ 253

Cs4g12120.2 ------------------------------------------------------------ 299

Cs2g22090 ------------------------------------------------------------ 288

Cs2g22100.1 ------------------------------------------------------------ 329

Cs2g07280 ------------------------------------------------------------ 305

Cs2g24530.1 ------------------------------------------------------------ 287

Cs5g22980 ------------------------------------------------------------ 327

Cs3g04340 ------------------------------------------------------------ 203

BAD27256 ------------------------------------------------------------ 326

AAM53944 ------------------------------------------------------------ 326

BAF73932 ------------------------------------------------------------ 326

AAM53946 ------------------------------------------------------------ 326

BAD27257 ------------------------------------------------------------ 328

AOP12358 ------------------------------------------------------------ 327

Cs3g04170 ------------------------------------------------------------ 327

Cs3g04360 ------------------------------------------------------------ 327

Cs3g04190 ------------------------------------------------------------ 242

orange1.1t04775.1 ------------------------------------------------------------ 298

orange1.1t04366 ------------------------------------------------------------ 260

BAM29049 ------------------------------------------------------------ 326

Cs2g22150 ------------------------------------------------------------ 238

BAD27258 ------------------------------------------------------------ 337

BAD27259 ------------------------------------------------------------ 337

Cs3g07850 ------------------------------------------------------------ 297

AAM53943 ------------------------------------------------------------ 337

Cs7g18530 ------------------------------------------------------------ 337

Cs3g07920 ------------------------------------------------------------ 297

BAF73933 ------------------------------------------------------------ 337

AAM53945 ------------------------------------------------------------ 337

BAD27260 ------------------------------------------------------------ 337

BAD91045 ------------------------------------------------------------ 337

Cs3g07880 ------------------------------------------------------------ 314

Cs7g17640 ------------------------------------------------------------ 364

Cs2g03570 ------------------------------------------------------------ 347

Cs8g14120.1 ------------------------------------------------------------ 358

BAD91046 ------------------------------------------------------------ 350

Cs5g15530 ------------------------------------------------------------ 505

Cs5g31210 ------------------------------------------------------------ 585

Cs2g06470 ------------------------------------------------------------ 516

orange1.1t03278 ------------------------------------------------------------ 510

**DD**IF**D**

Cs2g22180 -------------------------CRIELAKTIALLLVI**DD**IF**D**TYGSLS--------- 336

Cs2g07250 -------------------------QRVELTKPISLIYII**DD**IF**D**VYGTLD--------- 350

BAP75559 -------------------------QRVELTKPISLIYII**DD**IF**D**VYGTLD--------- 348

Cs2g07240 -------------------------QRIELTKPISFIYII**DD**IF**D**IYGALD--------- 346

BAP75560 -------------------------QRIELTKPISFVYII**DD**IFYVCGALD--------- 339

orange1.1t02448 -------------------------QRIELTKPISFVYII**DD**IF**D**VYGALD--------- 339

orange1.1t03307 -------------------------QRIELTKPISFVYII**DD**IF**D**VYGALD--------- 339

orange1.1t02008 -------------------------GRIIFTKIYLLLLII**DD**TY**D**AYGTFG--------- 354

Cs4g11980 -------------------------TRKFMTKIIAIASVI**DD**IY**D**VYGTLE--------- 328

orange1.1t04360.1 -------------------------TRKFMTKIIAIASVI**DD**IY**D**VYGTLE--------- 329

orange1.1t04360.2 -------------------------TRKFMTKIIAIASVI**DD**IY**D**VYGTLE--------- 329

Cs4g12080 -------------------------ARKILSKVIAMASIL**DD**TY**D**AYGTLE--------- 320

Cs4g12050 -------------------------ARKILSKVIAMASIL**DD**TY**D**AYGTLE--------- 320

Cs4g12060 -------------------------ARKIFSKVIAMASIL**DD**TY**D**AYGTLE--------- 320

Cs5g12880 -------------------------GRKIMTQLNYILSII**DD**TY**D**AYGTLE--------- 311

Cs5g12900.2 -------------------------GRKIMTQLNYILSII**DD**TY**D**AYGTLE--------- 311

AAQ04608 -------------------------GRKIMTQLNYILSII**DD**TY**D**AYGTLE--------- 311

Cs4g12350 -------------------------ARKIMTKTIYMASII**DD**TF**D**AYGFFE--------- 319

Cs4g12400 -------------------------ARKIMTKTIYTASII**DD**TF**D**AYGFFE--------- 319

Cs4g12220 -------------------------GRIFLSKIICLETLV**DD**TF**D**AYGTFE--------- 258

Cs4g12480 -------------------------GRIFASKVMCMLTII**DD**TF**D**AYGTFE--------- 316

Cs4g11320 ------------------------------------------------------------ 181

orange1.1t03302.1 ------------------------------------------------------------ 257

Q94JS8 -------------------------GRIFASKVVCMITTI**DD**TF**D**AYGTFE--------- 322

Cs4g12450 -------------------------GRIFMSKLISMISIL**DD**TF**D**AYGTYD--------- 315

orange1.1t00017.2 -------------------------GRILVSKIICLISLI**DD**TF**D**AYGTFE--------- 316

Cs5g06290 -------------------------ARRILTKVIAMTSII**DD**IY**D**VYGTPE--------- 317

Cs3g21560 -------------------------ARKLLAKILSMTSII**DD**IY**D**VFATPK--------- 305

Cs3g21590c -------------------------ARKLLTKVISMTSII**DD**IY**D**VYGIPE--------- 305

Cs4g12110.1 -------------------------ARKILTKVISMASII**DD**IY**D**AYGTIE--------- 327

BAP75561 -------------------------ARKILTKVISMASII**DD**IY**D**AYGTIE--------- 327

Cs3g16210 CNEFRKQVSNAWKDINEDCLRPTVVPMPLLMRILNLTRVI**D**VIYNCVADVFKKFKDTDGN 544

Cs2g23470 -------------------------ARRLFTKVITLISVI**DD**IY**D**VYGKIE--------- 324

Cs2g24110.1 -------------------------ARRLFTKVLSLTSII**DD**IY**D**VYGKIE--------- 225

Cs5g23510.1 -------------------------ARKLFTKVVYMTSII**DD**IY**D**VYGKIE--------- 323

Cs5g23540 -------------------------ARKLFTKVIYMTSII**DD**IY**D**VYGKIE--------- 323

BAP74389 -------------------------GRRMLAKVFPMTSII**DD**IY**D**VYGKFE--------- 326

Cs4g12090 -------------------------ARRILTKVISMTSVI**DD**IY**D**VYGKIE--------- 326

CuSTS1 -------------------------ARRILTKVISMTSVI**DD**IY**D**VYGKIE--------- 325

Cs4g12120.1 -------------------------ARRILTKVISMTSVI**D**DIY**D**VYGKIE--------- 279

Cs4g12120.2 -------------------------ARRILTKVISMTSVI**DD**IY**D**VYGKIE--------- 325

Cs2g22090 -------------------------CRIGLTKFVCILTAI**DD**IY**D**VYGSID--------- 314

Cs2g22100.1 -------------------------CRIELTKFVCILTAI**DD**MY**D**IYGSLE--------- 355

Cs2g07280 -------------------------FRKWLTKVIIFILVI**DD**IY**D**IYGSLE--------- 331

Cs2g24530.1 -------------------------FRKWLTKVIIFILVI**DD**IY**D**IYGSLE--------- 313

Cs5g22980 -------------------------SRRVLTITLALITVI**DD**IY**D**IYGTLD--------- 353

Cs3g04340 -------------------------CRRIRTITFALISVI**DD**IY**D**VYGTLD--------- 229

BAD27256 -------------------------CRRILTITFALISVI**DD**IY**D**VYGTLD--------- 352

AAM53944 -------------------------CRRVLTISIALITVI**DD**IY**D**VYGTLD--------- 352

BAF73932 -------------------------CRRVLTISIALITVI**DD**IY**D**VYGTLD--------- 352

AAM53946 -------------------------CRRVLTISIALITVI**DD**IY**D**VYGTLD--------- 352

BAD27257 -------------------------CRRVLTISIALITVI**DD**IY**D**VYGTLD--------- 354

AOP12358 -------------------------CRRVLTISIALITVI**DD**IY**D**VYGTLD--------- 353

Cs3g04170 -------------------------CRRVLTISIALITVI**DD**IY**D**VYGTLD--------- 353

Cs3g04360 -------------------------CRRVLTISIALITVI**DD**IY**D**VYGTLD--------- 353

Cs3g04190 -------------------------CRRIVTIAIALITVI**DD**IY**D**VYGTLD--------- 268

orange1.1t04775.1 -------------------------CRRIRTITFALISVI**DD**IY**D**VYGTLD--------- 324

orange1.1t04366 -------------------------CRRIVTIAIALITVI**DD**IY**D**VYGTLD--------- 286

BAM29049 -------------------------CRRIVTIAIALITVI**DD**IY**D**VYGTLD--------- 352

Cs2g22150 -------------------------CRRALTKVAAFVTII**D**YIYAVYGTLD--------- 264

BAD27258 -------------------------CRRMSAMVNCLLTSI**DD**VY**D**VYGTLD--------- 363

BAD27259 -------------------------CRRMSAMVNCLLTSI**DD**VY**D**VYGTLD--------- 363

Cs3g07850 -------------------------CRRMSAMVNCLLTSI**DD**VY**D**VYGTLD--------- 323

AAM53943 -------------------------CRRMSAMVNCLLTSI**DD**VY**D**VYGTLD--------- 363

Cs7g18530 -------------------------FRRMSTMVNALITAV**DD**VY**D**VYGTLD--------- 363

Cs3g07920 -------------------------CRRMSAMVNCLLTSI**DD**VY**D**VYGTLD--------- 323

BAF73933 -------------------------FRRMSTMVIALITAV**DD**VY**D**VYGTLD--------- 363

AAM53945 -------------------------FRRMSTMVNALITAV**DD**VY**D**VYGTLE--------- 363

BAD27260 -------------------------FRRMSTMVNALITAV**DD**VY**D**VYGTLD--------- 363

BAD91045 -------------------------CRRMSTMVNALITTI**DD**VY**D**VYGTLD--------- 363

Cs3g07880 ------------------------------------------------------------ 314

Cs7g17640 -------------------------CRRMSTMVNALITTI**DD**VY**D**VYGTLD--------- 390

Cs2g03570 -------------------------CRRMSTKANALITTI**DD**VY**D**VYGTLD--------- 373

Cs8g14120.1 -------------------------CRRMSTKVNALITTV**DD**VY**D**VYGTLD--------- 384

BAD91046 -------------------------CRRMSTKVNALITTI**DD**VY**D**VYGTLD--------- 376

Cs5g15530 -------------------------ERFAWAKAAVLVETIASYFSNQQDSR--------- 531

Cs5g31210 -------------------------ERIAWAKTAVLVETITSYF**D**KQKDSC--------- 611

Cs2g06470 -------------------------VRLILAKSAILITVA**DD**FF**D**MEGSLN--------- 542

orange1.1t03278 -------------------------ARMSWAKNAVLTTIV**DD**FY**D**LGGSEE--------- 536

Cs2g22180 ------------------------------------------------------------ 336

Cs2g07250 ------------------------------------------------------------ 350

BAP75559 ------------------------------------------------------------ 348

Cs2g07240 ------------------------------------------------------------ 346

BAP75560 ------------------------------------------------------------ 339

orange1.1t02448 ------------------------------------------------------------ 339

orange1.1t03307 ------------------------------------------------------------ 339

orange1.1t02008 ------------------------------------------------------------ 354

Cs4g11980 ------------------------------------------------------------ 328

orange1.1t04360.1 ------------------------------------------------------------ 329

orange1.1t04360.2 ------------------------------------------------------------ 329

Cs4g12080 ------------------------------------------------------------ 320

Cs4g12050 ------------------------------------------------------------ 320

Cs4g12060 ------------------------------------------------------------ 320

Cs5g12880 ------------------------------------------------------------ 311

Cs5g12900.2 ------------------------------------------------------------ 311

AAQ04608 ------------------------------------------------------------ 311

Cs4g12350 ------------------------------------------------------------ 319

Cs4g12400 ------------------------------------------------------------ 319

Cs4g12220 ------------------------------------------------------------ 258

Cs4g12480 ------------------------------------------------------------ 316

Cs4g11320 ------------------------------------------------------------ 181

orange1.1t03302.1 ------------------------------------------------------------ 257

Q94JS8 ------------------------------------------------------------ 322

Cs4g12450 ------------------------------------------------------------ 315

orange1.1t00017.2 ------------------------------------------------------------ 316

Cs5g06290 ------------------------------------------------------------ 317

Cs3g21560 ------------------------------------------------------------ 305

Cs3g21590c ------------------------------------------------------------ 305

Cs4g12110.1 ------------------------------------------------------------ 327

BAP75561 ------------------------------------------------------------ 327

Cs3g16210 FKTSLAKDVRGMLSLYEATHLGVHEEDILDEALAFTTSHLESIATHQIRSPLVEQVKHAL 604

Cs2g23470 ------------------------------------------------------------ 324

Cs2g24110.1 ------------------------------------------------------------ 225

Cs5g23510.1 ------------------------------------------------------------ 323

Cs5g23540 ------------------------------------------------------------ 323

BAP74389 ------------------------------------------------------------ 326

Cs4g12090 ------------------------------------------------------------ 326

CuSTS1 ------------------------------------------------------------ 325

Cs4g12120.1 ------------------------------------------------------------ 279

Cs4g12120.2 ------------------------------------------------------------ 325

Cs2g22090 ------------------------------------------------------------ 314

Cs2g22100.1 ------------------------------------------------------------ 355

Cs2g07280 ------------------------------------------------------------ 331

Cs2g24530.1 ------------------------------------------------------------ 313

Cs5g22980 ------------------------------------------------------------ 353

Cs3g04340 ------------------------------------------------------------ 229

BAD27256 ------------------------------------------------------------ 352

AAM53944 ------------------------------------------------------------ 352

BAF73932 ------------------------------------------------------------ 352

AAM53946 ------------------------------------------------------------ 352

BAD27257 ------------------------------------------------------------ 354

AOP12358 ------------------------------------------------------------ 353

Cs3g04170 ------------------------------------------------------------ 353

Cs3g04360 ------------------------------------------------------------ 353

Cs3g04190 ------------------------------------------------------------ 268

orange1.1t04775.1 ------------------------------------------------------------ 324

orange1.1t04366 ------------------------------------------------------------ 286

BAM29049 ------------------------------------------------------------ 352

Cs2g22150 ------------------------------------------------------------ 264

BAD27258 ------------------------------------------------------------ 363

BAD27259 ------------------------------------------------------------ 363

Cs3g07850 ------------------------------------------------------------ 323

AAM53943 ------------------------------------------------------------ 363

Cs7g18530 ------------------------------------------------------------ 363

Cs3g07920 ------------------------------------------------------------ 323

BAF73933 ------------------------------------------------------------ 363

AAM53945 ------------------------------------------------------------ 363

BAD27260 ------------------------------------------------------------ 363

BAD91045 ------------------------------------------------------------ 363

Cs3g07880 ------------------------------------------------------------ 314

Cs7g17640 ------------------------------------------------------------ 390

Cs2g03570 ------------------------------------------------------------ 373

Cs8g14120.1 ------------------------------------------------------------ 384

BAD91046 ------------------------------------------------------------ 376

Cs5g15530 ------------------------------------------------------------ 531

Cs5g31210 ------------------------------------------------------------ 611

Cs2g06470 ------------------------------------------------------------ 542

orange1.1t03278 ------------------------------------------------------------ 536

Cs2g22180 -------------------------------------------DLVLFTEA--------- 344

Cs2g07250 -------------------------------------------ELILFTET--------- 358

BAP75559 -------------------------------------------ELILFTET--------- 356

Cs2g07240 -------------------------------------------VLTLFTEA--------- 354

BAP75560 -------------------------------------------ALTLFTEP--------- 347

orange1.1t02448 -------------------------------------------ALTLFTEA--------- 347

orange1.1t03307 -------------------------------------------ALTLFTEA--------- 347

orange1.1t02008 -------------------------------------------ELQRFTD---------- 361

Cs4g11980 -------------------------------------------ELKLFTHAI-------- 337

orange1.1t04360.1 -------------------------------------------ELKLFTHAIESIH---- 342

orange1.1t04360.2 -------------------------------------------ELKLFTHAI-------- 338

Cs4g12080 -------------------------------------------ELELFTN---------- 327

Cs4g12050 -------------------------------------------ELELFTN---------- 327

Cs4g12060 -------------------------------------------ELELFTN---------- 327

Cs5g12880 -------------------------------------------ELSLFTE---------- 318

Cs5g12900.2 -------------------------------------------ELSLFTE---------- 318

AAQ04608 -------------------------------------------ELSLFTE---------- 318

Cs4g12350 -------------------------------------------ELKLFVE---------- 326

Cs4g12400 -------------------------------------------ELKLFAE---------- 326

Cs4g12220 -------------------------------------------ELKIFTE---------- 265

Cs4g12480 -------------------------------------------QLTHFT----------- 322

Cs4g11320 ------------------------------------------------------------ 181

orange1.1t03302.1 ------------------------------------------------------------ 257

Q94JS8 -------------------------------------------ELTLFTE---------- 329

Cs4g12450 -------------------------------------------ELKLFVE---------- 322

orange1.1t00017.2 -------------------------------------------ELTLFTE---------- 323

Cs5g06290 -------------------------------------------ELKLFTE---------- 324

Cs3g21560 -------------------------------------------ELDLFTA---------- 312

Cs3g21590c -------------------------------------------ELDLFTA---------- 312

Cs4g12110.1 -------------------------------------------ELELFAT---------- 334

BAP75561 -------------------------------------------ELELFAT---------- 334

Cs3g16210 VQPIHRGLQRLEARHYIPIYQEQSSHNEALLTFAKLDFNKLQKLHQKELG---------- 654

Cs2g23470 -------------------------------------------ELELFTS---------- 331

Cs2g24110.1 -------------------------------------------ELELFTS---------- 232

Cs5g23510.1 -------------------------------------------ELELFTS---------- 330

Cs5g23540 -------------------------------------------ELDLFTS---------- 330

BAP74389 -------------------------------------------ELELFTS---------- 333

Cs4g12090 -------------------------------------------ELELFTS---------- 333

CuSTS1 -------------------------------------------ELELFTS---------- 332

Cs4g12120.1 -------------------------------------------ELELFTS---------- 286

Cs4g12120.2 -------------------------------------------ELELFTS---------- 332

Cs2g22090 -------------------------------------------ELELFTEAVKSFPPSYI 331

Cs2g22100.1 -------------------------------------------ELELFTDAV-------- 364

Cs2g07280 -------------------------------------------ELEHFTSA--------- 339

Cs2g24530.1 -------------------------------------------ELEHFTSA--------- 321

Cs5g22980 -------------------------------------------ELELFTNA--------- 361

Cs3g04340 -------------------------------------------ELELFADA--------- 237

BAD27256 -------------------------------------------ELELFADA--------- 360

AAM53944 -------------------------------------------ELEIFTDA--------- 360

BAF73932 -------------------------------------------ELEIFTDA--------- 360

AAM53946 -------------------------------------------ELEIFTDA--------- 360

BAD27257 -------------------------------------------ELEIFTDA--------- 362

AOP12358 -------------------------------------------ELEIFTDA--------- 361

Cs3g04170 -------------------------------------------ELEIFTDA--------- 361

Cs3g04360 -------------------------------------------ELEIFTDA--------- 361

Cs3g04190 -------------------------------------------ELELFTDV--------- 276

orange1.1t04775.1 -------------------------------------------ELELFADA--------- 332

orange1.1t04366 -------------------------------------------GLELFTAA--------- 294

BAM29049 -------------------------------------------ELELFTAA--------- 360

Cs2g22150 -------------------------------------------ELELFTYAVERFVQNFK 281

BAD27258 -------------------------------------------ELELFTDA--------- 371

BAD27259 -------------------------------------------ELELFTDA--------- 371

Cs3g07850 -------------------------------------------ELELFTDA--------- 331

AAM53943 -------------------------------------------ELELFTDA--------- 371

Cs7g18530 -------------------------------------------ELEIFTDA--------- 371

Cs3g07920 -------------------------------------------ELELFTDAL-------- 332

BAF73933 -------------------------------------------ELEIFTDA--------- 371

AAM53945 -------------------------------------------ELEIFTDA--------- 371

BAD27260 -------------------------------------------ELEIFTDA--------- 371

BAD91045 -------------------------------------------ELELFTDA--------- 371

Cs3g07880 ------------------------------------------------------------ 314

Cs7g17640 -------------------------------------------ELELFTDA--------- 398

Cs2g03570 -------------------------------------------ELELFTNA--------- 381

Cs8g14120.1 -------------------------------------------ELEQFTNA--------- 392

BAD91046 -------------------------------------------ELEQFTNA--------- 384

Cs5g15530 -------------------------------------------E---------------- 532

Cs5g31210 -------------------------------------------K---------------- 612

Cs2g06470 -------------------------------------------ELKSLADA--------- 550

orange1.1t03278 -------------------------------------------ELLNLIEL--------- 544

Cs2g22180 ----------------IQRWDLG-AMEHIPEYMKICYMALYN--------------TTNE 373

Cs2g07250 ----------------ITRWDLA-AMGQLPEYMKICFKALDD--------------ITNE 387

BAP75559 ----------------ITRWDLA-AMGQLPEYMKICFKALDD--------------ITNE 385

Cs2g07240 ----------------INRWDLG-GIEQLPECMKICFKALND--------------ITNE 383

BAP75560 ----------------INRWDLG-DIDQLPEYMKICFKALND--------------ITNE 376

orange1.1t02448 ----------------INRWDLG-DIDQLPEYMKICFKALNN--------------ITNE 376

orange1.1t03307 ----------------INRWDLG-DIDQLPEYMKICFKALND--------------ITNE 376

orange1.1t02008 ---------------AVERWDIN-CVSELPEYMKPLYGALLN--------------LFDE 391

Cs4g11980 -----------------ERWEVV-AANELPKYMQVCYFALLD--------------VVKE 365

orange1.1t04360.1 --------------IVFGRWEVV-AANELPKYMQVCYFALLD--------------VVKE 373

orange1.1t04360.2 -----------------ERWEVV-AANELPKYMQVCYFALLD--------------VVKE 366

Cs4g12080 ---------------AIKRWDIS-NIDVLPKYIKLIYQELLD--------------VFGE 357

Cs4g12050 ---------------AIKRWDIS-NIDVLPKYMKLIYQGLLD--------------VFGE 357

Cs4g12060 ---------------A-------------------------------------------- 328

Cs5g12880 ---------------AVQRWNIE-AVDMLPEYMKLIYRTLLD--------------AFNE 348

Cs5g12900.2 ---------------AVQRWNIE-AVDMLPEYMKLIYRTLLD--------------AFNE 348

AAQ04608 ---------------AVQRWNIE-AVDMLPEYMKLIYRTLLD--------------AFNE 348

Cs4g12350 ---------------AVQRWDIG-AMDILPEYMKVLYKALLD--------------TYNE 356

Cs4g12400 ---------------AVQRWDIG-AMDILPEYMKVLYKALLD--------------TFNE 356

Cs4g12220 ---------------AVARWDIG-HIDALPEYMKFIFKTLID--------------LLAM 295

Cs4g12480 ------------------------------------------------------------ 322

Cs4g11320 -----------------TRWDIG-LIDTLPEYMKFIFKALLD--------------IYRE 209

orange1.1t03302.1 -----------------TRWDIG-LIDTLPEYMKFIVKALLD--------------IYRE 285

Q94JS8 ---------------AVTRWDIG-LIDTLPEYMKFIVKALLD--------------IYRE 359

Cs4g12450 ---------------AVKRWDIG-AIDTLPEYMKFIYKSLLD--------------VYDK 352

orange1.1t00017.2 ---------------AVKRWDTN-VTDTLPACMKFIYNKLLG--------------VYNE 353

Cs5g06290 ---------------VIERWDES-SMDQLPEYMQTFFGALLD--------------LYNE 354

Cs3g21560 ---------------AIDRWDMS-CMDQLPEYMQIFYEALLD--------------LYKE 342

Cs3g21590c ---------------AIDRWDVS-CIDQLPEYMQTFYVALLD--------------LYKE 342

Cs4g12110.1 ---------------AIERWDLS-AIDLLPEYIKLCYCALLD--------------AYSE 364

BAP75561 ---------------AIERWDLS-AIDLLPEYMKLCYCALLD--------------AYSE 364

Cs3g16210 ---------------DISRWDIS-AIDQLPEYMKLCYRALLD--------------VYSE 684

Cs2g23470 ---------------AIERWDIN-AIDQLPEYMKLCYGALID--------------VYSE 361

Cs2g24110.1 ---------------AIERWDIN-AIDQLPEYMKLCYRALID--------------VYNE 262

Cs5g23510.1 ---------------AIERWDIN-AIDQLPEYMKLCYRALIN--------------VYSE 360

Cs5g23540 ---------------AIERWDIN-AIDQLPEYMKLCYRALIN--------------VYSE 360

BAP74389 ---------------AIERWDIS-AIDELPEYMKLCYRALLD--------------VYSE 363

Cs4g12090 ---------------AIERWDIS-AIDQLPEYMKLCYRALLD--------------VFSE 363

CuSTS1 ---------------AIERWDIS-AIDQLPEYMKLCYRALLG--------------VFSE 362

Cs4g12120.1 ---------------AIERWDIS-AIDQLPEYMKLCYRALLD--------------VFSE 316

Cs4g12120.2 ---------------AIERWDIS-AIDQLPEYMKLCYRALLD--------------VFSE 362

Cs2g22090 LS------FCFMGISPKIRWEIGAVLEELPEYMQICYLAMFN--------------FGNE 371

Cs2g22100.1 -----------------KRWDTG-AMEKLPYNMKICYLAMLN--------------FGND 392

Cs2g07280 ----------------VERWDFK-EIQRLPECMKLCFKALYD--------------TTNE 368

Cs2g24530.1 ----------------VERWDFK-EIQRLPECMKLCFKALYD--------------TTNE 350

Cs5g22980 ----------------VERWDINFAIKQLPDYMKICFFALYN--------------FVSE 391

Cs3g04340 ----------------VERWDINYALNHLPDYMKFCFLALYN--------------LVNE 267

BAD27256 ----------------VERWDINYALNHLPDYMKICFLALYN--------------LVNE 390

AAM53944 ----------------VERWDINYALKHLPGYMKMCFLALYN--------------FVNE 390

BAF73932 ----------------VERWDINYALKHLPGYMKMCFLALYN--------------FVNE 390

AAM53946 ----------------VARWDINYALKHLPGYMKMCFLALYN--------------FVNE 390

BAD27257 ----------------VARWDINYALKHLPGYMKMCFLALYN--------------FVNE 392

AOP12358 ----------------VARWDINYALKHLPGYMKMCFLALYN--------------FVNE 391

Cs3g04170 ----------------VARWDINYALKHLPGYMKMCFLALYN--------------FVNE 391

Cs3g04360 ----------------VARWDINYALKHLPGYMKMCFLALYN--------------FVNE 391

Cs3g04190 ----------------R---------------HPLCFEPPSG--------------LH-- 289

orange1.1t04775.1 ----------------VERWDINYALNHLPDYMKFCFLALYN--------------LVNE 362

orange1.1t04366 ----------------VERWDINYALNHLPDYMKLCFFALYN--------------FVNE 324

BAM29049 ----------------VARWDIHYALNHLPDYMKLCFFALYN--------------FVNE 390

Cs2g22150 FNNTTYLYYYSKLSGDVARWDIN-AVNDLPNYMKLSFLALYN--------------TINE 326

BAD27258 ----------------VERWDAT-ATEQLPYYMKLCFHALYN--------------SVNE 400

BAD27259 ----------------VERWDAT-ATEQLPYYMKLCFHALYN--------------SVNE 400

Cs3g07850 ----------------VERWDAT-ATEQLPYYMKLCFHALYN--------------SVNE 360

AAM53943 ----------------VERWDAT-TTEQLPYYMKLCFHALYN--------------SVNE 400

Cs7g18530 ----------------VERWDAT-AVEQLPHYMKLCFHALRN--------------SINE 400

Cs3g07920 --------------YYIVRWDAT-AVEQLPHYMKLCFHALRN--------------SINE 363

BAF73933 ----------------VERWDAT-AVEQLPHYMKLCFHALRN--------------SINE 400

AAM53945 ----------------VERWDAT-AVEQLPHYMKLCFHALRN--------------SINE 400

BAD27260 ----------------VERWDAT-VVEKLPHYMKLCFHALRN--------------SINE 400

BAD91045 ----------------VERWDAT-TIEQLPDYMKLCFHALHN--------------SINE 400

Cs3g07880 --------------------------------STLSFKLL-------------------- 322

Cs7g17640 ----------------VERGDAT-TIEQLPDYMKLCFHALHN--------------SINE 427

Cs2g03570 ----------------VERWDVN-AMDQLPYYMKLCFLVLHN--------------STNE 410

Cs8g14120.1 ----------------VERWDVN-AMDQLPYYMKMCFHVLHS--------------STNE 421

BAD91046 ----------------VERWDVN-AMDQLPYYMKLCFHVLHS--------------STNE 413

Cs5g15530 ----------------KRKIFVDEFRNCINPQ-----EGLASNKTEQGFTGTILRQHIYL 571

Cs5g31210 ----------------QRRAFVREFRNCISAWDYINRRRWDSNKKGERFAGKTLLGTLNH 656

Cs2g06470 ----------------VKRWDGK----GLSGHSKTIFGALDS--------------LVSE 576

orange1.1t03278 ----------------LERWDVEEAKNCCSEQVEIIFSALRS--------------TICE 574

Cs2g22180 ISYRILKDH-------------------GW--NVVP----QLKRT-----WIDIFEAQLS 403

Cs2g07250 ISYKVYKKH-------------------GY--NPVQ----SLRNA-----WTSLCKAFLV 417

BAP75559 ISCKVYKKH-------------------GY--NPVQ----SLRNA-----WTSLCKAFLV 415

Cs2g07240 ISNKVYKEH-------------------GY--NPVH----SLRKA-----WGSLCNAFLT 413

BAP75560 ISKQGVQRS-------------------MGITLCTP----LRKGV-----GEVLCNAFLI 408

orange1.1t02448 ISNKVYKEH-------------------GY--NPVH----SLRKA-----WGSLCNAFLI 406

orange1.1t03307 IRNKVYKEH-------------------GY--NPVH----SLRKA-----WGSLCNAFLI 406

orange1.1t02008 LNNELAEE--------------------GR-SYSVS----FTKDM-----MKGVVRAYFV 421

Cs4g11980 MEDKLVNK--------------------EP-LCCMY----YAKEA-----IKGLVRAYFV 395

orange1.1t04360.1 MEDKLVNK--------------------EP-LCCMY----YAKEA-----IKGLVRAYFV 403

orange1.1t04360.2 MEDKLVNK--------------------EP-LCCMY----YAKEA-----IKGLVRAYFV 396

Cs4g12080 AEEQISKE--------------------GR-TYCMS----YVIQA-----VKKVVQAYFE 387

Cs4g12050 AEEQISKE--------------------GR-G---------------------------- 368

Cs4g12060 ------------------------------------------------------------ 328

Cs5g12880 IEEDMAKQ--------------------GR-SHCVR----YAKEE-----NQKVIGAYFV 378

Cs5g12900.2 IEEDMAKQ--------------------GR-SHCVR----YAKEE-----NQKVIGAYSV 378

AAQ04608 IEEDMAKQ--------------------GR-SHCVR----YAKEE-----NQKVIGAYSV 378

Cs4g12350 VEQDLAKE--------------------GR-SSYLR----YDKEK-----MQELVQMYFV 386

Cs4g12400 IEQDLAKE--------------------GR-SSYLP----YGKEK-----MQELVQMYFV 386

Cs4g12220 CI-----------------------------IVDFD----YI-SQ-----FQELVMKYFC 316

Cs4g12480 ------------------------------------------------------------ 322

Cs4g11320 AKEELAKE--------------------GR-SHGIA----YAKQM-----MQELIILYFT 239

orange1.1t03302.1 AEEELAKE--------------------GR-SYGIP----YAKQM-----MQELIILYFT 315

Q94JS8 AEEELAKE--------------------GR-SYGIP----YAKQM-----MQELIILYFT 389

Cs4g12450 AEESLAKE--------------------GRSSYGVK----YVKQT-----MEESIMMYFS 383

orange1.1t00017.2 AEEELAKQ--------------------GR-SYGIP----YAKQT-----MQEVILMYFT 383

Cs5g06290 IEKEIANE--------------------GW-SYRVQ----HAKEA-----MKILVEGYYD 384

Cs3g21560 IEEELATK--------------------GW-SYRVH----YAKEE-----MKILVHGYHD 372

Cs3g21590c IEEELATK--------------------GW-SYRVH----YAKEE-----MKILVHGYHD 372

Cs4g12110.1 FEKDLASK--------------------GI-LYGLP----FAKES-----MKILVRSYII 394

BAP75561 FEKDLASK--------------------GI-LYGLP----FAKES-----MKILVRSYII 394

Cs3g16210 AEKDLAPQ--------------------GK-LYRLH----YAKKAVEYSSMKNIVKNCFF 719

Cs2g23470 AEKDLASQ--------------------GK-LYRLH----YAKEA-----MKNLVKHYLF 391

Cs2g24110.1 VEKDLASQ--------------------GK-LYRLH----YAKEA-----MKNLVKHYLF 292

Cs5g23510.1 VEKDLVSQ--------------------GK-LSRLH----YAKEA-----MKNQVKHYFF 390

Cs5g23540 VEKDLASQ--------------------AK-LYRLH----YAKEA-----MKNQVKHYFF 390

BAP74389 AEKDLASQ--------------------GK-LYHLH----YAKEA-----MKNQVKNYFF 393

Cs4g12090 AEKDLAPQ--------------------GK-SYRLY----YAKEA-----MKNMVKNYFY 393

CuSTS1 AEKDLAPQ--------------------GK-SYRLY----YAKEA-----MKNMVKNYFY 392

Cs4g12120.1 AEKDLAPQ--------------------GK-SYRLY----YAKEA-----MKNMVKNYFY 346

Cs4g12120.2 AEKDLAPQ--------------------GK-SYRLY----YAKEA-----MKNMVKNYFY 392

Cs2g22090 LACDVMKI--------------------HGLN-TLS----YIKKE-----WENLCTSYLV 401

Cs2g22100.1 LAYDILKN--------------------HGLN-FLS----YIKNE-----WANLCGSYLV 422

Cs2g07280 MAYEIGRR--------------------NTWKRVLP----HLKKE-----WSDFCKSLLV 399

Cs2g24530.1 MAYEIGRR--------------------NTWKQVLP----HLKKE-----WSDFCKSLLV 381

Cs5g22980 VAYYILKQ--------------------QDSDQLLRIKN-----S-----WLGLLQAFLV 421

Cs3g04340 FTYYVLKQ--------------------QGFDILRSIKNALRKIL-----WLRNIQAYLV 302

BAD27256 FTYYVLKQ--------------------QDFDILRSIKN-----A-----WLRNIQAYLV 420

AAM53944 FAYYVLKQ--------------------QDFDLLLSIKN-----A-----WLGLIQAYLV 420

BAF73932 FAYYVLKQ--------------------QDFDLLLSIKN-----A-----WLGLIQAYLV 420

AAM53946 FAYYVLKQ--------------------QDFDMLLSIKN-----A-----WLGLIQAYLV 420

BAD27257 FAYYVLKQ--------------------QDFDMLLSIKH-----A-----WLGLIQAYLV 422

AOP12358 FAYYVLKQ--------------------QDFDMLLSIKH-----A-----WLGLIQAYLV 421

Cs3g04170 FAYYVLKQ--------------------QDFDMLLSIKN-----A-----WLGLIQAYLV 421

Cs3g04360 FAYYVLKQ--------------------QDFDMLLSIKN-----A-----WLGLIQAYLV 421

Cs3g04190 -------------------------------------------EI-----WLGLLQACLV 301

orange1.1t04775.1 FTYYVLKQ--------------------QGFDILRSIKNALRKIL-----WLRNIQAYLV 397

orange1.1t04366 FAYYVLKQ--------------------QDFDMLRSIKN-----S-----WLGLLQACLV 354

BAM29049 FAYYVLKK--------------------QDFDMLRSIKN-----S-----WLGLLQACLV 420

Cs2g22150 MAYDILKQHISFPALYNTVNEMAYDILKQHGEIIIP----YLTKA-----WADLCKSFLQ 377

BAD27258 MGFIALRD--------------------QEVGMIIP----YLKKA-----WADQCKSYLV 431

BAD27259 MGFIALRD--------------------QEVGMIIP----YLKKA-----WADQCKSYLV 431

Cs3g07850 MGFIALRD--------------------QEVGMIIP----YLKKA-----WADQCKSYLV 391

AAM53943 MGFIALRD--------------------QEVGMIIP----YLKKA-----WADQCKSYLV 431

Cs7g18530 MTFDALRD--------------------QGVDIVIS----YLTKA-----WADICKAYLV 431

Cs3g07920 MTFDALRD--------------------QGVDIVIS----YLTKA-----WADICKAYLV 394

BAF73933 MTFDALRD--------------------QGVDIVIS----YLTKA-----WADICKAYLV 431

AAM53945 MTFDALRD--------------------QGVDIVIS----YLTKA-----WADICKAYLV 431

BAD27260 MTFDALRD--------------------QGVDIVIS----YLTKA-----WADICKAYLV 431

BAD91045 MAFDALRD--------------------QGVGMVIS----YLKKA-----WADICKTYLV 431

Cs3g07880 ------KP--------------------HGFK---------VFLD-----WADICKTYLV 342

Cs7g17640 MAFDALRD--------------------QGVGMVIS----YLKKA-----WADICKTYLV 458

Cs2g03570 MAFDVLKK--------------------QGVH-IIP----YLKKA-----WADMCKSFLL 440

Cs8g14120.1 MAFDALKD--------------------QGVH-VVP----YLKKA-----WADMCKSFLL 451

BAD91046 MAFDTLKD--------------------QGVH-VVP----YLKKA-----WADMCKSFLL 443

Cs5g15530 SFDPQ-PA--------------------QG-RNISH----QLLHA-----WEKWLMKWQA 600

Cs5g31210 IALDALVA--------------------NG-RDIGG----NLREA-----WEKWLTTWQD 686

Cs2g06470 LAEKHLQQ--------------------QG-RDITN----DLKDI-----WYETFASWLT 606

orange1.1t03278 FGDKTLTW--------------------QG-RNATS----HIVET-----WLNLLQSMFK 604

Cs2g22180 EAKWFSEGYVPTQEQYLRNGVTTGGTYMALVHAFCLMGQD----VTKE---TLAMMEPYP 456

Cs2g07250 EAKWFASGHMPESEEYLKNGIESSGVHVALVHIFFLLGHG----ITEE---TVELIDSNP 470

BAP75559 EAKWFASGHMPEAEEYLRNGIESSGVHVALAHFFFLLGHG----ITKE---TVELIDGNP 468

Cs2g07240 EAKWFASGQLPKAEEYLKNGIVSSGVHLGLVHIFFLLGHG----ITSE---TVRLIDSNP 466

BAP75560 EAKWFASGHLPKAEEYLENGIVSSGVHLVLVHIFFLLGHG----ITNE---TVQLIDSNP 461

orange1.1t02448 EAKWFASGHLPKAEEYLENGIVSSGVHLVLVHIFFLLGHG----VTNE---TVQLIDSNP 459

orange1.1t03307 EAKWFASGHLPKAEEYLENGIVSSGVHLVLVHIFFLLGHG----ITNE---TVQLIDSNP 459

orange1.1t02008 EAQWFREGYVPPFDERMSNAIVTGTCFLDPAAAYIGLGDI----AGID---AYEWLRSQP 474

Cs4g11980 EAKWFNAKYVPTFEEYMENSTMSSGYPMLAVEALVGLEDM---AITKQ---ALDWAISVP 449

orange1.1t04360.1 EAKWFHAKYVPTFEECVENSTMSSGYPMLAVEALVGLEDM---AITKR---ALDWAISVP 457

orange1.1t04360.2 EAKWFHAKYVPTFEECVENSTMSSGYPMLAVEALVGLEDM---AITKR---ALDWAISVP 450

Cs4g12080 EAKWCNEGYFPKVEEYMQVSLVTTCYHMLATASFLGMGKI----ADKL---AFERISNYP 440

Cs4g12050 ---------------------------------------I----ADKQ---AFEWISNYP 382

Cs4g12060 ------------------------------------------------------------ 328

Cs5g12880 QAKWFSEGYVPTIEEYMPIALTSCAYTFVITNSFLGMGDF----ATKE---VFEWISDNP 431

Cs5g12900.2 QAKWFSEGYVPTIEEYMPIALTSCAYTFVITNSFLGMGDF----ATKE---VFEWISNNP 431

AAQ04608 QAKWFSEGYVPTIEEYMPIALTSCAYTFVITNSFLGMGDF----ATKE---VFEWISNNP 431

Cs4g12350 QAKWSSEGYVPTWEEYYPVGLVSGGYFMLATNSFLGMCEV----ANKE---AFEWISKNP 439

Cs4g12400 QAKWFSEGYVPTWDEYYPVGLVSCGYFMLATNSFLGMCDV----ANKE---AFEWISKDP 439

Cs4g12220 EAKWLNEGYVPSMDEYKSVSLRSIDFLPIAVASFIFMGDI----ASRE---IFEWEMSNP 369

Cs4g12480 ------------------------------------------------------------ 322

Cs4g11320 QAKWLYKGYVPSFDEYKSVALRSIGLRTLGVASFVDLGDF---IATKD---NFEGILKNA 293

orange1.1t03302.1 EAKWLYKGYVPTFDEYKSVALRSIGLRTLAVASFVDLGDF---IATKD---NFECILKNA 369

Q94JS8 EAKWLYKGYVPTFDEYKSVALRSIGLRTLAVASFVDLGDF---IATKD---NFECILKNA 443

Cs4g12450 EAKWLHEGFLPKIEEYEGVALGSSGVLTLATASFVDMGDI----ATKE---AFEWLIKKP 436

orange1.1t00017.2 EAKWLKEGYVPSVEEYKSVALRSIAVLPVVTASFLDMGDI----ATKE---VFEWVVKVP 436

Cs5g06290 ESKWFHENYIPKMEEYMRVALVTSGYTMLTTVSFLGMDNI----VTKE---TFDWVFSRP 437

Cs3g21560 ESKWFHNNYVPTMEEYMRVSLVTSAYSMLTAASFLGMDSV----VTKE---AFDWVSEKP 425

Cs3g21590c ESKWFHNNYVPTMEEYMRVSLVTSGYSMLTAASFLGMDGV----VTKE---AFDWVSGKP 425

Cs4g12110.1 EARWCDQQYVPTMEEYMRVALLSCGYLLLSTSSFLGMEDI----VTKE---AFEWVSGNP 447

BAP75561 EARWCDQQYVPTMEEYMRVALLSCGYLLLSTSSFLGMEDI----VTKE---AFEWVSGNP 447

Cs3g16210 EAKWCHQNYIPTMDEYMTVALVTSAYPMLSTTSFVGMGDI----VTKE---SFEWLFSNP 772

Cs2g23470 EAKWCHQNYVPTVDEYMAVALITSASLMLSTISFVGMGDI----VTKE---SFEWLFSNP 444

Cs2g24110.1 EAKWCHQNYVPTVDEYMAVALITSASLMLSTISFVGMGDI----VTKE---SFEWLFSNP 345

Cs5g23510.1 EAKWYHQNYVPTVDEYMTVALISSAHPNLSTISFVGMGDI----VTKE---SFEWLFSNP 443

Cs5g23540 EAKWYHQNYIPTVDEYMTVALISSGHPNLSAISFVGMGDI----VTKE---SFEWLFSNP 443

BAP74389 EAKWCHQNYIPSVDEYMTVASVTSGYPMLSTTSFVGMGDI----VTKE---SFEWSLTNP 446

Cs4g12090 EAKWCLQNYVPTVDEYMTVALVTSGSPMLSTTSFVGMGDI----VTKE---SFEWLFSNP 446

CuSTS1 EAKWCLQNYVPTVDEYMTVALVTSGSPMLSTTSFVGMGDI----VTKE---SFEWLFSNP 445

Cs4g12120.1 EAKWCLQNYVPTVDEYMTVALVTSGSPMLSTTSFVGMGDI----VTKE---SFEWLFSNP 399

Cs4g12120.2 EAKWCLQNYVPTVDEYMTVALVTSGSPMLSTTSFVGMGDI----VTKE---SFEWLFSNP 445

Cs2g22090 EARWFSKGYTPTAKEYIENAWVSVGSPAAIVHAYILLQLQGSNALTENSLSCLKVEHGYD 461

Cs2g22100.1 EARWFSRGHKPTLKEYLGNAWTSVGGPAAIVHAYLLQAEE-CNLTEHS---LINCLKDGY 478

Cs2g07280 EAKWQKRGHTPCLQEYLSNAWISSSGTVLSVYSFFGIMKE----ATEE---TAGFLKLNQ 452

Cs2g24530.1 EAKWQKRGHTPCLQEYLSNAWISSSGTVLSVYSFFGIMKE----ATEE---TAGFLKLNQ 434

Cs5g22980 EAKWYHNKYAPTLEEYLKNAALSIAGPLITITAYLSATDP----IVEK---ELEYLESNP 474

Cs3g04340 EAKWYHGKYTPTLGEFLENGLVSIGGPMVTMTAYLSGTNP----IIEK---ELEFLESNQ 355

BAD27256 EAKWYHGKYTPTLGEFLENGLVSIGGPMVTMTAYLSGTNP----IIEK---ELEFLESNQ 473

AAM53944 EAKWYHSKYTPKLEEYLENGLVSITGPLIITISYLSGTNP----IIKK---ELEFLESNP 473

BAF73932 EAKWYHSKYTPKLEEYLENGLVSITGPLIITISYLSGTNP----IIKK---ELEFLESNP 473

AAM53946 EAKWYHSKYTPKLEEYLENGLVSITGPLIIAISYLSGTNP----IIKK---ELEFLESNP 473

BAD27257 EAKWYHSKYTPKLEEYLENGLVSITGPLIITISYLSGTNP----IIKK---ELEFLESNP 475

AOP12358 EAKWYHSKYTPKLEEYLENGLVSITGPLIITISYLSGTNP----IIKK---ELEFLESNP 474

Cs3g04170 EAKWYHSKYTPKLEEYLENGLVSITGPLIIAISYLSGTNP----IIKK---ELEFLESNP 474

Cs3g04360 EAKWYHSKYTPKLEEYLENGLVSITGPLIIAISYLSGTNP----IIKK---ELEFLESNP 474

Cs3g04190 EAKWYHSKYKPTLGEFLENGLVSIGGPMGTMTSYLSGTNP----IIEK---ELEFLESNQ 354

orange1.1t04775.1 EAKWYHSKYTPTLGEFLENGLVSIAGPIVTISAYLSGTNP----IIEK---ELEFLESNP 450

orange1.1t04366 EAKWYHSKYTPTLGEFLENGLVSIGGPMGIMTAYLSGTNP----IIEK---ELEFLESNQ 407

BAM29049 EAKWYHTKYTPTLGEFLENGLVSIGGPMGIMTAYLSGTNP----IIEK---ELEFLESNQ 473

Cs2g22150 EAKWSYNEYTPTFEEYLENAWRSSSGELSLVHSYFLVSRS----INKK---ALESLGKYH 430

BAD27258 EAKWYNSGYIPTLQEYMENAWISVTAPVMLLHAYAFTANP----ITKE---ALEFLQDSP 484

BAD27259 EAKWYNSGYIPTLQEYMENAWISVTAPVMLLHAYAFTANP----ITKE---ALEFLQDSP 484

Cs3g07850 EAKWYNSGYIPTLQEYMENAWISVTAPVMLIHAYAFTANP----ITKE---ALEFLQDSP 444

AAM53943 EAKWYNSGYIPTLQEYMENAWISVTAPVMLLHAYAFTANP----ITKE---ALEFLQDSP 484

Cs7g18530 EAKWYNSGYIPPLQEYMENAWISIGATVILVHANTFTANP----ITKE---GLEFVKDYP 484

Cs3g07920 EAKWYNSGYIPSLQEYMENAWISIGSTVILVHAYTFTANP----ITKE---GLEFVKDYP 447

BAF73933 EAKWYNSGYIPSLQEYMENAWISIGSTVILVHAYTFTANP----ITKE---GLEFVKDYP 484

AAM53945 EAKWYNSGYIPPLQEYMENAWISIGATVILVHANTFTANP----ITKE---GLEFVKDYP 484

BAD27260 EAKWYNSGYIPPLQEYMENAWISIGATVILVHANTFTANP----ITKE---GLEFVKDYP 484

BAD91045 EAKWYNNGYIPTLQEYMENAWISISAPVILVHAYTYTANP----ITKE---GLEFVKDYP 484

Cs3g07880 EAKWYNNGYIPTLQEYMENAWISISAPVILVHAYTYTANP----ITKE---GLEFVKDYP 395

Cs7g17640 EAKSYNNGYIPTLQEYMENAWISISAPVILVHAYTYTANP----ITKE---GLEFVKDYP 511

Cs2g03570 EAKWYSSGYIPTLEEYMDNAWISVSGPVILLHAYTLIANP----PTKE---ALQFLEEYP 493

Cs8g14120.1 EAKWYSSGYIPTLDEYIENAWVSVSGPVILLHAYSLIANP----AKEE---ALQFLQEYP 504

BAD91046 EAKWYSSGYIPTLDEYIENAWVSVSGPVILLHAYTLIANP----AKEE---ALQFLQEYP 496

Cs5g15530 QGDRHQGE-----------------AELIVQTINLSAGNC----LSED----LLSHQEYK 635

Cs5g31210 DGNSHWGV-----------------AELLVQTINLSAGRL----LSEE----LLCHPEYA 721

Cs2g06470 EATWSKSGRTPSMEEYLETGMISIAAHTLVLTASCFLNPS----LPNY----KFRPAQYD 658

orange1.1t03278 EAKCLRNKSVPTLDEYMENAYASFALGPIIFPAVYFVGPK----LSEE----VVRDPEFH 656

**DD**XX**T**XXX**E**

Cs2g22180 NLFSCSGKILRLW**DD**LG**T**ARE**E**QERGDVASSIECYMREKT----ISCEEEARKHIRQLIR 512

Cs2g07250 AIISSTATILRLW**DD**LG**S**AKD**E**NQDGKDGSYIHYYMKEHR----YSAAEEAQKSAINKIS 526

BAP75559 AIISSTATILRLW**DD**LG**S**AKD**E**NQEGKDGSYIHYYMKEHR----YSAAEEAQKSAINKIS 524

Cs2g07240 PIVSSVATILRLW**DD**LG**S**AKD**E**NQDGNDGSYIYYYMMEHQ----DVTAEDAQKHAMDKIS 522

BAP75560 PIVSSVATILRIW**DD**LG**S**AKD**E**NQGGKDGSYIYYYMMEHR----DLTAEDAHKHAMDKIS 517

orange1.1t02448 PIVSSVATILRIW**DD**LG**S**AKD**E**NQGGKDGSSIYYYMMEHR----DLTAEDAHKHAMDKIS 515

orange1.1t03307 LIVSSVAPILRIW**DD**LG**S**AKD**E**NQGGKDGSSIYYYMMEHR----DLTAEDAHKHAMDKIS 515

orange1.1t02008 KIMTASFTLSRLIA**D**LV**S**NKA**E**QERGHVASVVESYMKEYG-----TSGEETAEEFKKMIA 529

Cs4g11980 KIIRYSSLIARLD**DD**VR**T**YKV**E**QERGDAPSSVECYMQQYG-----VSEEEACNKIKEMVE 504

orange1.1t04360.1 KIIRSSSLIARLD**DD**VH**T**YKV**E**QERGDAPSSVECYVQQYG-----VSEEEACNKIKGMVE 512

orange1.1t04360.2 KIIRSSSLIARLD**DD**VH**T**YKV**E**QERGDAPSSVECYVQQYG-----VSEEEACNKIKGMVE 505

Cs4g12080 EIVKASEVICRLM**DD**IV**S**HEF**E**QKRKHVASGIECYMKQHG-----VSDEED--------- 486

Cs4g12050 KIVKASQVICRLM**DD**IV**S**HEF**E**QKRKHVASGIECYMKQHG-----VSDEEVIKVFRKEIS 437

Cs4g12060 --------IKRLM**DD**IV**S**HEF**E**QKRKHVASGIECYMKQHG-----VSDEEVIKVFRKEIS 375

Cs5g12880 KVVKAASVICRLL**DD**MQGHEF**E**QKRGHVASAIECYTKQHG-----VSKEEAIKMFEEEVA 486

Cs5g12900.2 KVVKAASVICRLM**DD**MQGHEF**E**QKRGHVASAIECYTKQHG-----VSKEEAIKMFEEEVA 486

AAQ04608 KVVKAASVICRLM**DD**MQGHEF**E**QKRGHVASAIECYTKQHG-----VSKEEAIKMFEEEVA 486

Cs4g12350 KISRASSVISRLM**ND**IV**S**HQF**E**QKRGHVTTGVECYCKQHG-----VSEEEVVKVFTEEVE 494

Cs4g12400 KISTASSVICRLR**ND**IV**S**QQF**E**QKRGHIASGVECYIKQYG-----VSEEEVVTVFTEEVE 494

Cs4g12220 DIIIAAETIIRFL**DD**IAGHKF**E**QKREHNPSAVECYKNQHG-----VSEEEAVKALSLAVA 424

Cs4g12480 ------EAVTRLM**DD**IAGYKF**E**QKRGHIPSAVECYKNQHG-----VSEEEAVKELLLEVA 371

Cs4g11320 KSVKATETIIRLM**DD**IAVYKF**E**QKRGHNPTAVECYKNQHG-----VSEEEAVKELLLEVA 348

orange1.1t03302.1 KSLKATETIGRLM**DD**IAGYKF**E**QKRGHNPSAVECYKNQHG-----VSEEEAVKELLLEVA 424

Q94JS8 KSLKATETIGRLM**DD**IAGYKF**E**QKRGHNPSAVECYKNQHG-----VSEEEAVKELLLEVA 498

Cs4g12450 KIVVAAQTIGRLM**DD**IA**S**HEF**E**QKRGHIPSAVECYMKQHG-----VSEEEAKKALRIQVD 491

orange1.1t00017.2 KIITASENICRLL**DD**VA**S**HKF**E**QKRGHIPSAVECYMKQHV-----VSEEEAEKALWLEIA 491

Cs5g06290 KIIRASEIIGRFM**DD**IK**S**HKF**E**QERGHAASAVECYMKQHG-----LSEQEVCEELYRQVS 492

Cs3g21560 RIIRASTIIGRLV**ND**IK**S**HKF**E**QERGHPASAIECYMKEKEREGVSVTQQEVHEELYKKVG 485

Cs3g21590c KIIRASTIIGRLM**DD**IK**S**HKF**E**QERGHAASAIECYMKEKEREGVSVTEQEVHEELYKKVG 485

Cs4g12110.1 KIVQASSIICRLM**DD**IV**S**HKF**E**QQRGHVASAVECYMKQHG-----VSEEEAVKVFREKVG 502

BAP75561 KIVQASSIICRLM**DD**IV**S**HKF**E**QQRGHVASAVECYMKQHG-----VSEEEAVKVFREKVG 502

Cs3g16210 RFIRASSVVCRLM**DD**MV**S**HKF**E**QSRGHVASSVECYMKQHG-----ATEEEACNEFRKQVS 827

Cs2g23470 RSIRASSVVNRLM**ND**IM**S**HKF**E**QSRGHVASSVECYMKQYE-----ATEEEAYNELRKQVS 499

Cs2g24110.1 RSIRASSVVNRLM**ND**IM**S**HKF**E**QSRGHVASSVECYMKQYE-----ATEEEAYNELRKQVS 400

Cs5g23510.1 RSIRASCAVGRLM**ND**MV**S**HKF**E**QSRGHVASSVECYMNQYG-----ATEEEAYSEFRKQVS 498

Cs5g23540 RSIRASCAVGRLM**ND**MA**S**HKF**E**QSRGHVASSVECYMNQYG-----ATEEEAYSEFRKQVS 498

BAP74389 RVIRASSVAARLM**ND**MV**S**HKF**E**QSRGHVASCVECYIKQYG-----ATEEEACNEFRKQVS 501

Cs4g12090 RFIRASSIVCRLM**DD**IV**S**HKF**E**QSRGHVASSVECYMKQHG-----ATEEEACNEFRKQVS 501

CuSTS1 RFIRASSIVYRLM**DD**IV**S**HKF**E**QSRGHVASSVECYIKQHG-----ATEEEACNEFRKQVS 500

Cs4g12120.1 RFIRASSIVCRLM**DD**IV**S**HKF**E**QSRGHVASSVECYMKQHG-----ATEEEACNEFRKQVS 454

Cs4g12120.2 RFIRASSIVCRLM**DD**IV**S**HKF**E**QSRGHVASSVECYMKQHG-----ATEEEACNEFRKQVS 500

Cs2g22090 EMIYWSSLISRLS**ND**LGNSAV**E**LKRGDVAKSIQCYMIEEG-----ISEEEARDRIKSLII 516

Cs2g22100.1 EIIYWSSLIARLS**ND**LG**T**SMA**E**IKRGDVAKSVQCCMNEEG-----ISAEEARERIQGLLN 533

Cs2g07280 DLVYNSSLIIRLC**ND**LG**T**SSA**E**LERGDVASSILCCMTEMN-----ISEEIARNYIKGMIS 507

Cs2g24530.1 DLVYNSSLIIRLC**ND**LG**T**SSA**E**LERGDVASSILCCMTEMN-----ISEEIARNYIKGMIS 489

Cs5g22980 DVIQWSSRIFRLL**DD**LG**T**SSD**E**IQRGDVSKSIQCYMHETG-----ASEEAAREHIKNLIR 529

Cs3g04340 DIVHWSFKILRLQ**DD**LG**T**SSD**E**IRRGDVPKSIQCYMHETG-----ASEEVAREHIKDMMR 410

BAD27256 DISHWSFKILRLQ**DD**LG**T**SSD**E**IRRGDVPKSIQCYMHETG-----ASEEVAREHIKDMMR 528

AAM53944 DIVHWSSKIFRLQ**DD**LG**T**SSD**E**IQRGDVPKSIQCYMHETG-----ASEEVARQHIKDMMR 528

BAF73932 DIVHWSSKIFRLQ**DD**LG**T**SSD**E**IQRGDVPKSIQCYMHETG-----ASEEVARQHIKDMMR 528

AAM53946 DIVHWSSKIFRLQ**DD**LG**T**SSD**E**IQRGDVPKSIQCYMHETG-----ASEEVAREHIKDMMR 528

BAD27257 DIVHWSSKIFRLQ**DD**LG**T**SSD**E**IQRGDVPKSIQCYMHETG-----ASEEVAREHIKDMMR 530

AOP12358 DIVHWSSKIFRLQ**DD**LG**T**SSD**E**IQRGDVPKSIQCYMHETG-----ASEEVAREHIKDMMR 529

Cs3g04170 DIVHWSSKIFRLQ**DD**LG**T**SSD**E**IQRGDVPKSIQCYMHETG-----ASEEVAREHIKDMMR 529

Cs3g04360 DIVHWSSKIFRLQ**DD**LG**T**SSD**E**IQRGDVPKSIQCYMHETG-----ASEEVAREHIKDMMR 529

Cs3g04190 DIIHWSFKILRLQ**DD**LG**T**SSD**E**IQRGDVPKSIQCYMHETG-----ASEEVAREHIEDMMR 409

orange1.1t04775.1 DLVHWSCKIFRLQ**DD**LG**T**SSD**E**IQRGDVPKSIECYMHETG-----ASEEVAREHIKDMMR 505

orange1.1t04366 DIIHWSCKIFRLQ**DD**LG**T**SSD**E**IQRGDVPKSIECYMHETG-----ASEEVAREHIKDMMR 462

BAM29049 DIIHWSCKIFRLQ**DD**LG**T**SSD**E**IQRGDVPKSIECYMHETG-----ASEEVAREHIKDMMR 528

Cs2g22150 NLIRWPSTIFRLS**ND**LA**T**SKH--------------------------------------- 451

BAD27258 DIIRISSMIVRLE**DD**LG**T**SSD**E**LKRGDVPKSIQCYMHETG-----VSEDEAREHIRDLIA 539

BAD27259 DIIRISSMIVRLE**DD**LG**T**SSD**E**LKRGDVPKSIQCYMHETG-----VSEDEAREHIRDLIA 539

Cs3g07850 DIIRISSMIVRLE**DD**LG**T**SSD**E**LKRGDVPKSIQCYMHETR-----VSEDEAREHIRDLIA 499

AAM53943 DIIRISSMIVRLE**DD**LG**T**SSD**E**LKRGDVPKSIQCYMHETG-----VSEDEAREHIRDLIA 539

Cs7g18530 NIIRWSSVILRFA**DD**LG**T**SSVQLILF---------------------------------- 510

Cs3g07920 NIIRWSSVILRFA**DD**LG**T**SSD**E**LKRGDVHKSIQCYMHEAG-----VSEGEAREHINDLIA 502

BAF73933 NIIRWSSVILRFA**DD**LG**T**SSD**E**LKRGDVHKSIQCYMHEAG-----VSEGEAREHINDLIA 539

AAM53945 NIIRWSSMILRFA**DD**LG**T**SSD**E**LKRGDVHKSIQCYMHEAG-----VSEGEAREHINDLIA 539

BAD27260 NIIRWSSVILRFA**DD**LG**T**SSD**E**LKRGDVHKSIQCYMHEAG-----VSEGEAREHINDLIA 539

BAD91045 NIIRWSSIILRLA**DD**LG**T**SSD**E**LKRGDVHKSIQCYMHEAG-----VSEREAREHIHDLIA 539

Cs3g07880 NIIRWSSIILRLA**DD**LG**T**SLD**E**LKRGDVHKSIQCYMHEAG-----VSEREAREHIHDLIA 450

Cs7g17640 NIIRWSSIILRLA**DD**LG**T**SLD**E**LKRGDVHKSIQCYMHEAG-----VSEREAREHIHDLIA 566

Cs2g03570 NIIRWPSMIFRLA**ND**LA**T**SSD**E**VKRGDVPKAIQCYMHETG-----ASESDAREHIRDLIT 548

Cs8g14120.1 HIIRWPSMIFRLA**ND**LA**T**SSD**E**VKRGDVPKAIQCYMHETG-----ASESDARQHIRDLIT 559

BAD91046 HIIRWPSMIFRLA**ND**LA**T**SSD**E**VKRGDVPKAIQCYMHETG-----ASESDARLHIRDLIT 551

Cs5g15530 RLMDLTNTICHKLGQYQNQKVHDASDSDVDI-----------------------DHMQID 672

Cs5g31210 RLSYLTNRICHTLGHYHKQKVQVNRNNNIGNN--------------------RITALSVE 761

Cs2g06470 TVTKLLMLVPRLL**ND**IQ**S**YQK**E**IADGKQNSVI-LYLRENP----EADIEDSVAYVRELIA 713

orange1.1t03278 NLYKLVSTCGRLL**ND**IQGFKR**E**SKEGKLNSVSLQMTYSDG----NMTEVEAIEKIKVVIK 712

: .

Cs2g22180 SLWVELNG-MLVAPA----ALPASIVNASLNLARTAQVVYQHG-DDNTVTSV-------- 558

Cs2g07250 DAWKRLNK-ECLCPN----PFSASFTRASLNLARMVPLMYSYD-DNQRLPSL-------- 572

BAP75559 DAWKRLNK-ECLCPN----PFSASFTRASLNLARMVPLMYSYD-DNQRLPSL-------- 570

Cs2g07240 DAWKRLNQ-ECLSPN----PFSASFTRASLNIARMVPLMYSYD-DNQRLPRL-------- 568

BAP75560 DAWKRLNK-ECLSPN----PFSASFTRASFNCARMVPLMYSYD-DSQRLPSL-------- 563

orange1.1t02448 DAWKRLNK-ECLSPN----PFSASFTRASLNSARMVPLMYSYD-DSQRLPSL-------- 561

orange1.1t03307 DAWKRLNK-ECLSPN----PFSASFTRASLNSARMVPLMYSYD-DSQRLPSL-------- 561

orange1.1t02008 DGWKDINE-ECMRPT----IVPMRLLNVIVNIARLVEVFYKKM-DGVTNPE-YL------ 576

Cs4g11980 IEWMNINE-EIQDPN----HPPLQWLLPSLNLARMMVVLYQNG-DGYTNSSGKT------ 552

orange1.1t04360.1 IEWMNINE-EIQDPN----HPPLQWLLPSLNLARMMVVLYQNG-DGYTNSTGKT------ 560

orange1.1t04360.2 IEWMNINE-EIQDPN----HPPLQWLLPSLNLARMMVVLYQNG-DGYTNSTGKT------ 553

Cs4g12080 ------------------------------------------D-DGYTNSY-VI------ 496

Cs4g12050 NGWKDINE-GFLKPT----EVAMPLLERILNLARVMDVIYKDD-DGYTNSY-VI------ 484

Cs4g12060 NGWKDINE-GFLKPT----EVAMPLLERILNLARVMDVIYKDD-DGYTNSY-VI------ 422

Cs5g12880 NAWKDINEELMMKPT----VVARPLLGTILNLARAIDFIYKED-DGYTHSY-LI------ 534

Cs5g12900.2 NAWKDINEELMMKPT----VVARPLLGTILNLARAIDFIYKED-DGYTHSY-LI------ 534

AAQ04608 NAWKDINEELMMKPT----VVARPLLGTILNLARAIDFIYKED-DGYTHSY-LI------ 534

Cs4g12350 NAWKDMNE-EFLRPT----AFPVALIERPFNIARVLEFLYKKG-DCYTHSH-AI------ 541

Cs4g12400 NAWKDMNE-EFLKPT----AFPVALIERPFNIARVIEFLNKKG-DWYTHSH-AI------ 541

Cs4g12220 NSWKVINEELLLNPT----AIPLPLLQVIFDLSCSADFMYGDAQDRLTHST-MM------ 473

Cs4g12480 DSWKDINE-ELLNPT----TVPLPMLQRILYFARSGHLMYDDGHDRFTHSL-MM------ 419

Cs4g11320 DSWKDINE-ELLNPT----TVPLPMLQRILYFARSGHFIYDDGHDRYTHSL-MM------ 396

orange1.1t03302.1 NSWKDINE-ELLNPT----TVPLPMLQRLLYFARSGHFIYDDGHDRYTHSL-MM------ 472

Q94JS8 NSWKDINE-ELLNPT----TVPLPMLQRLLYFARSGHFIYDDGHDRYTHSL-MM------ 546

Cs4g12450 NAWKDINE-ELLSPT----AVSLPLLERILNLARVCHFMYEDG-DRYTQPL-LM------ 538

orange1.1t00017.2 NGWKDLNYEELLNLI----AMPLPLLGPVLNLARMSEFIYEDGVDRYTNSY-KM------ 540

Cs5g06290 NAWKDINE-ECLNPT----AVPMPLLMRALNLARVIDVVYKEG-DGYTHVGNEM------ 540

Cs3g21560 DAWKDINE-ECLMPT----EVPRALLMRVLNLSRVIDIIYKEA-DDYTHVGQVM------ 533

Cs3g21590c DAWKDINE-ECLMPT----EVPRPLLMRVLNLSRVIDVIYKEG-DGYTHVGQVM------ 533

Cs4g12110.1 NAWKDINE-ELMRPP----VVPMPLLERVLNLARLMDVLYQNN-DSYTNPH-LM------ 549

BAP75561 NAWKDINE-ELMRPP----VVPMPLLERVLNLARLMDVLYQNN-DSYTNPH-LM------ 549

Cs3g16210 NAWKDINE-DCLRPT----VVPMPLLMRILNLTRVIDVIYKYE-DGYTHSAVVL------ 875

Cs2g23470 NAWKDINE-DCLRPT----VVPMPLLMRILNLTRDADVTYKYD-DGYTFAE-VL------ 546

Cs2g24110.1 NAWKDINE-DCLRPT----VVPMPLLMRILNLTRDADVTYKYD-DGYTFAE-VL------ 447

Cs5g23510.1 NAWKDINE-ECLRPT----LVPVPLLMRILNLTRAADVVYKYK-DGYTDTE-EL------ 545

Cs5g23540 NAWKDINE-ECLRPT----VVPVPLLIRILNLTRAADVIYKYK-DGYTDSE-EL------ 545

BAP74389 NAWKDVNE-ECLRPT----VVPMPLLMRILNLTRFLDVVYRFE-DGYTHSGVVL------ 549

Cs4g12090 NAWKDINE-DCLRPT----VLPMPLLMRILNLTRVIDVIYKYE-DGYTHSAVVL------ 549

CuSTS1 NAWKDINE-DCLRPT----VVPMPLLMRILNLTRVIDVIYKYE-DGYTHSAVVL------ 548

Cs4g12120.1 NAWKDINE-DCLRPT----VVPMPLLMRILNLTRVIDVIYKYE-DGYTHSAVVL------ 502

Cs4g12120.2 NAWKDINE-DCLRPT----VVPMPLLMRILNLTRVIDVIYKYE-DGYTHSAVVL------ 548

Cs2g22090 YSWKKLNGKNLYKS-----DFPESMAKMCLDMSRTAHCIFH-G-DGIGTSTGVS------ 563

Cs2g22100.1 YSWKKLNENRIAKSN----CLPNTMVNMCLNMARTAQCIYQHG-DGIGSSEGVT------ 582

Cs2g07280 KTWTKINGQCFT-----QSPLLQSFIHITTNFARVVHSLYQYG-DGFGVQDGDT------ 555

Cs2g24530.1 KTWTKINGQCFT-----QSPLLQLFIHITTNFARVVHSLYQYG-DGFGVQDRDT------ 537

Cs5g22980 QMWKKVMMDVSRASNNKDSPLSQITNEFILNLVRVSHFMYLHG-DGHGVQNQET------ 582

Cs3g04340 QMWKKVNAY----RADKDSPLSQTTVEFILNVVRVSHFMYLHG-DGHGAQNQET------ 459

BAD27256 QMWKKVNAY----RADKDFPLSQTTVEFILNVVRVSHFMYLHG-DGHGAQNQET------ 577

AAM53944 QMWKKVNAY----TADKDSPLTGTTTEFLLNLVRMSHFMYLHG-DGHGVQNQET------ 577

BAF73932 QMWKKVNAY----TADKDSPLTGTTTEFLLNLVRMSHFMYLHG-DGHGVQNQET------ 577

AAM53946 QMWKKVNAY----TADKDSPLTRTTTEFLLNLVRMSHFMYLHG-DGHGVQNQET------ 577

BAD27257 QMWKKVNAY----TADKDSPLTRTTAEFLLNLVRMSHFMYLHG-DGHGVQNQET------ 579

AOP12358 QMWKKVNAY----TADKDSPLTRTTAEFLLNLVRMSHFMYLHG-DGHGVQNQET------ 578

Cs3g04170 QMWKKVNAY----TADKDSPLTRTTTEFLLNLVRMSHFMYLHG-DGHGVQNQET------ 578

Cs3g04360 QMWKKVNAY----TADKDSPLTRTTTEFLLNLVRMSHFMYLHG-DGHGVQNQET------ 578

Cs3g04190 QMWKKVNAY----RADKDSPLSQNTVDFMLNLVRMSHFMYLRR-DGHGAQNQET------ 458

orange1.1t04775.1 QMWKKVNAY----RADKDSPLSQNTVDFMLNLVRMSHFMYLRG-DGHGAQNQET------ 554

orange1.1t04366 QMWKKVNAY----RADKDSPLSQNTVDFMLNLVRMSHFMYLR--DGHGAQNQET------ 510

BAM29049 QMWKKVNAY----RADKDSPLSQNTVDFMLNLVRMSHFMYLRG-DGHGAQNQET------ 577

Cs2g22150 -------------------PFVEPFVETAFNLARIAQCTYQYG-DEHGAPDGRA------ 485

BAD27258 ETWMKMNSARFGNPP----YLPDVFIGIAMNLVRMSQCMYLYG-DGHG--VQEN------ 586

BAD27259 ETWMKMNSARFGNPP----YLPDVFIGIAMNLVRMSQCMYLYG-DGHG--VQEN------ 586

Cs3g07850 ETWMKMNSARFGNPP----YLPDVFIGIAMNLVRMSQCMYLYG-DGHG--VQEN------ 546

AAM53943 ETWMKMNSARFGNPP----YLPDVFIGIAMNLVRMSQCMYLYG-DGHG--VQEN------ 586

Cs7g18530 ------------------------------------------------------------ 510

Cs3g07920 QTWMKMNRDRFGNPH----FVSDVFVGIAMNLARMSQCMYQFG-DGHGCGAQEI------ 551

BAF73933 QTWMKMNRDRFGNPH----FVSDVFVGIAMNLARMSQCMYQFG-DGHGCGAQEI------ 588

AAM53945 QTWMKMNRDRFGNPH----FVSDVFVGIAMNLARMSQCMYQFG-DGHGCGAQEI------ 588

BAD27260 QTWMKINRDRFGNPH----FVSDVFVGIAMNLARMSQCMYQFG-DGHGCGAQEI------ 588

BAD91045 QTWMKMNRDRFGNPH----FVSDVFVGIAMNLARMSQCMYQFG-DGHGHGVQEI------ 588

Cs3g07880 QTWMKMNSDQFGNPH----FVSDVFVGIAMNLARMSQCMYQFG-DGHGHGVQEI------ 499

Cs7g17640 QTWMKMNSDQFGNPH----FVSDVFVGIAMNLARMSQCMYQFG-DGHGHGVQEI------ 615

Cs2g03570 ATWMKMNS-KDGDENPDHLLLSNNFIRFAINLARMAQCTYQNG-DGHT--IQHK------ 598

Cs8g14120.1 AAWMKMNNKREGDENPDHLLLPNNFVQFAMNLARMAQCTYQNG-DGHT--VQDN------ 610

BAD91046 AAWMKMNNKREGDENPDHLLLPNNFVQFAMNLARMAQCTYQNG-DRHT--VQDN------ 602

Cs5g15530 FAMQELVQVVLQSSS---NGIDFEVKQTFLTVTKSF-----YY-SAYCDSKTINFHIDKV 723

Cs5g31210 PDMQELVQLVLQNSS---SGIGSDVKQTFLTVAKSF-----YY-CAYCDAETINSHIGKV 812

Cs2g06470 TKEKELLEHALMDGF---SDLPRPCKQLHLFCMKAFQMFFHSS-NRYDSNTEMIDDVQKA 769

orange1.1t03278 STRRELLRLVLKEEG---SIVPRACKDLIWKMSKVLHLFYMNT-DGFSSNVEMVKAVSEV 768

Cs2g22180 ---------NGHIERLFCNPLTEQGHIREKTYE---IWFKNYTYFRDLSCILIHIEEGCM 606

Cs2g07250 ---------EHFIKSLLSESVPTEGVY--------------------------------- 590

BAP75559 ---------EHYIKSLLFESVPTEGVY--------------------------------- 588

Cs2g07240 ---------EEYIKSLLFDNLPTQGVY--------------------------------- 586

BAP75560 ---------EEYIKSSLFDNLPTQGVY--------------------------------- 581

orange1.1t02448 ---------EEYIKSFLFDNLPTQGVY--------------------------------- 579

orange1.1t03307 ---------EEYIKSSLFDNLPTQGVY--------------------------------- 579

orange1.1t02008 ---------KDHVTKLFIDPILV------------------------------------- 590

Cs4g11980 ---------KDRIASLLVDPLPM------------------------------------- 566

orange1.1t04360.1 ---------KDRIASLLVDPLPM------------------------------------- 574

orange1.1t04360.2 ---------KDRIASLLVDPLPM------------------------------------- 567

Cs4g12080 ---------KDYITTLLEKPVPF------------------------------------- 510

Cs4g12050 ---------KDYITTLLEKPVPF------------------------------------- 498

Cs4g12060 ---------KDYITTLLEKPVPF------------------------------------- 436

Cs5g12880 ---------KDQIASVLGDHVPF------------------------------------- 548

Cs5g12900.2 ---------KDQIASVLGDHVPF------------------------------------- 548

AAQ04608 ---------KDQIASVLGDHVPF------------------------------------- 548

Cs4g12350 ---------KDQIAAVLRDPVTI------------------------------------- 555

Cs4g12400 ---------KDQIAAVLRDPVTI------------------------------------- 555

Cs4g12220 ---------KDQVELVLRDPIKL------------------------------------- 487

Cs4g12480 ---------KRQVALLLAEPLAI------------------------------------- 433

Cs4g11320 ---------KRQVALLLTEPLAI------------------------------------- 410

orange1.1t03302.1 ---------KRQVALLLTEPLAI------------------------------------- 486

Q94JS8 ---------KRQVALLLTEPLAI------------------------------------- 560

Cs4g12450 ---------KDQVALVLKDPVTL------------------------------------- 552

orange1.1t00017.2 ---------KDQVALVLKDPVTF------------------------------------- 554

Cs5g06290 ---------KQNVAALLIDQVPI------------------------------------- 554

Cs3g21560 ---------KDNIASVLIHPVAI------------------------------------- 547

Cs3g21590c ---------KDNIASVLIHPVAI------------------------------------- 547

Cs4g12110.1 ---------KDHVAALLKDPVFFED----------------------------------- 565

BAP75561 ---------KDHVAALLKDPVFFED----------------------------------- 565

Cs3g16210 ---------N-------------------------------------------------- 876

Cs2g23470 ---------KDFIASLFINPVPISA----------------------------------- 562

Cs2g24110.1 ---------KDFIASLFINPVPISA----------------------------------- 463

Cs5g23510.1 ---------KDFIASLLINPVPI------------------------------------- 559

Cs5g23540 ---------KDFIVSLLINPVPI------------------------------------- 559

BAP74389 ---------KDFVASLLINPVSI------------------------------------- 563

Cs4g12090 ---------KDFVASLLINPVPI------------------------------------- 563

CuSTS1 ---------KDFVASLFINPVPI------------------------------------- 562

Cs4g12120.1 ---------KDFVASLFINPVPI------------------------------------- 516

Cs4g12120.2 ---------KDFVASLFINPVPI------------------------------------- 562

Cs2g22090 ---------RDRLVSLILEPIPVEL----------------------------------- 579

Cs2g22100.1 ---------KDRLVSLILEPIPIEQ----------------------------------- 598

Cs2g07280 ---------KKQILSLLIEPMPPSPFS--------------------------------- 573

Cs2g24530.1 ---------KKQILSLLIEPMPPSPFSSFIFPQNIIM----------------------- 565

Cs5g22980 ---------MDEAFALLFQPIPLEDNKHMAFTSA-------------------------- 607

Cs3g04340 ---------MDVVFTLLFQPIPLDDK-HVVATSSPGTKG--------------------- 488

BAD27256 ---------MDVVFTLLFQPIPLDDK-HIVATSSPVTKG--------------------- 606

AAM53944 ---------IDVGFTLLFQPIPLEDK-HMAFTASPGTKG--------------------- 606

BAF73932 ---------IDVGFTLLFQPIPLEDK-HMAFTASPGTKG--------------------- 606

AAM53946 ---------IDVGFTLLFQPIPLEDK-DMAFTASPGTKG--------------------- 606

BAD27257 ---------IDVGFTLLFQPIPLEDK-DMAFTASPGTKG--------------------- 608

AOP12358 ---------IDVGFTLLFQPIPLEDK-DMAFT---------------------------- 600

Cs3g04170 ---------IDVGFTLLFQPIPLEDK-DMAFTASPGTKG--------------------- 607

Cs3g04360 ---------IDVGFTLLFQPIPLEDK-DMAFTASPGTKG--------------------- 607

Cs3g04190 ---------MDVASTWLFQPIPLEDN-HMAFTASPGTKG--------------------- 487

orange1.1t04775.1 ---------MDVASTWLFQPIPLEDK-HMAFTAPKADEFPEYSFS--------------- 589

orange1.1t04366 ---------MDVASTWLFQPIPLEDN-HMAFTAPTADEFPECSFS--------------- 545

BAM29049 ---------MDVASTWLFQPIPLEDK-HMAFTAPKADEFPEYSFS--------------- 612

Cs2g22150 --------KKRVVISC-------------------------------------------- 493

BAD27258 --------TKDRVLSLFIDPIP-------------------------------------- 600

BAD27259 --------TKDRVLSLFIDPIP-------------------------------------- 600

Cs3g07850 --------TKDRVLSLFIDPIP-------------------------------------- 560

AAM53943 --------TKDRVLSLFIDPI--------------------------------------- 599

Cs7g18530 ------------------------------------------------------------ 510

Cs3g07920 --------TKARVLSLFIDPIA-------------------------------------- 565

BAF73933 --------TKARVLSLFIDPIA-------------------------------------- 602

AAM53945 --------TKARVLSLFFDPIA-------------------------------------- 602

BAD27260 --------TKARVLSLFFDPIA-------------------------------------- 602

BAD91045 --------TKARVLSLIVDPIA-------------------------------------- 602

Cs3g07880 --------TKARVLSLIVDPIA-------------------------------------- 513

Cs7g17640 --------TKARVLSLIVDPIA-------------------------------------- 629

Cs2g03570 --------SKNRVLPLLVHPVSLINL---------------------------------- 616

Cs8g14120.1 --------SKNRVLPLLIHPIKS------------------------------------- 625

BAD91046 --------SKNRVLPLLIHPIKS------------------------------------- 617

Cs5g15530 LFERVE------------------------------------------------------ 729

Cs5g31210 LFQTVDI----------------------------------------------------- 819

Cs2g06470 FYIPLKLQTPKPLIKLMLNQLPLPLHHHSR---------SKNDYQSVANCFNIYGKRSLA 820

orange1.1t03278 VYEPISVNM--------------------------------------------------- 777

Cs2g22180 YGE---------------------------- 609

Cs2g07250 ------------------------------- 590

BAP75559 ------------------------------- 588

Cs2g07240 ------------------------------- 586

BAP75560 ------------------------------- 581

orange1.1t02448 ------------------------------- 579

orange1.1t03307 ------------------------------- 579

orange1.1t02008 ------------------------------- 590

Cs4g11980 ------------------------------- 566

orange1.1t04360.1 ------------------------------- 574

orange1.1t04360.2 ------------------------------- 567

Cs4g12080 ------------------------------- 510

Cs4g12050 ------------------------------- 498

Cs4g12060 ------------------------------- 436

Cs5g12880 ------------------------------- 548

Cs5g12900.2 ------------------------------- 548

AAQ04608 ------------------------------- 548

Cs4g12350 ------------------------------- 555

Cs4g12400 ------------------------------- 555

Cs4g12220 ------------------------------- 487

Cs4g12480 ------------------------------- 433

Cs4g11320 ------------------------------- 410

orange1.1t03302.1 ------------------------------- 486

Q94JS8 ------------------------------- 560

Cs4g12450 ------------------------------- 552

orange1.1t00017.2 ------------------------------- 554

Cs5g06290 ------------------------------- 554

Cs3g21560 ------------------------------- 547

Cs3g21590c ------------------------------- 547

Cs4g12110.1 ------------------------------- 565

BAP75561 ------------------------------- 565

Cs3g16210 ------------------------------- 876

Cs2g23470 ------------------------------- 562

Cs2g24110.1 ------------------------------- 463

Cs5g23510.1 ------------------------------- 559

Cs5g23540 ------------------------------- 559

BAP74389 ------------------------------- 563

Cs4g12090 ------------------------------- 563

CuSTS1 ------------------------------- 562

Cs4g12120.1 ------------------------------- 516

Cs4g12120.2 ------------------------------- 562

Cs2g22090 ------------------------------- 579

Cs2g22100.1 ------------------------------- 598

Cs2g07280 ------------------------------- 573

Cs2g24530.1 ------------------------------- 565

Cs5g22980 ------------------------------- 607

Cs3g04340 ------------------------------- 488

BAD27256 ------------------------------- 606

AAM53944 ------------------------------- 606

BAF73932 ------------------------------- 606

AAM53946 ------------------------------- 606

BAD27257 ------------------------------- 608

AOP12358 ------------------------------- 600

Cs3g04170 ------------------------------- 607

Cs3g04360 ------------------------------- 607

Cs3g04190 ------------------------------- 487

orange1.1t04775.1 ------------------------------- 589

orange1.1t04366 ------------------------------- 545

BAM29049 ------------------------------- 612

Cs2g22150 ------------------------------- 493

BAD27258 ------------------------------- 600

BAD27259 ------------------------------- 600

Cs3g07850 ------------------------------- 560

AAM53943 ------------------------------- 599

Cs7g18530 ------------------------------- 510

Cs3g07920 ------------------------------- 565

BAF73933 ------------------------------- 602

AAM53945 ------------------------------- 602

BAD27260 ------------------------------- 602

BAD91045 ------------------------------- 602

Cs3g07880 ------------------------------- 513

Cs7g17640 ------------------------------- 629

Cs2g03570 ------------------------------- 616

Cs8g14120.1 ------------------------------- 625

BAD91046 ------------------------------- 617

Cs5g15530 ------------------------------- 729

Cs5g31210 ------------------------------- 819

Cs2g06470 APRVRWPVSRNFGYKNNIVMGVPPKIRFSFF 851

orange1.1t03278 ------------------------------- 777
