# Supplementary figures and images for: Genomic Analysis of Terpene Synthase Family and Functional Characterization of Seven Sesquiterpene Synthases from Citrus sinensis
Source: Front Plant Sci. 2017 Aug 24;8:1481. doi: 10.3389/fpls.2017.01481 (PMC5573811; doi:10.3389/fpls.2017.01481)

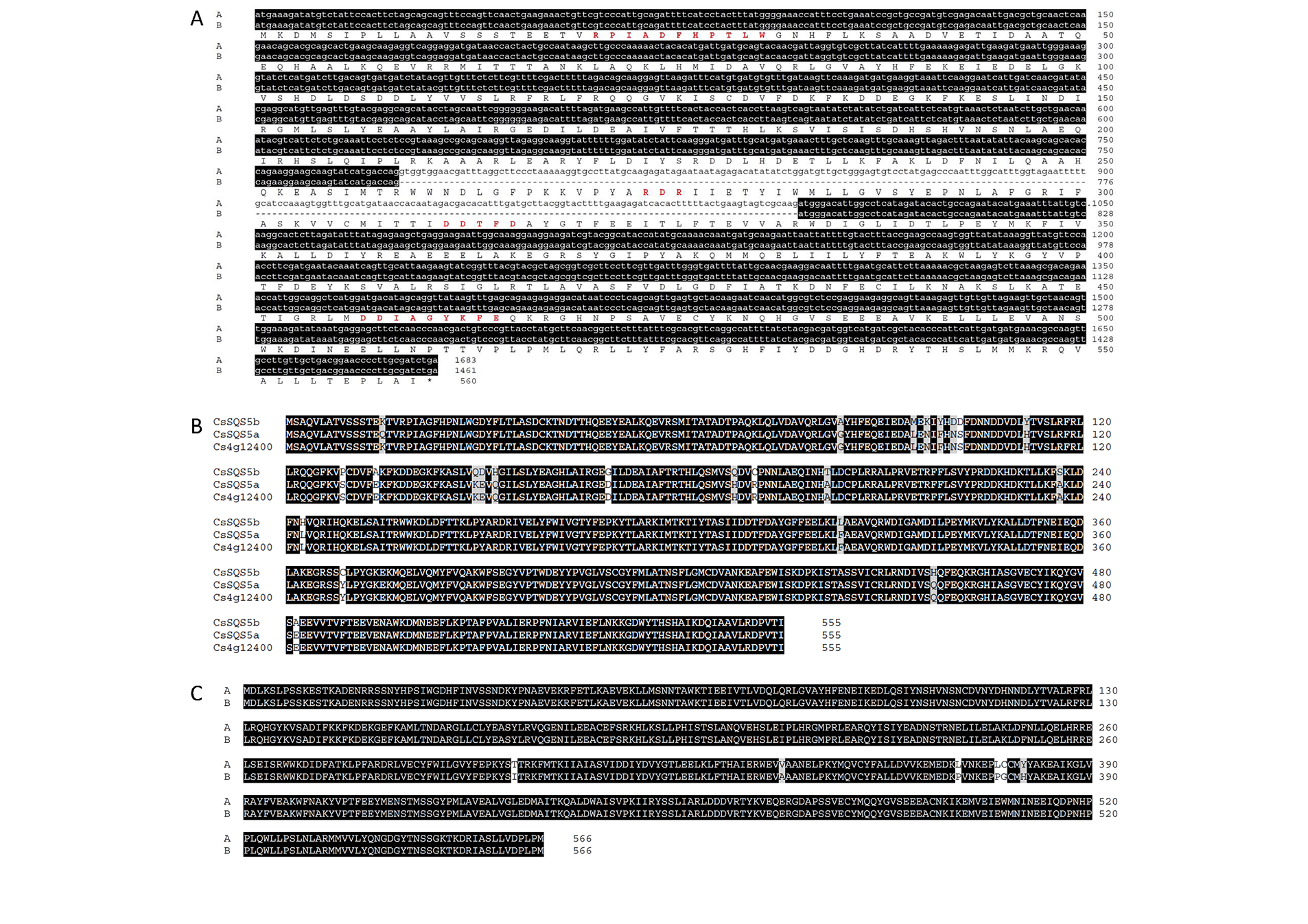

Supplement: Supplementary Figure 2 — Comparison of amino acid sequences between predicted transcripts in the CAP database and those obtained in this work. (A). Comparison of nucleotide sequences (lowercases) of orange1.1t03302 predicted transcript in the CAP database (A) and those obtained in this work (B). Identical nucleotides are white lettered on a black frame. Deduced amino acid for each triplet are detailed in capital letters. Characteristic TPS signatures are red-lettered. (B) Alignment of the deduced amino acid sequences of Cs4g12400 annotated in the CAP database (Cs4g12400) and from cDNAs cloned in this work (CsSesquiTPS5 (A,B). Identical amino acids and conservative substitutions are shown on a black or gray background, respectively. (C) Alignment of the deduced amino acid sequences of Cs4g11980 annotated in the CAP database and from cDNAs cloned in this work (CsSesquiTPS7). Identical amino acids and conservative substitutions are shown on a black or gray background, respectively. [file SupplementaryFigure2.TIF]
